# The burden of Parkinson’s disease, 1990–2021: a systematic analysis of the Global Burden of Disease study 2021

**DOI:** 10.3389/fnagi.2025.1596392

**Published:** 2025-06-16

**Authors:** Xi-Chen Wu, Yi-Yue Dong, Yu-Chen Ying, Guang-Yan Chen, Qian Fan, Ping Yin, Yue-Lai Chen

**Affiliations:** ^1^LongHua Hospital, Shanghai University of Traditional Chinese Medicine, Shanghai, China; ^2^Gansu Provincial People’s Hospital, Lanzhou, China

**Keywords:** Parkinson’s disease, Global Burden of Disease, incidence, prevalence, mortality, disability adjusted life years

## Abstract

**Background:**

The pathogenesis of Parkinson’s disease (PD) remains incompletely understood, has drawn significant attention within the scope of the Global Burden of Disease (GBD) study. Therefore, to explore PD’s global burden and devise countermeasures is indispensable.

**Methods:**

Data from GBD 2021 to analyze age standardized incidence rate (ASIR), prevalence rate (ASPR), mortality rate (ASMR), and disability adjusted life years (DALYs) rate (ASDR) burden of PD globally. Moreover, the estimated annual percentage change (EAPC) was utilized to gauge PD burden trends from 1990 to 2021. Subsequently, PD burden by sex and 21 GBD regions was further evaluated in 2021. Moreover, the influence of age, sex, and socio-demographic index (SDI) on burden of PD from 1990 to 2021 was examined. Finally, the projection of burden of PD from 2022 to 2026 was also conducted.

**Results:**

From 1990 to 2021, ASIR, ASPR, and ASDR were shown to be on an upward trend for both males and females. However, ASMR was higher in males than in females. In addition, ASIR, ASPR, and ASDR in the East Asian region were found to be the highest, and they were higher among males than among females. Notably, with the elapse of years, PD’s total incidence cases, prevalence cases, mortality cases, and DALYs cases were all presented with an upward trend. Moreover, in 1990 and 2021, ASDR in 75–79 age group was the highest. Finally, from 2022 to 2026, PD’s ASIR and ASPR were predicted to exhibit an upward tendency, while ASMR and ASDR were expected to show a downward trend.

**Conclusion:**

The research showed a growing global ASIR and ASPR of PD over time, urging more effective health policies to ease its burden.

## 1 Introduction

Parkinson’s disease (PD) is a clinicopathological syndrome characterised by retardation, tremor and dyskinesia associated with degeneration and loss of dopaminergic neurons due to aggregation of *α*-synuclein proteins (Lewy bodies) in the substantia nigra (Gibb and Lees, 1988). The global incidence of PD was 8–18 per 100,000 (Al-Kuraishy et al., 2023). The prevalence in people over 65 was 2% and increases with age (Hong et al., 2022). PD was about twice as prevalent in men as in women, and the incidence and prevalence of PD was slightly higher in the West than in the East (Yu and Wu, 2022; He et al., 2022). The number of patients was expected to increase to about 10 million worldwide by 2030 (GBD 2016 Parkinson’s Disease Collaborators, 2018). Currently, the diagnosis of PD was made by clinical presentation combined with laboratory tests and imaging (Li et al., 2022). Common treatments for PD were medications and surgery. Pharmacological interventions for PD encompass several classes of medications, including dopamine receptor agonists (e.g., pramipexole), monoamine oxidase B inhibitors (MAOBIs, e.g., selegiline), catechol-O-methyltransferase inhibitors (e.g., entacapone), anticholinergic agents (e.g., benztropine), and N-Methyl-D-aspartate (NMDA) receptor antagonists (e.g., amantadine). While these therapeutic agents demonstrate efficacy in ameliorating dyskinesia during the initial stages of PD, they were associated with the development of motor fluctuations and may exacerbate dyskinesia in advanced disease stages (Waller et al., 2021; Stevenson, 1997). Surgical treatments such as deep brain stimulation, although effective in improving symptoms, were difficult to generalize due to their complexity, high cost and a range of complications (Serva et al., 2022). The high cost and burden of clinical management of PD had become a serious global public health problem. Thus, it was imperative to assess the epidemiological trends of PD accurately and to thoroughly investigate the current disease burden of PD, which was essential to raise public awareness and increase knowledge among health policy makers.

The Global Burden of Disease (GBD) study was a systematic scientific effort to quantify the severity, risk factors, and intermediate clinical outcomes of all major diseases in a highly standardised way that allows comparisons across time, populations, and health problems (Murray, 2022). GBD study now provides annual estimates for 371 diseases and injuries, along with 3,499 clinical outcomes (sequelae) associated with these conditions, spanning 204 countries and regions, as well as sub-national units in over 20 countries since 1990 (Murray, 2022). Global epidemiological data from 1990 to 2019 demonstrate consistent increases in age-standardized rates (ASRs) for PD incidence, prevalence, and years lived with disability (YLDs), with estimated annual percentage changes (EAPCs) of 0.61, 0.52, and 0.53, respectively, particularly affecting individuals over 65 years and showing accelerated growth beyond 80 years, while the United States and Norway exhibited the most pronounced upward trends with EAPCs of 2.87 and 2.14 (Ou et al., 2021). However, approximately 30 countries have shown a declining trend in PD, particularly Italy and the Republic of Moldova (Ou et al., 2021). Currently, the most recent assessment of the global, regional and national burden of PD relies on GBD 2019 data, and a comprehensive analysis of the global burden of PD using the latest GBD 2021 data had not yet been conducted.

In summary, this study, based on the 2021 GBD database, analyzed the global burden of PD in terms of incidence, prevalence, mortality and Disability Adjusted Life Years (DALYs), and its association with various population stratification indicators, including time, age, gender, region, and the Socio-Demographic Index (SDI). The study also used historical data to project the global burden of PD for the next 5 years, from 2022 to 2026. The aim is to provide guidance for the epidemiological surveillance of Parkinson’s disease and to help determine the most appropriate public health intervention strategies.

## 2 Methods

### 2.1 Data acquisition

Within the framework of the International Classification of Diseases (ICD), specifically the 10th revision (ICD-10), classification codes corresponding to PD were G20-22 (Xu et al., 2024). In this study, the PD data analysed were taken from the GBD database.^1^ By clicking on the GBD Results Tool^2^ (accessed on October 25th, 2024), raw PD data for 204 countries worldwide from 1990 to 2021 were downloaded according to ICD codes. Although sub-national level (such as provincial and state-level) data existed for some large countries (e.g., China and India), only national-level data were included in this study to ensure the consistency and comparability of the datasets for 204 countries/regions worldwide. All of these data can be freely obtained via Global Health Data Exchange.^3^ This database primarily compiles statistics on the burden of 371 diseases and injuries, enabling us to understand the probability of a certain disease occurring in a specific time period and among a particular population during the period from 1990 to 2021 (GBD 2021 Diseases and Injuries Collaborators, 2024). Moreover, it accurately measured and presented key indicators such as incidence, prevalence, mortality, and DALYs of various diseases within different ages, sex, and geographical groups (Murray and GBD 2021 Collaborators, 2024). Information on the above estimated indices was provided in a comprehensive manner in the appendix of the GBD 2021 key document (GBD 2021 Tuberculosis Collaborators, 2024).

### 2.2 Demographic characteristics

The ages were grouped into 5-year intervals. Specifically, they were divided into the following groups: < 5, 5–9… 90–94, and 95 + years. The SDI is a holistic measure of the level of development of a country or region. It was made up of factors such as per capita income, years of education and the fertility rate of women under 25. If closer the SDI value was to 1, the higher the level of social and demographic advancement of the country or region, indicating a more ideal state in aspects such as economy, education, and population structure. Conversely, the closer the SDI value was to 0, the relatively lower the level of development, indicating that the country or region may be facing problems such as economic backwardness, inadequate educational resources and an irrational population structure. Based on SDI values, all nations or regions can be categorized into five levels of development: low (< 0.455), lower-middle (0.455–0.608), middle (0.608–0.690), high-middle (0.690–0.805) and high (> 0.805) (Fang et al., 2022). Based on similarities in epidemiology and geographical proximity, 204 countries (regions) were grouped into 21 GBD regions (GBD 2019 Diseases and Injuries Collaborators, 2020).

### 2.3 PD burden indicators

DALYs were used as an indicator to comprehensively measure the burden of disease [DALYs = YLDs + Years of Life Lost (YLLs)]. DALYs, incidence, prevalence and mortality, together with ASR [age-standardized DALYs rate (ASDR), age-standardized incidence rate (ASIR), age-standardized prevalence rate (ASPR), age-standardized mortality rate (ASMR)], and 95% uncertainty intervals (UI) were utilized to represent the burden of PD. The 95% UI was defined as the range of values within which the true value of the target parameter was expected to lie, commonly used in epidemiological, statistical, and medical research. Rates were reported per 100,000 population. In each calculation step, the UI was according to the 2.5th and 97.5th percentiles of 1,000 drawn-level estimates. The EAPC was a holistic metric designed to measure changing long-term trends in the ASR of disease burden. Formal statistical comparison of EAPC across groups was not a standard practice, and it was calculated using the generalised linear regression model lnY = *α* + *β*T + *ϵ*. In this model, Y was the quantity or ratio, Yis the ASR, T is the calendar year, β was the regression coefficient, and ϵ was the error term (Cen et al., 2024). The EAPC was expressed as 100 × (exp[β] − 1), with a 95% confidence interval (CI) (Cen et al., 2024). EAPC was frequently used in epidemiological, statistical, and public health research to describe the average annual change trend of an indicator over a period. An upward trend of the indicator was indicated when EAPC > 0, a downward trend was shown when EAPC < 0, and no significant trend change of the indicator was observed when EAPC = 0. 95% CI was not referred to as the probability that the population parameter fell within the interval being 95%, but rather that if sampling were repeated multiple times (e.g., 100 times), the confidence intervals calculated would contain the true population parameter approximately 95 times. Overlapping confidence intervals were indicative of no statistically significant differences between groups (McCormack et al., 2013). The coefficients of the linear regression model fitted using Ordinary Least Squares (OLS) were used to estimate the significance of each indicator from 1990 to 2021. A *p*-value less than 0.05 was indicated to be statistically significant.

### 2.4 Statistical analysis

To obtain the disease burden of PD in 204 countries at the global level in 1990 and 2021, the ASIR, ASPR, ASMR, and ASDR of PD data were extracted based on the “dplyr” package (v 1.1.4) (Mangiola et al., 2021). Subsequently, a world map covering 204 countries was created for visualization through “ggplot2” package (v 3.4.1) (Gustavsson et al., 2022)^.^ In addition, the “flextable” package (v 0.9.6)^4^ was also utilized to create tables to display ASIR, ASPR, ASMR, and ASDR of PD in different sexes, classified by 21 regions and SDI in 1990 and 2021. And EAPC values were calculated to understand the trends of burden indicators for PD in the context of temporal changes. We then examined the burden of PD at the level of the 21 regions in 2021. The “ggplot2” package (v 3.4.1) was utilized to generate histograms, which facilitated comparison of incidence, prevalence, mortality, DALYs, and ASIR, ASPR, ASMR, and ASDR between different sex. Next, in order to understand the burden of PD at different SDI levels, “ggplot2” package (v 3.4.1) was utilized to draw bar charts to compare EAPC changes trends of ASIR, ASPR, ASMR and ASDR globally and in the five SDI regions. Furthermore, the “ggplot2” package (v 3.4.1) was also employed to generate a stacked area chart. The aim was to describe the trends of changes in total incidence cases, prevalence cases, mortality cases and DALYs cases of PD globally and in five SDI regions from 1990 to 2021. Besides, with the objective of understanding with respect to SDI and PD’s disease burden association in different regions during the period from 1990 to 2021, “cor” package (v 0.8.3) (Verhaegen et al., 2024) was utilized to conduct Spearman correlation analysis method for evaluating correlations between SDI and ASIR, ASPR, ASMR, and ASDR under conditions of 21 GBD regions (|correlation (cor)| > 0.3, *p* < 0.05). The influence of both age and sex on the burden of PD in patients in 1990 and 2021 was also examined. Histograms were generated by “ggplot2” package (v 3.4.1) to facilitate comparison of differences and trends in incidence, prevalence, mortality, and DALYs from 1990 to 2021, across different sex and age groups. Finally, the “forecast” package (v 8.23.0)^5^ and “tseries” package (v 0.10–58)^6^ were employed in construction of autoregressive integrated moving average (ARIMA) model, and model was assessed through a white noise test. The auto. arima function from the “forecast” package (v 8.23.0) was used to identify an optimized ARIMA model that minimizes both the akaike information criterion (AIC) and the bayes information criterion (BIC). The AIC was used to measure the goodness of fit of the model while taking the model’s complexity into account. A smaller AIC value was considered to indicate a better fitting effect of the model. The BIC, similar to AIC, imposed a stricter penalty on model complexity. A smaller BIC value was regarded as suggesting a better fitting effect of the model. Then ARIMA model was applied to predict PD’s ASIR, ASPR, ASMR and ASDR in next 5 years, with statistical analysis incorporating 95% CI. First, the raw GBD data were read, and then the ASIR, ASPR, ASDR, and ASMR data were extracted. The ARIMA_Plot function was used to perform ARIMA modeling and forecasting. Prediction results were presented using the “ggplot2” package (v 3.4.1). Statistical analyses were performed using R language (v 4.2.2), and results were considered statistically significant if the corresponding *p* < 0.05 (Xu et al., 2024). Moreover, the model’s predictive performance was evaluated through the Mean Error (ME), Root Mean Squared Error (RMSE), Mean Absolute Error (MAE), Mean Percentage Error (MPE), Mean Absolute Percentage Error (MAPE), Mean Absolute Scaled Error (MASE), and Autocorrelation Function at Lag 1 (ACF1). Smaller values of MAE, RMSE, MAPE, and MASE were indicative of better predictive performance of the model. For ME, MPE, and ACF1, the ideal values were close to 0, and the closer the predicted values were to the actual values, the higher the predictive performance became. The rationality of the model assumptions was verified through residual normality tests, and a Q-Q plot for residual normality of the ARIMA model was drawn. The Box.test function was used to conduct the Ljung-Box test to examine whether all autocorrelation coefficients of the residuals were zero. If the overall correlation coefficient was zero, it indicated white noise with no autocorrelation.

## 3 Results

### 3.1 The burden of PD in 204 countries

**Figure 1:**
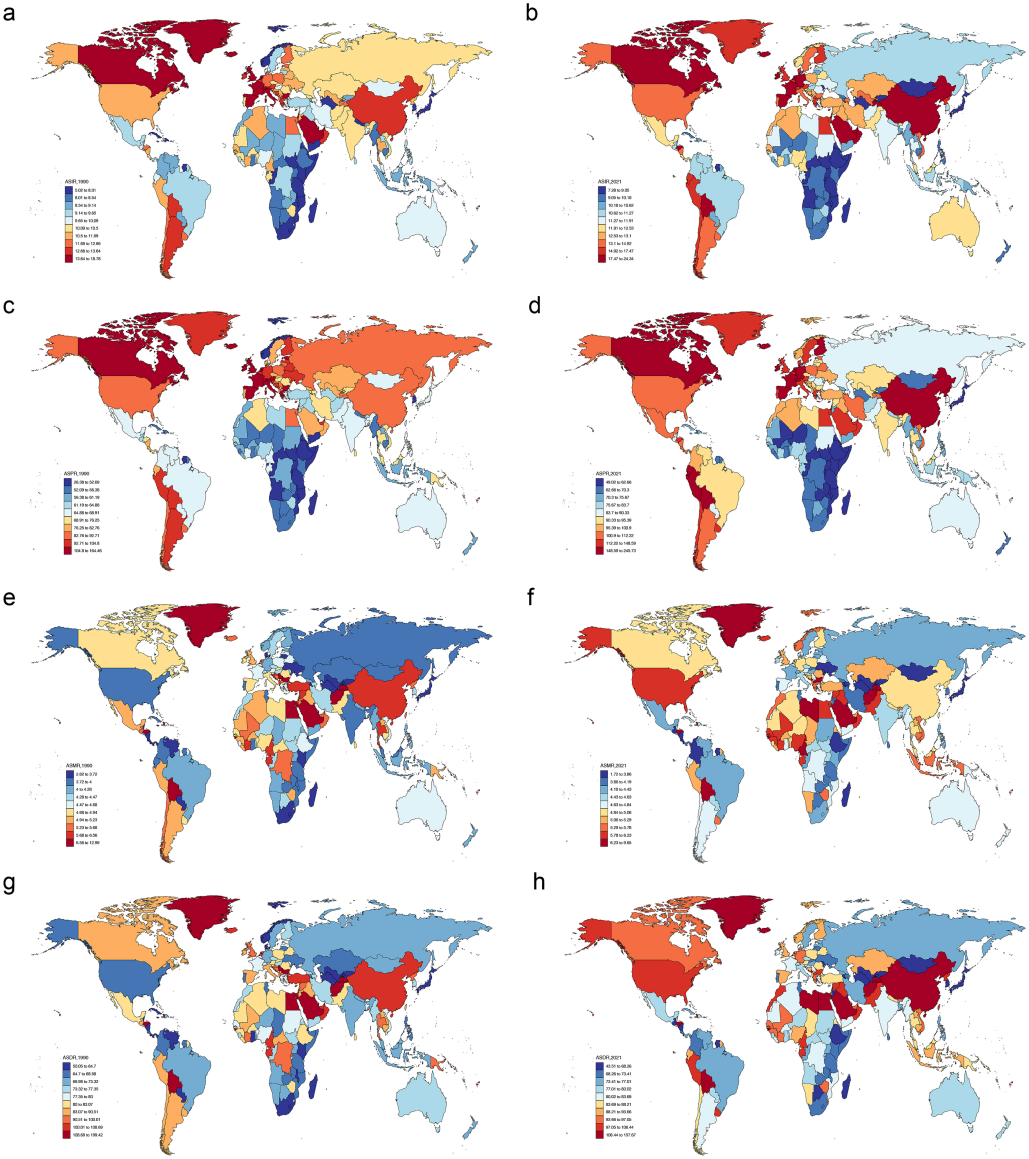
Age-standardized rates of PD burden in different countries in 1990 and 2021. ASIR in 1990 **(a)**, ASIR in 2021 **(b)**, ASPR in 1990 **(c)**, ASPR in 2021 **(d)**, ASMR in 1990 **(e)**, ASMR in 2021 **(f)**, ASDR in 1990 **(g)**, ASDR in 2021 **(h)**. PD, Parkinson’s disease; ASIR, age-standardized incidence rate; ASPR, age-standardized prevalence rate; ASMR, age-standardized mortality rate; ASDR, age-standardized DALYs rate; DALYs, disability-adjusted life years.

Significant variations in ASIR, ASPR, ASMR, and ASDR for PD were observed across different countries. Specifically, in 1990, the country with the highest ASIR was the Netherlands 18.78 per 100,000 (95% UI = 16.77–20.45), followed by Israel, with 18.45 per 100,000 (95% UI = 16.07–21.73). In 2021, country with the highest ASIR was China, with 24.34 per 100,000 (95% UI = 20.67–28.30) (Figures 1a,b; Supplementary Tables S1, S2). Regarding ASPR, in 1990, the top three countries were Israel, with 164.48 per 100,000 (95% UI = 138.47–199.54), Italy with 160.72 per 100,000 (95% UI = 136.22–187.75), and Netherlands with 162.18 per 100,000 (95% UI = 143.17 to 181.79). Surprisingly, the country with the highest ASPR in 2021 was still China with 245.73 per 100,000 (95% UI = 208.28 to 289.24) (Figures 1c,d; Supplementary Tables S3, S4). In addition, the countries with the highest ASMR in 1990 and 2021 were Qatar with 12.99 per 100,000 (95% UI = 11.17 to 14.9) and Honduras with 9.65 per 100,000 (95% UI = 7.76 to 11.65), respectively (Figures 1e,f; Supplementary Tables S5, S6). For ASDR, the top countries in 1990 and 2021 t were still Qatar with 199.42 per 100,000 (95% UI = 173.24 to 227.31) and Honduras with 157.67 per 100,000 (95% UI = 131.01 to 188.02), respectively (Figures 1g,h; Supplementary Tables S7, S8). Notably, from 1990 to 2021, ASIR, ASPR, and ASDR of PD in China displayed an increasing tendency, while ASMR presented a downward trend, indicating that both therapeutic approaches for PD and comprehensive patient care had been achieved, but the increase in indicators such as ASIR also indicated that more efforts were needed in aspects such as disease prevention, early screening and etiology research. There was a need to further explore how to reduce the incidence of PD at the source and improve the health of the population as a whole to better address the many problems caused by the disease.

### 3.2 Trends in the GBD of PD from 1990 to 2021

Based on the GBD database, among the male population, from 1990 to 2021, globally, ASIR of PD climbed at a pace of 1.11% by the year (95% CI = 1.09–1.13, *p* < 0.05); ASPR climbed at a pace of 1.70% each year (95% CI = 1.67–1.73, *p* < 0.05); ASMR climbed at a pace of 0.21% annually (95% CI = 0.14–0.28, *p* < 0.05); and ASDR climbed at a pace of 0.37% annually (95% CI = 0.31–0.42, *p* < 0.05). When analyzed by different SDI levels, Middle SDI region had the fastest growth rates for ASIR and ASPR. ASIR increased by 1.57% (95% CI = 1.53–1.61, *p* < 0.05) and ASPR grew by 2.50% (95% CI = 2.45–2.55, *p* < 0.05). For ASMR and ASDR, Low - middle SDI region had the fastest growth rates, with an grow of 0.61% (95% CI = 0.53–0.70, *p* < 0.05) and 0.56% (95% CI = 0.51–0.61, *p* < 0.05), respectively (Table 1). Additionally, in the female population, globally, the ASIR of PD experienced an increase of 0.93% per year (95% CI = 0.91–0.95, *p* < 0.05); ASPR experienced an increase at a rate of 1.25% per year (95% CI = 1.23 to 1.27, *p* < 0.05); while ASMR witnessed a 0.01% decline per annum (95% CI = − 0.04 to 0.03, *p* = 0.79); and ASDR witnessed a 0.14% growth per annum (95% CI = 0.11 to 0.17, *p* < 0.05). Among different SDI regions, Middle SDI region also demonstrated the most rapid growth rates for ASIR and ASPR. ASIR escalated by 1.31% (95% CI = 1.28 to 1.35, *p* < 0.05) and ASPR escalated by 2.02% (95% CI = 1.97 to 2.07, *p* < 0.05). In the middle SDI region, ASMR decreased at a rate of 0.57% per year (95% CI = − 0.61 to - 0.52, *p* < 0.05). The growth rates of ASDR in High SDI and Low - middle SDI regions were approximately the same, at around 0.38% (Table 2). Overall, both ASIR and ASPR were on the rise among both males and females, while ASMR of females was decreasing. Notably, ASDR for males exceeded that of females, which indicated that burden of PD was higher in males.

**Table 1:** Global and SDI-level trends in PD burden indicators among males, 1990–2021.

Location	1990_ASPR^1^	2021_ASPR	EAPC^2^_ASPR	*p*_value_ASPR	1990_ASIR^3^	2021_ASIR	EAPC_ASIR	*p*_value_ASIR	1990_ASMR^4^	2021_ASMR	EAPC_ASMR	*p*_value_ASMR	1990_ASDR^5^	2021_ASDR	EAPC_ASDR	*p*_value_ASDR
Andean Latin America	113.97 (97.23 to 132.89)	204.06 (170.64 to 247.12)	1.8917% (1.85274 to 1.93068)	0	13.62 (12.3 to 14.99)	20.86 (18.63 to 23.85)	1.40011% (1.383 to 1.41722)	0	6.43 (5.54 to 7.33)	6.78 (5.61 to 8.12)	0.3396% (0.2398 to 0.43949)	0	112.86 (97.9 to 126.47)	126.8 (106.49 to 149.67)	0.46672% (0.38832 to 0.54519)	0
Australasia	77.66 (65.79 to 90.4)	102.32 (88.93 to 120.05)	0.92056% (0.81921 to 1.02202)	0	12.98 (11.87 to 13.97)	15.42 (14.2 to 17.05)	0.58981% (0.53204 to 0.64763)	0	7.08 (6.64 to 7.34)	6.88 (6.17 to 7.32)	0.02467% (−0.11405 to 0.16359)	0.72997	111.82 (106.62 to 117.16)	109.75 (100.33 to 117.2)	0.03214% (−0.09334 to 0.15778)	0.61945
Caribbean	64.12 (55.32 to 73.69)	84.12 (73.65 to 97.1)	0.83921% (0.76128 to 0.91719)	0	9.92 (9.25 to 10.56)	11.79 (10.9 to 12.68)	0.51725% (0.47829 to 0.55622)	0	5.69 (5.33 to 6.02)	6.11 (5.44 to 6.8)	0.27311% (0.20568 to 0.34058)	0	93.76 (87.78 to 99.91)	103.48 (92.71 to 114.58)	0.36317% (0.31334 to 0.41303)	0
Central Asia	96.14 (79.92 to 111.89)	100.61 (83.6 to 119.36)	0.25889% (0.14803 to 0.36988)	8e-05	13.82 (12.47 to 15.14)	15.3 (13.84 to 16.69)	0.40093% (0.35375 to 0.44813)	0	5.5 (4.78 to 6.93)	5.53 (5.02 to 5.98)	−0.04068% (−0.13249 to 0.05123)	0.39244	95.2 (84.15 to 114.95)	94.68 (86.2 to 104.06)	−0.07753% (−0.15871 to 0.00373)	0.07125
Central Europe	104.66 (91.17 to 118.18)	115.89 (102.38 to 129.26)	0.34555% (0.30103 to 0.39008)	0	14.76 (13.53 to 15.97)	15.52 (14.36 to 16.76)	0.17527% (0.13965 to 0.2109)	0	6.83 (6.53 to 7.02)	6.59 (6.07 to 6.99)	−0.05841% (−0.08363 to −0.03319)	9e-05	113.53 (108.01 to 118.75)	111.18 (103 to 119.32)	−0.04167% (−0.06235 to −0.02099)	0.00044
Central Latin America	79.02 (68.96 to 90.28)	118.83 (104.77 to 134.83)	0.99906% (0.87814 to 1.12012)	0	10.92 (10.02 to 11.93)	14.48 (13.24 to 15.8)	0.67629% (0.59318 to 0.75948)	0	5.45 (5.13 to 5.61)	5.53 (4.86 to 6.11)	−0.01827% (−0.09989 to 0.06343)	0.66425	91.2 (86.66 to 95.37)	98.65 (88.12 to 110.09)	0.13133% (0.06844 to 0.19425)	0.00029
Central Sub-Saharan Africa	65.07 (52.84 to 78.93)	75.3 (60.87 to 91.45)	0.44467% (0.36008 to 0.52933)	0	10.48 (9.37 to 11.57)	11.31 (10.12 to 12.6)	0.24729% (0.20053 to 0.29408)	0	5.58 (4.41 to 7.14)	5.56 (4.16 to 7.9)	−0.03673% (−0.16344 to 0.09014)	0.57445	96.14 (78.64 to 117.84)	94.75 (73.56 to 126.1)	−0.07441% (−0.19306 to 0.04437)	0.22893
East Asia	104.9 (87.35 to 125.04)	302.81 (256.31 to 353.52)	3.45187% (3.31772 to 3.58618)	0	16.35 (14.03 to 18.78)	31.01 (26.47 to 36.1)	2.21593% (2.16234 to 2.26954)	0	8.15 (6.97 to 9.24)	7.24 (5.83 to 8.83)	−0.44606% (−0.63808 to −0.25367)	9e-05	132.66 (113.18 to 149.5)	143.97 (120.68 to 171.49)	0.22313% (0.0885 to 0.35795)	0.00285
Eastern Europe	108.15 (90.76 to 129.09)	115.33 (96.74 to 136.72)	0.14299% (0.00242 to 0.28375)	0.05533	13.75 (11.93 to 15.73)	14.33 (12.46 to 16.18)	0.06851% (−0.01989 to 0.15698)	0.13927	5.79 (5.48 to 6)	5.51 (4.96 to 6.05)	−0.25158% (−0.34458 to −0.1585)	1e-05	99.35 (93.25 to 106.2)	97.94 (88.73 to 108.3)	−0.09172% (−0.16017 to −0.02322)	0.01353
Eastern Sub-Saharan Africa	62.9 (53.09 to 73.33)	74.47 (64.02 to 84.87)	0.52281% (0.48107 to 0.56456)	0	10.33 (9.32 to 11.34)	11.41 (10.34 to 12.53)	0.31134% (0.29057 to 0.33211)	0	4.76 (3.83 to 6.19)	4.68 (3.63 to 6.9)	−0.1641% (−0.20989 to −0.11828)	0	83.83 (69.58 to 101.85)	81.37 (65.38 to 112.33)	−0.2166% (−0.25789 to −0.17528)	0
Global	99.22 (86.7 to 113.81)	168.24 (148.41 to 191.71)	1.701% (1.67372 to 1.72829)	0	14.13 (12.59 to 15.75)	19.72 (17.69 to 21.88)	1.11037% (1.08714 to 1.13359)	0	6.28 (5.75 to 6.82)	6.57 (5.93 to 7.14)	0.21014% (0.13626 to 0.28407)	0	105.99 (97.61 to 114.91)	117.47 (106.15 to 128.88)	0.36502% (0.30826 to 0.4218)	0
High SDI	112.94 (100.31 to 128.02)	154.34 (142.25 to 168.23)	1.04012% (0.97294 to 1.10735)	0	15.18 (13.74 to 16.76)	19.43 (18.09 to 20.87)	0.82511% (0.78542 to 0.86482)	0	6.33 (5.9 to 6.54)	6.92 (6.22 to 7.3)	0.44033% (0.32771 to 0.55307)	0	105.94 (99.75 to 111.52)	118.74 (109.27 to 126.88)	0.48917% (0.39335 to 0.58509)	0
High-income Asia Pacific	75.01 (63.53 to 89.05)	70.34 (60.65 to 80.99)	0.47375% (0.29099 to 0.65684)	2e-05	10.35 (8.96 to 11.86)	10.65 (9.54 to 11.82)	0.44818% (0.36176 to 0.53467)	0	4.86 (4.48 to 5.06)	5 (4.44 to 5.3)	0.35471% (0.24316 to 0.46638)	0	80.26 (75.31 to 84.81)	80.58 (73.82 to 86.48)	0.34613% (0.23213 to 0.46027)	0
High-income North America	117.4 (99.62 to 137.93)	161.05 (150.7 to 172.47)	0.88989% (0.80432 to 0.97552)	0	15.64 (13.52 to 17.78)	20.43 (18.89 to 22.04)	0.75522% (0.68294 to 0.82755)	0	5.63 (5.14 to 5.85)	8.51 (7.49 to 9.01)	1.37645% (1.19484 to 1.55839)	0	97.05 (90.32 to 103.37)	140.49 (127.53 to 149.57)	1.19095% (1.04753 to 1.33458)	0
High-middle SDI	120.85 (106.48 to 138.46)	219.33 (191.52 to 253.8)	1.88856% (1.85122 to 1.92592)	0	16.51 (14.84 to 18.33)	24.15 (21.24 to 27.51)	1.25562% (1.2203 to 1.29095)	0	7.38 (6.78 to 7.84)	6.81 (6.02 to 7.67)	−0.26657% (−0.36168 to −0.17137)	1e-05	123.22 (113.61 to 132.39)	126.98 (112.23 to 144.01)	0.07528% (0.00322 to 0.14739)	0.04944
Low SDI	65.73 (55.81 to 76.42)	83.24 (71.47 to 95.57)	0.77303% (0.69799 to 0.84813)	0	10.87 (9.81 to 12.03)	12.25 (11.02 to 13.5)	0.39663% (0.35196 to 0.44131)	0	5.38 (4.41 to 6.98)	5.76 (4.73 to 7.16)	0.45511% (0.29316 to 0.61732)	1e-05	92.26 (77.12 to 118.22)	96.92 (80.72 to 118.57)	0.3034% (0.18604 to 0.42089)	2e-05
Low-middle SDI	71.76 (60.92 to 83.02)	101.78 (88.07 to 118.45)	1.14837% (1.08666 to 1.21011)	0	11.44 (10.13 to 12.77)	13.94 (12.51 to 15.35)	0.67135% (0.62907 to 0.71365)	0	4.97 (4.15 to 6.21)	5.89 (4.94 to 6.84)	0.61487% (0.53099 to 0.69882)	0	86.33 (73.68 to 106.34)	102.22 (86.41 to 116.4)	0.56243% (0.51013 to 0.61475)	0
Middle SDI	85.9 (72.92 to 101.05)	187.41 (161.17 to 217.36)	2.50085% (2.44718 to 2.55454)	0	13.13 (11.43 to 14.77)	20.91 (18.19 to 23.6)	1.56652% (1.52663 to 1.60642)	0	6.08 (5.5 to 6.74)	6.3 (5.54 to 7.12)	0.10382% (0.01777 to 0.18993)	0.02469	103.09 (92.12 to 113.65)	117.4 (103.33 to 132.73)	0.39148% (0.32848 to 0.45452)	0
North Africa and Middle East	81.79 (68.95 to 95)	126.11 (107.84 to 144.29)	1.44921% (1.39472 to 1.50374)	0	12.9 (11.74 to 14.18)	16.83 (15.21 to 18.53)	0.88983% (0.85055 to 0.92912)	0	6.94 (6.11 to 7.92)	6.18 (5.44 to 6.86)	−0.4098% (−0.48018 to −0.33937)	0	113.03 (101.72 to 127.68)	106.46 (95.01 to 117.38)	−0.22188% (−0.28315 to −0.16056)	0
Oceania	84.03 (69.73 to 101.31)	93.08 (75.05 to 109.31)	0.29191% (0.21256 to 0.37133)	0	12.52 (11.14 to 14.27)	13.06 (11.43 to 14.87)	0.14659% (0.09385 to 0.19935)	1e-05	6.31 (5.05 to 8.05)	5.52 (4.3 to 7.49)	−0.49287% (−0.56615 to −0.41954)	0	113 (92.05 to 139.08)	100.76 (79.86 to 131.13)	−0.42197% (−0.4918 to −0.3521)	0
South Asia	68.96 (57.42 to 81.02)	98.15 (82.78 to 115.3)	1.15025% (1.05348 to 1.24711)	0	11.42 (9.86 to 13.02)	13.45 (11.79 to 15.16)	0.54455% (0.48373 to 0.60541)	0	4.76 (3.72 to 6.44)	5.75 (4.46 to 7.04)	0.69717% (0.54789 to 0.84666)	0	83.22 (66.7 to 109.55)	98.88 (78.59 to 118.07)	0.56931% (0.47046 to 0.66825)	0
Southeast Asia	75.38 (65.16 to 87.23)	102.92 (90.95 to 117.09)	0.91867% (0.84844 to 0.98896)	0	11.21 (10.21 to 12.29)	13.82 (12.65 to 15.05)	0.659% (0.61631 to 0.70172)	0	5.1 (4.44 to 6.01)	6.28 (5.48 to 7.35)	0.66024% (0.56889 to 0.75166)	0	88.06 (78.19 to 100.31)	108.18 (96.09 to 124.6)	0.63948% (0.56665 to 0.71237)	0
Southern Latin America	120.68 (104.56 to 134.08)	138.22 (116.87 to 166.95)	0.61762% (0.50088 to 0.73449)	0	17.74 (16.14 to 18.99)	19.26 (17.67 to 21.52)	0.42491% (0.35893 to 0.49093)	0	7.87 (7.45 to 8.17)	7.58 (7 to 8.02)	0.12504% (0.01572 to 0.23449)	0.03251	129.95 (122.67 to 137.03)	126.05 (117.04 to 134.68)	0.1066% (0.01789 to 0.19538)	0.02523
Southern Sub-Saharan Africa	72.02 (61.13 to 84.63)	84.32 (71.19 to 98.38)	0.36625% (0.31107 to 0.42147)	0	10.73 (9.45 to 12.14)	12.34 (10.83 to 13.85)	0.36788% (0.34065 to 0.39512)	0	4.3 (3.73 to 5.28)	5.74 (5.22 to 6.15)	0.95903% (0.60829 to 1.31098)	1e-05	72.74 (63.39 to 87.37)	96.2 (87.38 to 103.48)	0.86585% (0.54083 to 1.19193)	1e-05
Tropical Latin America	82.98 (70.06 to 98.56)	103.07 (87.51 to 122.65)	0.94501% (0.8487 to 1.04141)	0	11.14 (9.51 to 12.76)	12.46 (10.81 to 14.05)	0.54376% (0.48111 to 0.60645)	0	5.37 (4.94 to 5.6)	5.38 (4.87 to 5.7)	0.28663% (0.21064 to 0.36268)	0	90.62 (84.84 to 95.78)	93.65 (86.03 to 100.4)	0.33157% (0.27126 to 0.39192)	0
Western Europe	142.99 (129.85 to 157.39)	180.27 (163.5 to 200.34)	0.65185% (0.53979 to 0.76403)	0	18.52 (17.14 to 19.93)	22.12 (20.63 to 23.7)	0.52446% (0.44907 to 0.59991)	0	7.31 (6.84 to 7.53)	7.01 (6.26 to 7.4)	0.10959% (0.02526 to 0.19399)	0.01622	121.93 (114.73 to 128.77)	121.89 (111.44 to 131.48)	0.17495% (0.09341 to 0.25655)	0.00022
Western Sub-Saharan Africa	69.63 (59.38 to 80.14)	84.38 (72.94 to 96.78)	0.71872% (0.65022 to 0.78727)	0	11.24 (10.19 to 12.4)	13.16 (11.98 to 14.47)	0.56312% (0.527 to 0.59926)	0	5.71 (4.81 to 6.73)	6.3 (5.2 to 7.36)	0.48577% (0.41035 to 0.56124)	0	92.58 (80.06 to 108.42)	101.74 (84.93 to 119.6)	0.45635% (0.39003 to 0.52271)	0

^1^ASPR: Age Standardized Prevalence Rate.

^2^EAPC: Estimated Annual Percentage Chang.

^3^ASIR: Age Standardized Incidence Rate.

^4^ASMR: Age Standardized Mortality Rate.

^5^ASDR: Age Standardized Disability Adjusted Life Years Rate.

**Table 2:** Global and SDI-level trends in PD burden indicators among females, 1990–2021.

Location	1990_ASPR	2021_ASPR	EAPC_ASPR	*p*_value_ASPR	1990_ASIR	2021_ASIR	EAPC_ASIR	*p*_value_ASIR	1990_ASMR	2021_ASMR	EAPC_ASMR	*p*_value_ASMR	1990_ASDR	2021_ASDR	EAPC_ASDR	*p*_value_ASDR
Andean Latin America	68.94 (58.25 to 82.03)	118.91 (100.25 to 139.05)	1.64808% (1.58976 to 1.70644)	0	8.98 (8.1 to 10.02)	13.04 (11.67 to 14.41)	1.16161% (1.1343 to 1.18893)	0	3.91 (3.4 to 4.44)	3.88 (3.18 to 4.63)	−0.03689% (−0.1608 to 0.08717)	0.56419	65.85 (58.24 to 73.77)	72.35 (61.54 to 84.03)	0.22552% (0.12409 to 0.32705)	0.00014
Australasia	56.57 (47.49 to 67.59)	72.98 (57.59 to 91.64)	0.83749% (0.77863 to 0.89638)	0	7.36 (6.51 to 8.15)	8.76 (7.46 to 10.31)	0.6177% (0.56771 to 0.66772)	0	3.02 (2.66 to 3.23)	2.93 (2.38 to 3.24)	−0.01711% (−0.12453 to 0.09042)	0.75715	51.49 (46.53 to 55.85)	51.28 (43.89 to 56.64)	0.02634% (−0.06041 to 0.11317)	0.5563
Caribbean	48.85 (42.07 to 56.12)	65.56 (56.72 to 74.41)	0.85061% (0.75501 to 0.9463)	0	6.83 (6.29 to 7.37)	8.28 (7.7 to 8.91)	0.59038% (0.52994 to 0.65085)	0	3.38 (3.1 to 3.65)	3.46 (3.03 to 3.88)	0.16579% (0.09218 to 0.23946)	0.00012	56.09 (51.93 to 61.05)	60.07 (53.57 to 66.92)	0.26399% (0.20772 to 0.32029)	0
Central Asia	67.03 (56.32 to 79.12)	76.68 (66.43 to 86.38)	0.35282% (0.2729 to 0.43279)	0	8.38 (7.46 to 9.3)	10.22 (9.4 to 10.9)	0.61861% (0.57672 to 0.66051)	0	2.96 (2.57 to 3.4)	3.23 (2.89 to 3.54)	0.3039% (0.17529 to 0.43268)	7e-05	54.32 (48.23 to 60.82)	59.07 (53.84 to 64.54)	0.21948% (0.11402 to 0.32505)	0.00031
Central Europe	75.38 (67.06 to 84.02)	81.85 (73.77 to 89.73)	0.15551% (0.1153 to 0.19573)	0	9.57 (8.9 to 10.26)	9.89 (9.18 to 10.55)	0.03357% (0.00483 to 0.06233)	0.02928	3.82 (3.56 to 3.98)	3.93 (3.53 to 4.26)	−0.00313% (−0.06767 to 0.06145)	0.92492	66.84 (62.7 to 70.81)	67.98 (62.05 to 73.09)	−0.04916% (−0.09594 to −0.00236)	0.04828
Central Latin America	57.31 (50.29 to 65.33)	82.85 (72.67 to 94.75)	0.95814% (0.83066 to 1.08578)	0	7.78 (7.08 to 8.53)	9.86 (8.93 to 10.89)	0.66844% (0.58437 to 0.75258)	0	3.71 (3.43 to 3.87)	3.25 (2.82 to 3.58)	−0.3822% (−0.46228 to −0.30205)	0	59.98 (56.34 to 63.4)	59.02 (52.55 to 65.94)	−0.08326% (−0.13668 to −0.02982)	0.00471
Central Sub-Saharan Africa	40.15 (32.92 to 48.44)	51.04 (41.75 to 61.73)	0.78262% (0.65151 to 0.91389)	0	5.68 (5.13 to 6.4)	6.63 (5.88 to 7.45)	0.5514% (0.46712 to 0.63575)	0	3.47 (2.72 to 4.51)	3.74 (2.54 to 4.94)	0.30242% (0.20669 to 0.39824)	0	59.69 (47.85 to 76.84)	64.37 (46.64 to 81.94)	0.304% (0.21269 to 0.39539)	0
East Asia	82.52 (69.04 to 98.45)	194.81 (166.9 to 229.02)	2.77316% (2.66158 to 2.88487)	0	10.47 (8.93 to 12.02)	18.63 (15.85 to 21.51)	1.91047% (1.86362 to 1.95735)	0	4.89 (4 to 5.68)	3.59 (2.82 to 4.44)	−1.21771% (−1.35308 to −1.08216)	0	87.16 (72.88 to 100.04)	80.8 (66.16 to 95.98)	−0.4191% (−0.50505 to −0.33308)	0
Eastern Europe	83.33 (70.32 to 98.57)	79.82 (67.55 to 93.16)	−0.2254% (−0.34391 to −0.10674)	0.00082	9.44 (8.13 to 10.85)	9.14 (7.97 to 10.35)	−0.19288% (−0.26903 to −0.11668)	3e-05	3.27 (3.02 to 3.45)	3.62 (3.24 to 3.99)	−0.05671% (−0.20275 to 0.08954)	0.45298	60.84 (56.12 to 65.9)	64.57 (58.06 to 71.13)	−0.12344% (−0.23515 to −0.01162)	0.0386
Eastern Sub-Saharan Africa	38.03 (32.44 to 44.58)	46.56 (39.86 to 53.61)	0.587% (0.53599 to 0.63805)	0	5.69 (5.12 to 6.3)	6.49 (5.86 to 7.13)	0.4277% (0.39513 to 0.46027)	0	3.41 (2.41 to 4.38)	3.59 (2.48 to 4.73)	0.13426% (0.10326 to 0.16526)	0	57.7 (43.76 to 71.57)	59.71 (42.99 to 75.87)	0.06119% (0.02845 to 0.09394)	0.00095
Global	77.09 (67.9 to 88.09)	114.47 (102.05 to 129.78)	1.2532% (1.23238 to 1.27403)	0	9.29 (8.3 to 10.35)	12.36 (11.11 to 13.75)	0.9316% (0.91404 to 0.94916)	0	3.62 (3.24 to 3.95)	3.59 (3.06 to 3.94)	−0.00515% (−0.04206 to 0.03177)	0.78643	65.37 (59.4 to 70.88)	68.56 (60.9 to 75.76)	0.13932% (0.11106 to 0.16758)	0
High SDI	77.51 (69.58 to 86.81)	101.65 (93.57 to 109.89)	0.90498% (0.84889 to 0.96111)	0	9.06 (8.2 to 9.95)	11.45 (10.69 to 12.22)	0.77691% (0.74651 to 0.80731)	0	3.11 (2.74 to 3.29)	3.25 (2.67 to 3.56)	0.33243% (0.25403 to 0.4109)	0	56.01 (50.84 to 60.29)	60.44 (52.47 to 66.26)	0.38172% (0.31501 to 0.44847)	0
High-income Asia Pacific	56.44 (48.01 to 66.53)	53.28 (46.06 to 61.74)	0.24915% (0.06002 to 0.43863)	0.01492	6.42 (5.55 to 7.42)	6.5 (5.82 to 7.22)	0.34027% (0.23337 to 0.44729)	0	2.45 (2.12 to 2.7)	2.62 (2 to 3.01)	0.48311% (0.37184 to 0.5945)	0	44.35 (39.58 to 48.79)	45.47 (37.56 to 51.16)	0.36112% (0.23647 to 0.48592)	0
High-income North America	72.1 (61.44 to 84.8)	89.26 (82.88 to 95.63)	0.68992% (0.64763 to 0.73224)	0	8.74 (7.5 to 10.02)	10.52 (9.78 to 11.3)	0.58326% (0.54654 to 0.61999)	0	2.97 (2.53 to 3.18)	3.75 (3.09 to 4.08)	0.89502% (0.82121 to 0.96888)	0	52.9 (47.18 to 57.4)	64.61 (56.03 to 70.28)	0.74238% (0.69119 to 0.79359)	0
High-middle SDI	92.18 (80.57 to 106.29)	138.48 (121.15 to 159.63)	1.17396% (1.1285 to 1.21943)	0	10.5 (9.4 to 11.7)	14.25 (12.56 to 16.1)	0.9063% (0.87739 to 0.93521)	0	3.8 (3.41 to 4.1)	3.59 (3.05 to 4.03)	−0.317% (−0.38758 to −0.24637)	0	70.29 (63.68 to 76.38)	71.73 (62.97 to 80.26)	−0.08326% (−0.14593 to −0.02055)	0.01426
Low SDI	49.06 (42.01 to 57.12)	63.42 (54.7 to 73.11)	0.94517% (0.8684 to 1.02199)	0	7.35 (6.6 to 8.19)	8.38 (7.55 to 9.22)	0.51303% (0.45727 to 0.56883)	0	3.89 (2.93 to 4.94)	4.19 (3.26 to 5.15)	0.38934% (0.22673 to 0.55222)	5e-05	65.83 (52.87 to 80.18)	70.73 (57.18 to 84.34)	0.34798% (0.2269 to 0.46921)	0
Low-middle SDI	59.94 (51.52 to 69.76)	83.38 (71.79 to 96.45)	1.24277% (1.18167 to 1.3039)	0	8.5 (7.53 to 9.51)	10.25 (9.13 to 11.33)	0.73591% (0.68877 to 0.78306)	0	3.78 (2.98 to 4.73)	4.09 (3.44 to 4.76)	0.34914% (0.26598 to 0.43238)	0	65.76 (53.92 to 79.03)	72.63 (62.62 to 83.67)	0.38335% (0.32671 to 0.44002)	0
Middle SDI	68.3 (58.42 to 79.85)	127.13 (109.86 to 147.28)	2.01965% (1.97379 to 2.06553)	0	9.13 (7.98 to 10.34)	13.5 (11.78 to 15.35)	1.31332% (1.27948 to 1.34718)	0	4.13 (3.61 to 4.81)	3.56 (2.96 to 4.08)	−0.56503% (−0.60947 to −0.52057)	0	72.59 (63.95 to 81.66)	70.4 (61.23 to 79.7)	−0.19294% (−0.23057 to −0.15529)	0
North Africa and Middle East	54.46 (46.72 to 63.02)	80.05 (69.38 to 93.06)	1.28028% (1.18763 to 1.37303)	0	7.94 (7.21 to 8.84)	10.33 (9.3 to 11.47)	0.88846% (0.8342 to 0.94275)	0	4.69 (3.91 to 5.96)	4.13 (3.48 to 4.66)	−0.14478% (−0.29877 to 0.00946)	0.0757	77.7 (66.58 to 93.77)	71.35 (61.06 to 79.15)	−0.11411% (−0.22117 to −0.00693)	0.04553
Oceania	68.44 (56.14 to 82.1)	73.33 (59.5 to 86.91)	0.46861% (0.38018 to 0.55712)	0	8.91 (7.94 to 9.95)	9.23 (8.24 to 10.37)	0.30687% (0.24874 to 0.36504)	0	4.37 (3.38 to 5.59)	3.92 (3.07 to 4.97)	−0.36421% (−0.39491 to −0.3335)	0	79.54 (62.58 to 99.69)	73.38 (57.92 to 92.81)	−0.24292% (−0.27538 to −0.21044)	0
South Asia	63.01 (53.26 to 75.48)	87.55 (73.81 to 103.15)	1.38377% (1.29376 to 1.47387)	0	8.82 (7.62 to 10.11)	10.33 (8.92 to 11.7)	0.73532% (0.66335 to 0.80735)	0	3.53 (2.45 to 4.71)	3.83 (2.93 to 4.74)	0.30461% (0.11777 to 0.4918)	0.00326	62.78 (47.39 to 79.84)	69.65 (55.62 to 83.27)	0.38684% (0.25594 to 0.5179)	0
Southeast Asia	57.52 (49.51 to 66.75)	72.45 (62.98 to 83.82)	0.93014% (0.86092 to 0.99941)	0	8.11 (7.36 to 8.89)	9.62 (8.71 to 10.64)	0.6681% (0.62232 to 0.71391)	0	3.96 (3.2 to 5.05)	4.29 (3.62 to 5.32)	0.1936% (0.09246 to 0.29484)	0.00075	67.74 (56.98 to 82.96)	72.8 (63.15 to 88.22)	0.18032% (0.09472 to 0.26598)	0.00027
Southern Latin America	74.65 (65.02 to 84.46)	86.55 (74.92 to 100.37)	0.57641% (0.44938 to 0.70361)	0	9.73 (8.85 to 10.73)	10.72 (9.75 to 12.22)	0.36442% (0.27336 to 0.45557)	0	3.48 (3.17 to 3.65)	3.06 (2.68 to 3.29)	−0.18818% (−0.28185 to −0.09442)	0.00046	61.05 (56.34 to 65.29)	55.47 (50.08 to 60.39)	−0.1369% (−0.20031 to −0.07344)	2e-04
Southern Sub-Saharan Africa	45 (38.45 to 53.07)	58.38 (49.17 to 68.81)	0.82548% (0.75122 to 0.8998)	0	6.55 (5.71 to 7.43)	8.28 (7.23 to 9.4)	0.78024% (0.73525 to 0.82526)	0	2.77 (2.19 to 3.73)	3.72 (3.16 to 4.1)	1.15673% (0.88274 to 1.43146)	0	44.92 (37.15 to 58.31)	60.01 (51.64 to 66.14)	1.12983% (0.89886 to 1.36133)	0
Tropical Latin America	55.54 (46.84 to 65.81)	79.92 (67.75 to 93.3)	0.71092% (0.57012 to 0.8519)	0	7.68 (6.53 to 8.85)	9.49 (8.2 to 10.75)	0.35172% (0.25197 to 0.45158)	0	3.39 (2.96 to 3.6)	3.45 (2.9 to 3.77)	0.28178% (0.21122 to 0.35239)	0	55.98 (50.95 to 59.71)	60.23 (53.15 to 65.85)	0.30051% (0.24821 to 0.35283)	0
Western Europe	103.44 (94.21 to 113)	129.69 (116.83 to 141.39)	0.54406% (0.42052 to 0.66774)	0	11.49 (10.71 to 12.32)	14.2 (13.03 to 15.32)	0.54017% (0.44584 to 0.63459)	0	3.23 (2.85 to 3.41)	3.15 (2.59 to 3.46)	0.2072% (0.12504 to 0.28943)	3e-05	60.56 (54.95 to 65.58)	61.93 (53.96 to 68.42)	0.22713% (0.16289 to 0.29141)	0
Western Sub-Saharan Africa	51.48 (43.48 to 59.42)	65.22 (55.42 to 74.59)	0.71996% (0.61838 to 0.82164)	0	8.69 (7.75 to 9.69)	10.57 (9.55 to 11.7)	0.64317% (0.58711 to 0.69926)	0	4.33 (3.58 to 5.16)	5.04 (4.06 to 5.77)	0.67215% (0.5987 to 0.74566)	0	67.77 (56.85 to 79.03)	77.92 (64 to 88.69)	0.5974% (0.53613 to 0.65871)	0

### 3.3 Global disease burden trends of PD by sex and age in 2021

**Figure 2:**
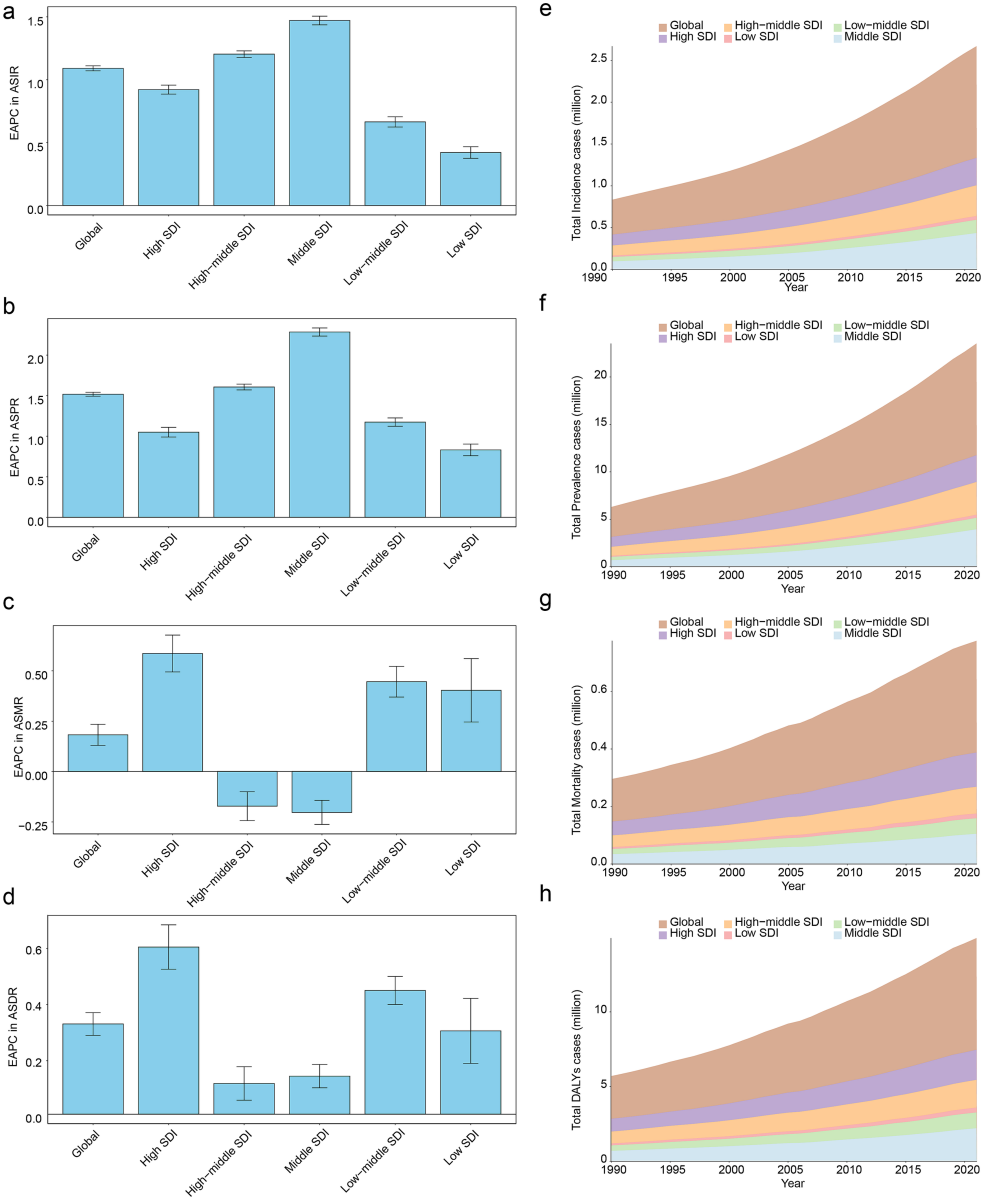
Comparative analysis of PD burden by sex across 21 GBD regions in 2021. Incidence of PD by sex across GBD regions **(a)**, ASIR of PD by sex across GBD regions **(b)**, prevalence of PD by sex across GBD regions **(c)**, ASPR of PD by sex across GBD regions **(d)**, mortality rate of PD by sex across GBD regions **(e)**, ASMR of PD by sex and region **(f)**, numbers of PD-related DALYs by sex and region **(g)**, ASDR of PD by sex and region **(h)**. GBD, Global Burden of Disease.

There were significant differences in incidence, prevalence, mortality, and DALYs, as well as ASIR, ASPR, ASMR, and ASDR of PD between different sexes and 21 GBD regions. In detail, in 2021, region with the highest incidence and ASIR of PD was East Asia, and Among males, both the incidence and ASIR were higher in comparison to females (Figures 2a,b; Supplementary Tables S9, S10). Surprisingly, same results were observed for prevalence and ASPR of PD (Figures 2c,d; Supplementary Tables S11, S12). The area recording the highest mortality rate was East Asia. Among males, High-income North America boasted the highest ASMR, with Southern Latin America coming right after it. In the case of females, Western Sub-Saharan Africa held the top position in terms of ASMR, and Southeast Asia trailed closely behind (Figures 2e,f; Supplementary Tables S13, S14). In addition, East Asia continued to be the region with the greatest number of DALYs. By contrast, for males, region with the uppermost ASDR was East Asia, with High-income North America ranking second. Meanwhile, the High-income Asia Pacific area had the lowest ASDR for males. For females, regions with relatively high ASDR included East Asia and Western Sub - Saharan Africa. Remarkably, in all regions, ASDR of males was higher than that of females (Figures 2g,h; Supplementary Tables S15, S16). This suggested that in processes of research, diagnosis, treatment, and health management of PD, we needed to fully consider sex differences and develop more targeted strategies to address the health challenges posed by this disease to different sex groups.

### 3.4 Impact of SDI on the burden of PD

**Figure 3:**
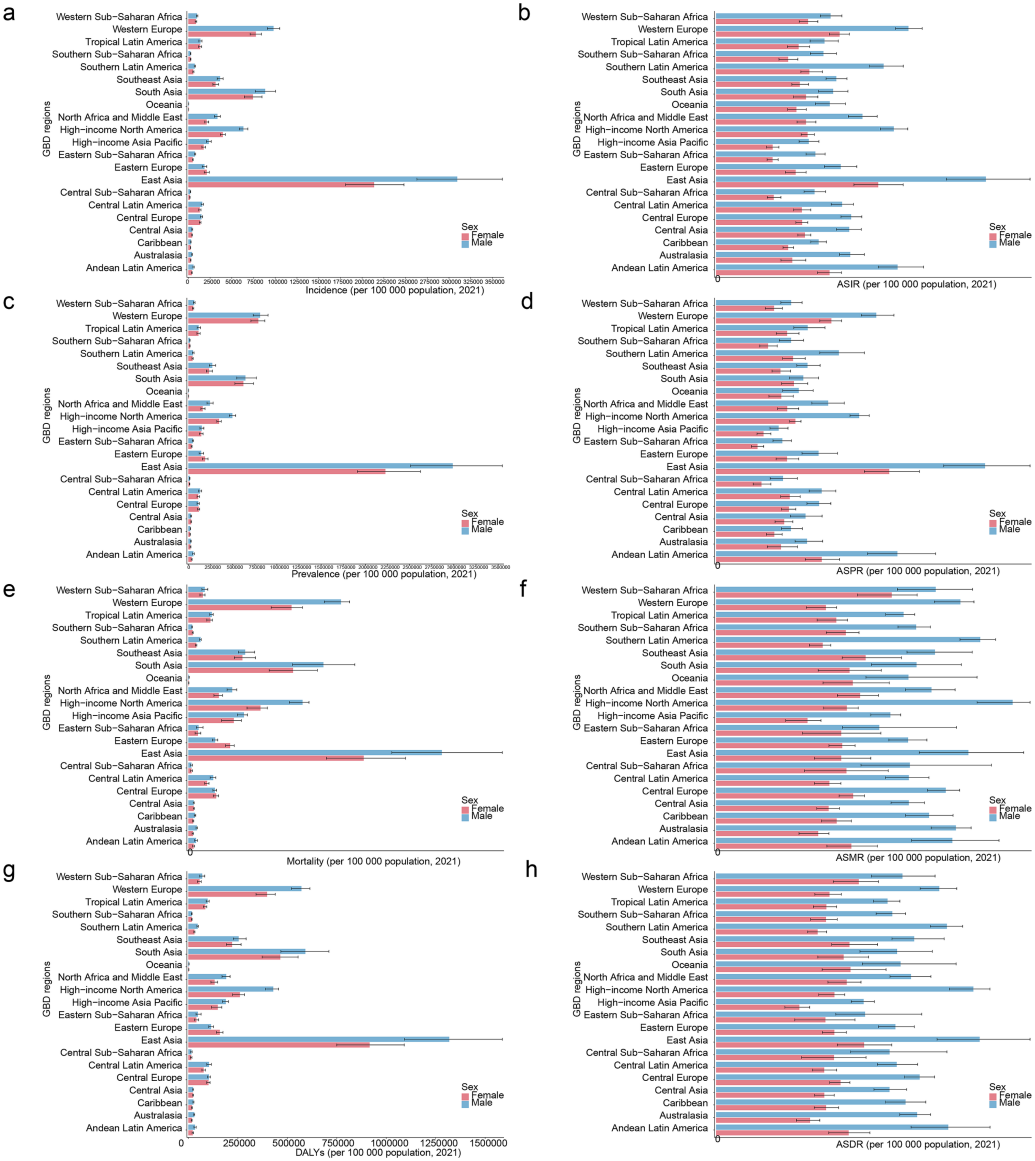
Impact of SDI on the burden of PD from 1990 to 2021. EAPC in ASIR of PD across SDI classification regions **(a)**, EAPC in ASPR of PD across SDI classification regions **(b)**, EAPC in ASMR of PD across SDI classification regions **(c)**, EAPC in ASDR of PD across SDI classification regions **(d)**, temporal trends in total incidence cases of PD globally and across five SDI regions **(e)**, temporal trends in total prevalence cases of PD globally and across five SDI regions **(f)**, temporal trends in total mortality cases of PD globally and across five SDI regions **(g)**, temporal trends in total DALYs cases of PD globally and across five SDI regions **(h)**. SDI, socio-demographic index.

Analyses based on SDI classification regions in 1990–2021 revealed that among the global level and five SDI regions, Middle SDI region exhibited the greatest values of the EAPC in terms of ASIR and ASPR. Conversely, Low SDI region registered the lowest EAPC values for ASIR and ASPR (Figures 3a,b). Among all regions, High SDI region had the top-ranking EAPC value in ASMR (Figure 3c). In addition, in terms of EAPC values in ASDR, order from highest to lowest was High SDI region, Low - middle SDI region, global level, Low SDI region, Middle SDI region, and Middle - high SDI region (Figure 3d). Moreover, stacked area chart showed that as the years increased, the numbers of total incidence cases, total prevalence cases, total mortality cases, and total DALYs cases of PD presented an upward inclination both globally and in five SDI regions (Figures 3e–h), which indicated that PD had posed serious challenges to public health globally and in various regions, thus requiring more medical resources to be invested in long-term management of disease. Meanwhile, it was also necessary to strengthen control of environmental factors and advocate a healthy lifestyle to alleviate burden of PD on individuals, families, and society.

**Figure 4:**
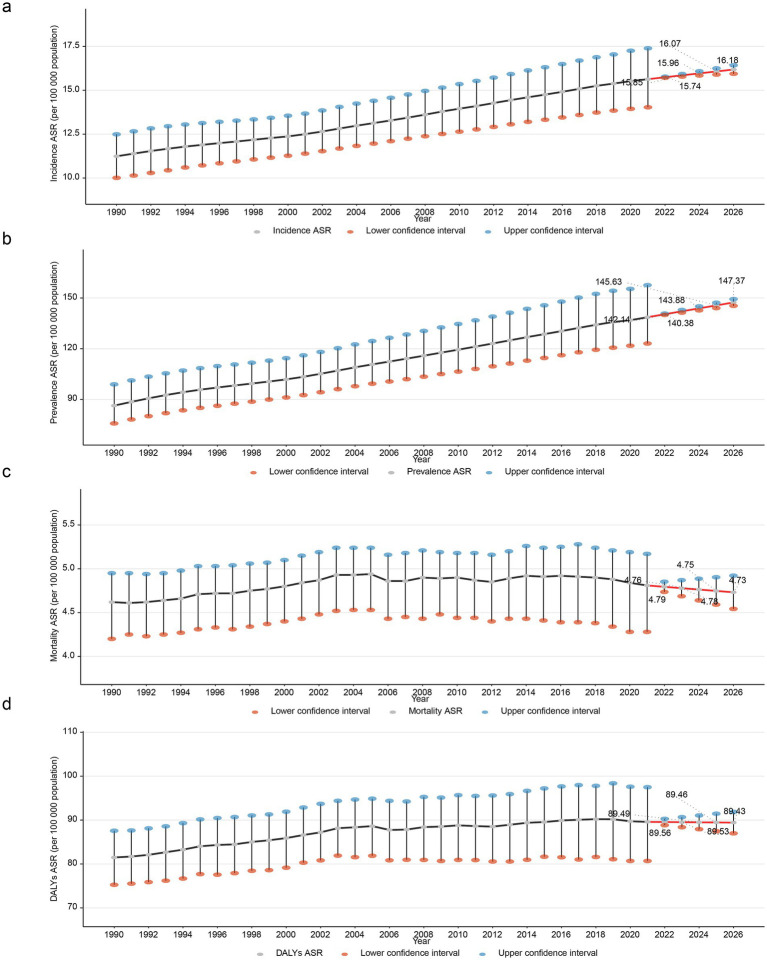
Correlation analysis between PD burden indicators and SDI across 21 GBD regions, 1990–2021. Positive correlation between SDI and ASIR of PD **(a)**, positive association between SDI and ASPR of PD **(b)**, non-significant correlation between SDI and ASMR of PD **(c)**, Non-significant correlation between SDI and ASDR of PD **(d)**.

Subsequently, from 1990 to 2021, correlation analysis between ASIR, ASPR, ASMR, and ASDR of PD and SDI at the level of 21 GBD regions revealed that positive correlation of substance was observed relationship between SDI and ASIR (cor = 0.40, *p* < 0.001) (Figure 4a). Besides, a remarkable positive association was found to exist between SDI and ASPR (cor = 0.44, *p* < 0.001) (Figure 4b). Which indicated that the more developed a region socio-economically, the worse PD’s incidence and prevalence. Frustratingly, there was no significant correlation between changes in ASMR (cor = −0.04, *p* ≥ 0.05) and ASDR (cor = 0.04, *p* ≥ 0.05) among 21 GBD regions and at different SDI levels (Figures 4c,d).

### 3.5 Global disease burden of PD by age and sex

**Figure 5:**
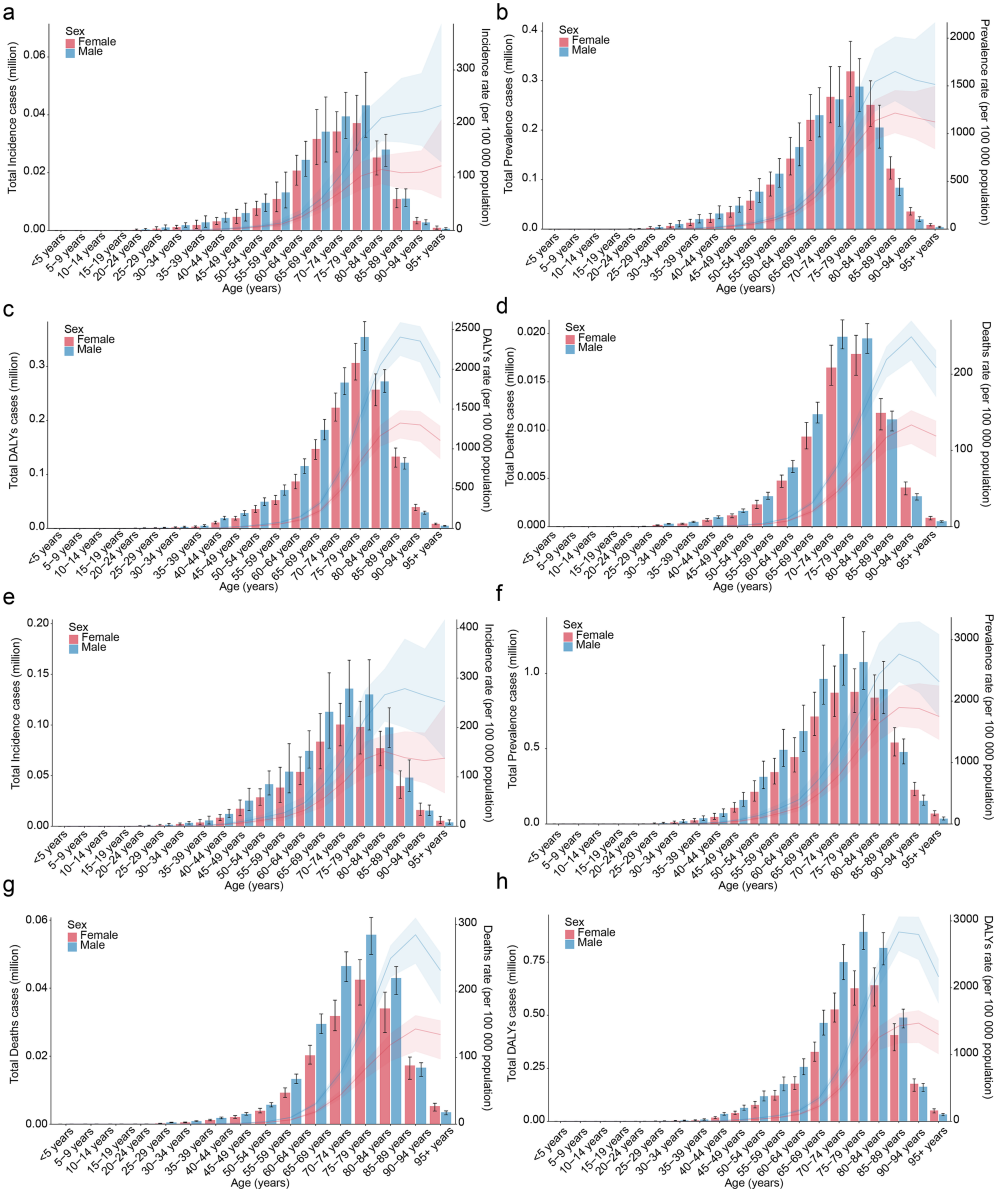
Global disease burden of PD by age and sex. Global age-specific incidence rate of PD by sex in 1990 **(a)**, global age-specific prevalence rate of PD by sex in 1990 **(b)**, global age-specific DALYs rate of PD by sex in 1990 **(c)**, global age-specific mortality rate of PD by sex in 1990 **(d)**, Global age-specific incidence rate of PD by sex in 2021 **(e)**, global age-specific prevalence rate of PD by sex in 2021 **(f)**, global age-specific DALYs rate of PD by sex in 2021 **(g)**, global age-specific mortality rate of PD by sex in 2021 **(h)**.

From 1990 to 2021, as age went up, incidence, prevalence, mortality, and DALYs of PD initially rose and subsequently fell. Precisely, globally, in 1990, the age bracket of 75–79 years had the peak values for incidence, prevalence, and DALYs. Remarkably, except for prevalence, values for the male population were greater than those for the female population (Figures 5a–c). Regarding mortality, the age group of 70–89 had a relatively high mortality. Notably, in the age group of 40–84, males exhibited a higher mortality of PD compared to females; while in the age group of 85–90+, females were higher than males (Figure 5d). In addition, in 2021, incidence of PD was relatively high in individuals aged 65–84 (Figure 5e). For the age cohort above 85, females had a higher prevalence than males (Figure 5f). Mortality rate of PD reached its maximum within the 80–84 age bracket, with males exhibiting a higher mortality than females (Figure 5g). Regarding DALYs, peak was witnessed in the 75–79 age cohort for males, while it peaked in individuals in the 80–84 age range for females (Figure 5h). Overall, in most age groups, compared with female cases, male cases were more numerous, this might indicate that estrogen could have a neuroprotective effect. And the age of PD patients was generally higher, which implied that further in-depth research on age-related pathogenesis would lead to the advancement of more efficient treatment methods and intervention measures for elderly patients with PD.

### 3.6 Prediction of the global PD trend by 2026

**Figure 6:**
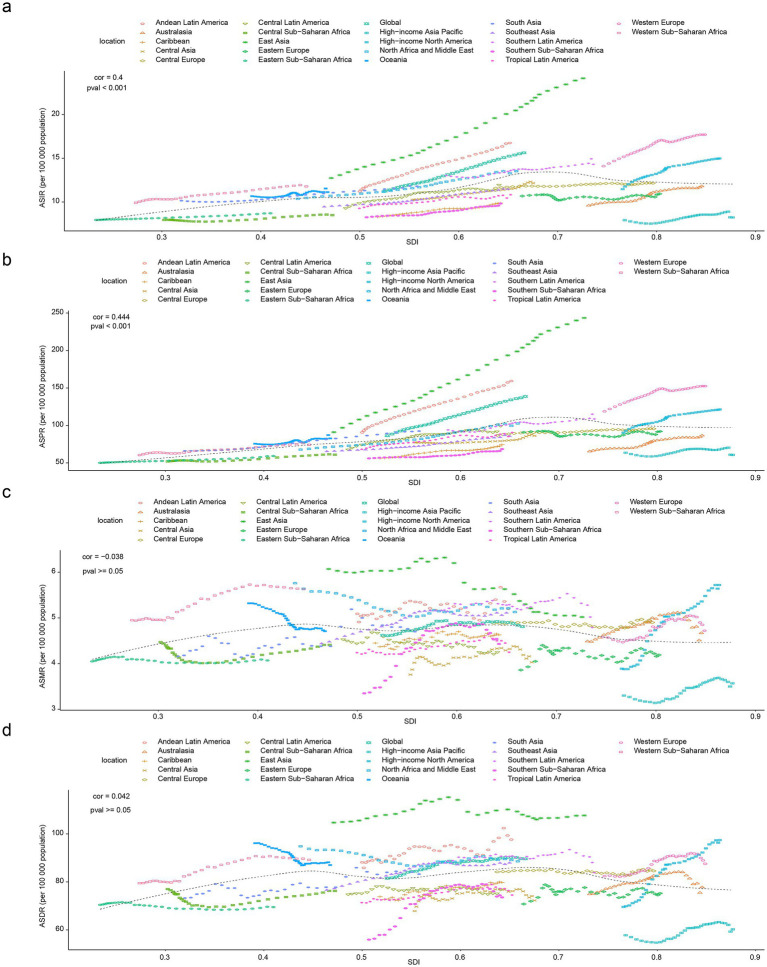
Projected trends in PD burden indicators using ARIMA model from 2022 to 2026. Projected ASIR of PD **(a)**, projected ASPR of PD **(b)**, projected ASMR of PD **(c)**, projected ASDR of PD **(d)**.

**Figure 7:**
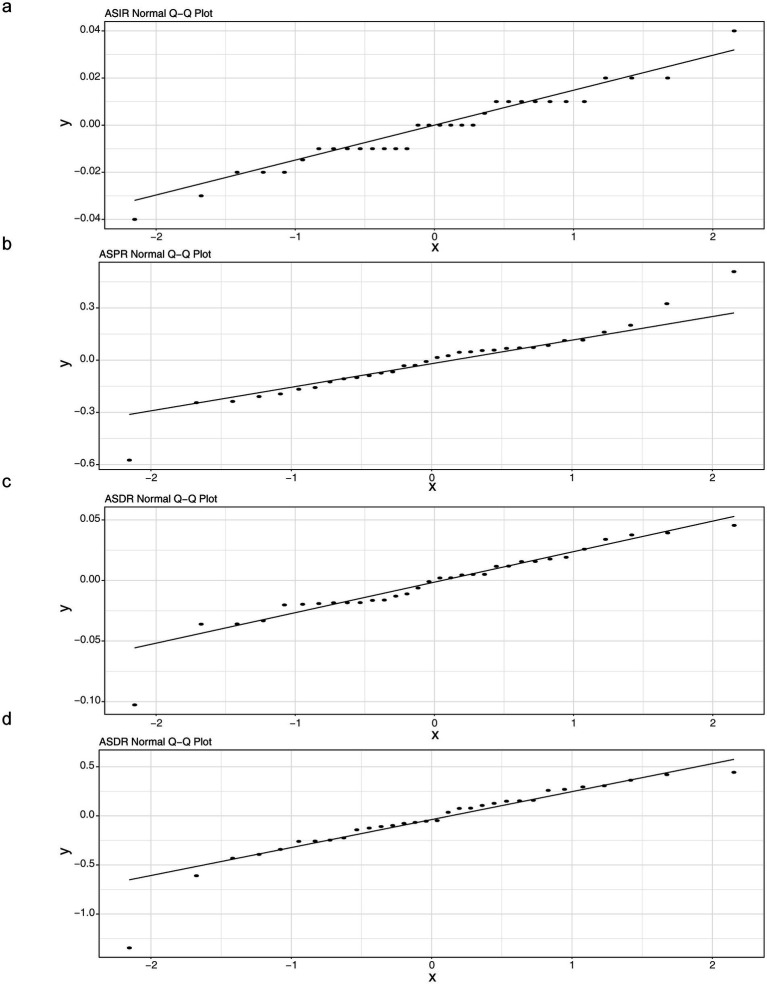
Q-Q plot of residuals normality for the ARIMA model. Q-Q plot of age standardized incidence rate (ASIR) **(a)**, Q-Q plot of age standardized prevalence rate (ASPR) **(b)**, ASPR: Q-Q plot of age standardized disability adjusted life years rate (ASDR) **(c)**, Q-Q plot of age standardized death (mortality) rate (ASDR(ASMR)) **(d)**.

The ARIMA model was used to quantify the projected trends in ASIR, ASPR, ASMR, and ASDR of PD over the following five-year period. As the years go by, ASIR and ASPR of PD manifested an upward trajectory. While ASMR and ASDR of PD exhibited a downward trajectory. Specifically, ASIR was projected to increase from 15.74 per 100,000 population in 2022 to 16.18 per 100,000 population in 2026 (Figure 6a). The ASPR was projected to increase from 140.38 per 100,000 population in 2022 to 147.37 per 100,000 population in 2026 (Figure 6b). Which manifested that PD would present a substantial challenge to global health. On the contrary, ASMR would slightly decline from 4.79 per 100,000 in 2022 to 4.73 per 100,000 in 2026 (Figure 6c). Besides, ASDR would slightly decline from 89.56 per 100,000 in 2022 to 89.43 per 100,000 in 2026 (Figure 6d). The AIC and BIC values of both ASIR and ASPR prediction models were negative, indicating that these models had good fitting effects and moderate complexity (Table 3). Meanwhile, the RMSE, MAE, MPE, MAPE, MASE, ACF1, ME, and MPE values of ASIR, ASPR, and ASMR models were all small, suggesting that the predicted values of the models were close to the actual values, with high precision and performance (Table 4). Additionally, the points in the ASIR, ASPR, ASDR, and ASMR data basically fell on the line, indicating that the residuals followed a normal distribution (Figures 7a–d). The Ljung-Box test showed that the autocorrelation coefficients of the residuals were zero, indicating that the residuals were white noise (without autocorrelation) (Table 5). The performance of the models was evaluated by comprehensively using AIC/BIC, error indicators, and residual tests. It was found that the prediction performance of the models was good, providing a reference for similar studies and helping to improve the standardization and rigor of model construction.

**Table 3:** The AIC and BIC of the ARIMA model.

Metric	AIC^1^	BIC^2^
ASIR	−159.7490216	−158.3478242
ASPR	−8.147747606	−3.845785992
ASDR	28.61721412	−119.068226
ASMR	28.61721412	31.41960888

^1^AIC: akaike information criterion.

^2^BIC: bayes information criterion.

**Table 4:** Different indicators for the predictive performance of the ARIMA model.

Metric	ME^1^	RMSE^2^	MAE^3^	MPE^4^	MAPE^5^	MASE^6^	ACF1^7^	Model
ASIR	−0.001553685	0.016049407	0.012492852	−0.011061963	0.09351808	0.088218319	0.034906259	ARIMA
ASPR	−0.014106796	0.18764266	0.136895159	−0.014077033	0.123483683	0.081064946	0.081220638	ARIMA
ASDR	−0.04958441	0.348738062	0.252192159	−0.05477294	0.288578987	0.668773048	0.162331886	ARIMA
ASMR	−0.002906215	0.028315211	0.021200785	−0.058198933	0.437966741	0.900307299	0.115657691	ARIMA

^1^ME: Mean Error.

^2^RMSE: Root Mean Squared Error.

^3^MAE: Mean Absolute Error.

^4^MPE: Mean Percentage Error.

^5^MAPE: Mean Absolute Percentage Error.

^6^MASE: Mean Absolute Scaled Error.

^7^ACF1: Autocorrelation Function at Lag 1.

**Table 5:** The results of the Ljung-Box test for autocorrelation of model residuals.

Metric	Test	Statistic	*p*_value
ASIR	Box-Ljung test	0.042763556	0.836171125
ASPR	Box-Ljung test	0.231526123	0.630394533
ASDR	Box-Ljung test	0.924857602	0.336202749
ASMR	Box-Ljung test	0.469479075	0.493226916

## 4 Discussion

PD is a prevalent neurodegenerative disease that can cause considerable disability and is linked to motor, non-motor, and cognitive symptoms (Morris et al., 2024). With the increasing global aging population, PD had become a growing global public health burden (Morris et al., 2024). However, a comprehensive assessment of the temporal and geographical trends in the global burden of PD remains inadequate, lacking recent GBD studies. This study found that, compared with 1990, the ASIR, ASPR, ASMR, and ASDR of global PD in 2021 showed an upward trend. The study results indicated that in 2021, the age group of 65 to 84 years exhibited a relatively higher incidence of PD (Figure 5e). It is recommended to implement questionnaire-based screening for non-motor symptoms (such as sleep disorders, constipation, and anxiety) in primary care settings for individuals aged 65 and older. Furthermore, given the later onset of the disease, raising public awareness and understanding of PD, along with conducting health education initiatives, is critical for early screening and diagnosis. Additionally, rehabilitation therapist availability and care bed capacity should be proactively planned based on regional incidence rates to provide patients with access to physical therapy, speech rehabilitation, and psychological support.

This study further explored the trends in ASPR, ASIR, ASMR, and ASDR caused by PD in 21 GBD regions, 5 SDI levels, and global regions. The results showed that countries with middle SDI had the highest ASPR and ASIR, while countries with high SDI had the highest ASMR and ASDR. Meanwhile, from 1990 to 2021, the number of PD cases, incidents, deaths, and DALYs all showed an upward trend with increasing years. By analyzing and predicting the ASPR and ASIR of PD from 2022 to 2026, this study showed an upward trend, while the ASMR and ASDR may show a downward trend. This indicates a heavy burden of PD and provides an important reference for predicting and preventing the future burden of PD. By employing the ARIMA model to model and predict the four key indicators of PD (ASIR, ASPR, ASMR, ASDR), combined with model evaluation metrics such as AIC and BIC, as well as residual analysis, the fitting characteristics and predictive reliability of different indicators were revealed. The AIC and BIC values of both the ASIR and ASPR models were negative, indicating that the models exhibited good fitting performance with moderate complexity (Claris and Peter, 2023). The residuals of all four indicators approximately followed a normal distribution, and the Ljung-Box test confirmed that the residuals were white noise (no autocorrelation), suggesting that the core assumptions of the ARIMA model (data stationarity, linear dependence) hold true in most scenarios (Wang et al., 2023).

From 1990 to 2021, the global trend of ASIR, ASPR, and ASDR for PD showed an overall increase, which was consistent with previous studies (Ou et al., 2021). With the acceleration of the global aging process, the proportion of the elderly population will continue to increase, and the total global population will continue to grow. Even if the incidence rate of PD remains relatively stable, due to the larger base, the number of PD cases, prevalent cases, and the resulting disability-adjusted life years will increase accordingly, leading to an overall upward trend in ASIR, ASPR, and ASDR. As medical technology continues to advance, PD diagnostic methods and techniques have become more comprehensive and accurate. Some PD cases that were difficult to diagnose or missed in the past can now be accurately diagnosed, increasing indicators such as incidence and prevalence rates in statistical data (Alcalay et al., 2020; Rizzo et al., 2016). Furthermore, improvements in medical conditions and advancements in medicine have extended the overall lifespan of humans, allowing PD patients to spend more time in a diseased state. This increases the cumulative prevalence of the disease and disability-adjusted life-years, thereby increasing ASPR and ASDR.

Our results indicate an increase in ASMR for males and a decrease in ASMR for females (Table 1), with a higher PD burden observed in most males compared to females (Figure 2). This may be attributed to the potential neuroprotective effects of estrogen in females, which can promote the survival and repair of nerve cells, enhance antioxidant capacity, and reduce nerve cell damage and death, thereby lowering the severity and mortality of PD to some extent (Means et al., 2021). Males, on the other hand, lack this estrogen-based protection. Additionally, higher occupational risk factors and unhealthy lifestyle habits (such as alcohol consumption and staying up late) can adversely affect nerve cells (causing inflammation and oxidative stress) (Dubey et al., 2023; Korf, 2024). Consequently, as age increases, nerve cells become more susceptible to damage, leading to a relatively faster progression of PD and increased mortality rates. Figure 2 demonstrates that the age group with higher ASIR, ASPR, ASMR, and ASDR was 75–79.

PD was a neurodegenerative disease that primarily affects middle-aged and older adults, and age was one of the major risk factors for PD (Braccia et al., 2025). As individuals age, brain neurons undergo natural aging processes that increase the risk of degenerative changes and increase the ASIR, ASPR, ASMR, and ASDR of PD (Braccia et al., 2025). The results also suggest that SDI had some effect on PD (Figures 3, 4). Across the 21 GBD regions, ASIR, ASPR, and SDI levels were significantly positively correlated. This suggests that regions with higher economic levels tend to have relatively higher incidence and prevalence rates of PD. Regions with higher SDI have better economic development and more healthcare investment. This leads to more funds for medical institutions, equipment, and talent, enabling more accurate PD diagnosis and higher ASIR and ASPR statistics. Additionally, regions with a high SDI have more comprehensive healthcare systems and robust disease diagnosis and surveillance systems, leading to more accurate and comprehensive reporting and statistics for diseases such as PD (Ou et al., 2021; Liu and Yu, 2025). This implies that more cases can be included in the statistics, making ASIR and ASPR data more reflective of the true incidence and prevalence of the disease. Furthermore, as SDI increases, people’s lifestyles gradually change, such as reduced physical activity and increased chronic stress. Physical inactivity can lead to reduced nervous system function, while chronic stress can affect neurotransmitter secretion and regulation, increasing the risk of PD and subsequently increasing ASIR and ASPR (Johansson et al., 2023; Goonetilleke et al., 2013). Therefore, differentiated strategies should be implemented for regions with varying levels of SDI. For instance, high-SDI countries can leverage specialized alliances to promote gene therapy and healthcare coverage, while middle- and low-SDI countries can collaborate with the WHO to disseminate primary screening and low-cost medications (de Souza et al., 2016). Regional resource allocation should be tailored accordingly. High-SDI countries should optimize healthcare resource allocation and develop home-based smart monitoring systems (Zhao et al., 2022), strengthen prevention and early intervention, and implement community-based programs such as olfactory testing and non-motor symptom screening for early identification. Big data can be utilized to identify high-risk populations, while exercise therapy and cognitive training tailored to elderly individuals should be promoted (Gupta et al., 2023). Middle-SDI countries should enhance primary screening and environmental toxin monitoring, whereas low-SDI countries should improve data quality and basic drug supply. In East Asia, efforts should focus on bridging the urban–rural healthcare gap (Sullivan et al., 2023). Population interventions should be tailored by gender and age. Males should be prioritized for occupational exposure prevention (Eng et al., 2011), females should explore estrogen-related preventive measures, middle-aged individuals should undergo brain health screening (Santos et al., 2023), and elderly individuals should benefit from community-friendly environments (Horgan et al., 2024). In terms of policy advocacy, research evidence should be incorporated into prevention and treatment guidelines, data visualization tools should be employed to aid decision-making, and funding should be allocated to cross-regional cohort studies and predictive system development (Prasinos et al., 2022). Overall, PD prevention and control should shift toward multidimensional “environment-society-biology” interventions. Differentiated strategies and targeted measures should be adopted to curb the increasing burden and achieve healthy aging goals.

To validate the reliability of our findings, we compared them with data from previous years. The Global Report on Aging and Health (2015) highlighted a gradual increase in the incidence and prevalence of PD, with its burden driven primarily by long-term disability rather than mortality (Beard et al., 2016), which aligns with our results. A 2024 systematic review and meta-analysis published in The Lancet Healthy Longevity reported that the EAPC in PD prevalence was significantly higher during 2004–2023 compared to 1980–2003, with higher prevalence rates in high-SDI countries and a global male predominance in PD prevalence—conclusions consistent with our findings (Zhu et al., 2024). These comparisons suggest the credibility of our results. However, discrepancies remain in aspects such as prevalence variations across countries, which may stem from methodological differences: GBD relies on vital registration systems, literature meta-analyses, and modeling, while studies by organizations like WHO often use prospective cohorts or hospital-based registries, potentially yielding results closer to clinical observations in high-SDI regions. As noted, our findings provide a foundation for governments to tailor public health strategies for PD patients across different SDI regions and age groups. Moving forward, improvements in data granularity (e.g., subpopulation-level analyses) and technological advancements (e.g., AI-driven predictive models) will further empower GBD to enhance precision in PD prevention and control, advancing the global goal to reduce health inequities.

While our model assumes that trends in disease burden across regions are primarily influenced by age, sex, and the SDI, it does not account for other factors such as comorbidities, environmental influences, or subnational-level heterogeneity (e.g., within large countries like China and India), which may lead to deviations in burden predictions in groups with high coexisting diseases. Additionally, external shocks (e.g., pandemics or policy shifts) may impact PD burden but were not incorporated into the current framework. Furthermore, data from some countries (e.g., South Sudan, where conflict has limited data availability) were excluded from our analysis, and future studies could expand the scope as data improve. Nevertheless, our model offers a comprehensive global perspective that helps identify regions and populations with the heaviest PD burden. In future iterations, we aim to integrate additional variables to enhance predictive accuracy.

## 5 Conclusion

This study systematically analyzes the burden of Parkinson’s disease (PD) globally and across different regions, age groups, genders, and Socioeconomic Development Index (SDI) levels, based on the latest GBD2021 database covering a broad geographical and temporal range. The findings reveal that from 1990 to 2021, the age-standardized incidence, prevalence, and burden rates of PD among both males and females worldwide exhibited an upward trend. While age-standardized mortality rates increased among males and decreased among females, the East Asian region bore the heaviest PD burden, with significantly higher rates among males, correlating with aging, diagnostic rates, and environmental exposures. High-SDI countries demonstrated elevated mortality and burden rates due to profound aging, whereas medium-SDI regions experienced the fastest growth in incidence and prevalence rates due to industrialization and lifestyle shifts. The 75–79 age group consistently represented the core burden cohort. Projections for 2022–2026 indicate sustained increases in incidence and prevalence rates, though mortality and burden rates may slightly decline due to medical advancements, the overall burden remains exacerbated. The study underscores the need for tiered prevention and control strategies globally: high-SDI countries should advance precision diagnostics and interdisciplinary care, medium-and low-SDI regions should strengthen primary screening and environmental governance, and targeted occupational protection and healthy aging interventions should be implemented for males and elderly populations. It calls for international collaboration and technological innovation to address the public health challenges posed by PD.

## Data Availability

The datasets presented in this study can be found in online repositories. The names of the repository/repositories and accession number(s) can be found in the article/Supplementary material.

## Footnotes

^1^ https://ghdx.healthdata.org/ihme_data

^2^ https://vizhub.healthdata.org/gbd-results/

^3^ https://ghdx.healthdata.org/gbd-2021/sources

^4^ https://davidgohel.github.io/flextable/

^5^ https://pkg.robjhyndman.com/forecast/

^6^ https://CRAN.R-project.org/package=tseries

## Glossary

PD: Parkinson’s Disease
GBD: Global Burden of Disease
ASIR: Age Standardized Incidence Rate
ASPR: Age Standardized Prevalence Rate
ASMR: Age Standardized Mortality Rate
DALYs: Disability Adjusted Life Years
ASDR: Age Standardized Disability Adjusted Life Years Rate
NMDA: N-Methyl-D-Aspartate
YLDs: Years Lived With Disability Years
ASR: Age-Standardized Rate
EAPC: Estimated Annual Percentage Change
SDI: Socio-Demographic Index
ICD: International Classification of Diseases
UI: Uncertainty Interval
CI: Confidence Interval
ARIMA: Autoregressive Integrated Moving Average
AIC: Akaike Information Criterion
BIC: Bayes Information Criterion
ME: Mean Error
RMSE: Root Mean Squared Error
MAE: Mean Absolute Error
MPE: Mean Percentage Error
MAPE: Mean Absolute Percentage Error
MASE: Mean Absolute Scaled Error
ACF1: Autocorrelation Function at Lag 1

## References

[ref1] Alcalay R. N. Kehoe C. Shorr E. Battista R. Hall A. Simuni T.. (2020). Genetic testing for Parkinson disease: current practice, knowledge, and attitudes among US and Canadian movement disorders specialists. Genet. Med. 22, 574–580. doi: 10.1038/s41436-019-0684-x, PMID: 31680121 PMC7056638

[ref2] Al-Kuraishy H. M. Al-Gareeb A. Alexiou A. Papadakis M. Alsayegh A. P.. (2023). Pros and cons for statins use and risk of Parkinson’s disease: an updated perspective. Pharmacol. Res. Perspect. 11:e01063. doi: 10.1002/prp2.1063, PMID: 36811160 PMC9944858

[ref3] Beard J. R. Officer A. M. Cassels A. K. (2016). The world report on ageing and health. Gerontologist 56, S163–S166. doi: 10.1093/geront/gnw037, PMID: 26994257

[ref4] Braccia A. Golfrè Andreasi N. Ghielmetti F. Aquino D. Savoldi A. P. Cilia R.. (2025). Magnetic resonance-guided focused ultrasound Thalamotomy in a prospective cohort of 52 patients with Parkinson’s disease: a possible critical role of age and lesion volume for predicting tremor relapse. Mov. Disord. 40, 478–489. doi: 10.1002/mds.30093, PMID: 39825750 PMC11926496

[ref5] Cen J. Wang Q. Cheng L. Gao Q. Wang H. Sun F. (2024). Global, regional, and national burden and trends of migraine among women of childbearing age from 1990 to 2021: insights from the global burden of disease study 2021. J. Headache Pain 25:96. doi: 10.1186/s10194-024-01798-z, PMID: 38844846 PMC11157953

[ref6] Claris S. Peter N. (2023). Arima model in predicting of Covid-19 epidemic for the southern Africa region. Afr J Infect Dis 17, 1–9. doi: 10.21010/Ajidv17i1.1, PMID: 36756487 PMC9885024

[ref7] de Souza J. A. Hunt B. Asirwa F. C. Adebamowo C. Lopes G. (2016). Global Health equity: Cancer care outcome disparities in high-, middle-, and low-income countries. J. Clin. Oncol. 34, 6–13. doi: 10.1200/JCO.2015.62.2860, PMID: 26578608 PMC5795715

[ref8] Dubey A. Kumar S. Acharya S. Nagendra V. Khurana K. (2023). Acquired Parkinson’s disease in alcoholic cirrhosis: the rarest association. Cureus 15:e34968. doi: 10.7759/cureus.34968 36938289 PMC10019375

[ref9] Eng A. t Mannetje A. McLean D. Ellison-Loschmann L. Cheng S. Pearce N. (2011). Gender differences in occupational exposure patterns. Occup. Environ. Med. 68, 888–894. doi: 10.1136/oem.2010.064097, PMID: 21486991

[ref10] Fang R. Yu Y. F. Li E. J. Lv N. X. Liu Z. C. Zhou H. G.. (2022). Global, regional, national burden and gender disparity of cataract: findings from the global burden of disease study 2019. BMC Public Health 22:2068. doi: 10.1186/s12889-022-14491-0, PMID: 36369026 PMC9652134

[ref11] GBD 2016 Parkinson’s Disease Collaborators (2018). Global, regional, and national burden of Parkinson’s disease, 1990-2016: a systematic analysis for the global burden of disease study 2016. Lancet Neurol. 17, 939–953. doi: 10.1016/S1474-4422(18)30295-3 30287051 PMC6191528

[ref12] GBD 2019 Diseases and Injuries Collaborators (2020). Global burden of 369 diseases and injuries in 204 countries and territories, 1990-2019: a systematic analysis for the global burden of disease study 2019. Lancet 396, 1204–1222. doi: 10.1016/S0140-6736(20)30925-9, PMID: 33069326 PMC7567026

[ref13] GBD 2021 Diseases and Injuries Collaborators (2024). Global incidence, prevalence, years lived with disability (YLDs), disability-adjusted life-years (DALYs), and healthy life expectancy (HALE) for 371 diseases and injuries in 204 countries and territories and 811 subnational locations, 1990-2021: a systematic analysis for the global burden of disease study 2021. Lancet 403, 2133–2161. doi: 10.1016/S0140-6736(24)00757-8 38642570 PMC11122111

[ref14] GBD 2021 Tuberculosis Collaborators (2024). Global, regional, and national age-specific progress towards the 2020 milestones of the WHO end TB strategy: a systematic analysis for the global burden of disease study 2021. Lancet Infect. Dis. 24, 698–725. doi: 10.1016/S1473-3099(24)00007-0, PMID: 38518787 PMC11187709

[ref15] Gibb W. R. Lees A. J. (1988). The relevance of the Lewy body to the pathogenesis of idiopathic Parkinson’s disease. J. Neurol. Neurosurg. Psychiatry 51, 745–752. doi: 10.1136/jnnp.51.6.745, PMID: 2841426 PMC1033142

[ref16] Goonetilleke L. Ralevic V. Dunn W. R. (2013). Influence of pressure on adenosine triphosphate function as a sympathetic neurotransmitter in small mesenteric arteries from the spontaneously hypertensive rat. J. Hypertens. 31, 312–320. doi: 10.1097/HJH.0b013e32835bd74d, PMID: 23263239

[ref17] Gupta R. Kumari S. Senapati A. Ambasta R. K. Kumar P. (2023). New era of artificial intelligence and machine learning-based detection, diagnosis, and therapeutics in Parkinson’s disease. Ageing Res. Rev. 90:102013. doi: 10.1016/j.arr.2023.102013, PMID: 37429545

[ref18] Gustavsson E. K. Zhang D. Reynolds R. H. Garcia-Ruiz S. Ryten M. (2022). Ggtranscript: an R package for the visualization and interpretation of transcript isoforms using ggplot2. Bioinformatics 38, 3844–3846. doi: 10.1093/bioinformatics/btac409, PMID: 35751589 PMC9344834

[ref19] He Y. Wang W. Yang T. Rosalind Thomas E. Dai R.. (2022). The potential role of voltage-dependent Anion Channel in the treatment of Parkinson’s disease. Oxidative Med. Cell. Longev. 2022:4665530. doi: 10.1155/2022/4665530 PMC9556184 36246397

[ref20] Hong X. Guo W. Li S. (2022). Lower blood lipid level is associated with the occurrence of Parkinson’s disease: a Meta-analysis and systematic review. Int. J. Clin. Pract. 2022:9773038. 35801143 10.1155/2022/9773038 PMC9203242

[ref21] Horgan S. Prorok J. Ellis K. Mullaly L. Cassidy K. L. Seitz D.. (2024). Optimizing older adult mental health in support of healthy ageing: a pluralistic framework to inform transformative change across community and healthcare domains. Int. J. Environ. Res. Public Health 21. doi: 10.3390/ijerph21060664, PMID: 38928911 PMC11203904

[ref22] Johansson H. Folkerts A. K. Hammarström I. Kalbe E. Leavy B. (2023). Effects of motor-cognitive training on dual-task performance in people with Parkinson’s disease: a systematic review and meta-analysis. J. Neurol. 270, 2890–2907. doi: 10.1007/s00415-023-11610-8, PMID: 36820916 PMC10188503

[ref23] Korf H. W. (2024). Photoneuroendocrine, circadian and seasonal systems: from photoneuroendocrinology to circadian biology and medicine. Cell Tissue Res. 400, 217–240. doi: 10.1155/2022/9773038 39264444 PMC12089256

[ref24] Li Q. Cao J. Liu X. Luo X. Su G. Wang D.. (2022). The diagnostic value of diffusion kurtosis imaging in Parkinson’s disease: a systematic review and meta-analysis. Ann Transl Med 10:474. doi: 10.21037/atm-22-1461, PMID: 35571428 PMC9096385

[ref25] Liu C. Yu J. (2025). Global inequality in the burden of hepatitis B from 1990 to 2021: findings from the global burden of disease study 2021: letter to the editor on “comprehensive approach to controlling chronic hepatitis B in China”. Clin. Mol. Hepatol. 31, e134–e136. doi: 10.3350/cmh.2024.1085, PMID: 39689703 PMC12016633

[ref26] Mangiola S. Doyle M. A. Papenfuss A. T. (2021). Interfacing Seurat with the R tidy universe. Bioinformatics 37, 4100–4107. doi: 10.1093/bioinformatics/btab404, PMID: 34028547 PMC9502154

[ref27] McCormack J. Vandermeer B. Allan G. M. (2013). How confidence intervals become confusion intervals. BMC Med. Res. Methodol. 13:134. doi: 10.1186/1471-2288-13-134, PMID: 24172248 PMC3818447

[ref28] Means J. C. Lopez A. A. Koulen P. (2021). Estrogen protects optic nerve head astrocytes against oxidative stress by preventing Caspase-3 activation, tau Dephosphorylation at Ser(422) and the formation of tau protein aggregates. Cell. Mol. Neurobiol. 41, 449–458. doi: 10.1007/s10571-020-00859-6, PMID: 32385548 PMC7648721

[ref29] Morris H. R. Spillantini M. G. Sue C. M. Williams-Gray C. H. (2024). The pathogenesis of Parkinson’s disease. Lancet 403, 293–304. doi: 10.1016/S0140-6736(23)01478-2, PMID: 38245249

[ref30] Murray C. J. L. (2022). The global burden of disease study at 30 years. Nat. Med. 28, 2019–2026. doi: 10.1038/s41591-022-01990-1, PMID: 36216939

[ref31] Murray C. J. L. GBD 2021 Collaborators (2024). Findings from the global burden of disease study 2021. Lancet 403, 2259–2262. doi: 10.1016/S0140-6736(24)00769-4, PMID: 38762327

[ref32] Ou Z. Pan J. Tang S. Duan D. Yu D. Nong H.. (2021). Global trends in the incidence, prevalence, and years lived with disability of Parkinson’s disease in 204 countries/territories from 1990 to 2019. Front. Public Health 9:776847. doi: 10.3389/fpubh.2021.776847, PMID: 34950630 PMC8688697

[ref33] Prasinos M. Basdekis I. Anisetti M. Spanoudakis G. Koutsouris D. Damiani E. (2022). A modelling framework for evidence-based public health policy making. IEEE J. Biomed. Health Inform. 26, 2388–2399. doi: 10.1109/JBHI.2022.3142503, PMID: 35025752

[ref34] Rizzo G. Copetti M. Arcuti S. Martino D. Fontana A. Logroscino G. (2016). Accuracy of clinical diagnosis of Parkinson disease: a systematic review and meta-analysis. Neurology 86, 566–576. doi: 10.1212/WNL.0000000000002350, PMID: 26764028

[ref35] Santos E. M. C. P. Canhestro A. M. G. D. C. Rosário J. M. O. A. Fonseca C. J. V. Pinho L. M. G. Arco H. M. C. L. R.. (2023). Efficacy of health promotion interventions aimed to improve health gains in middle-aged adults-a Systematic Review. Geriatrics (Basel) 8. doi: 10.3390/geriatrics8030050 PMC10204560 37218830

[ref36] Serva S. N. Bernstein J. Thompson J. A. Kern D. S. Ojemann S. G. (2022). An update on advanced therapies for Parkinson’s disease: from gene therapy to neuromodulation. Front. Surg. 9:863921. doi: 10.3389/fsurg.2022.863921, PMID: 36211256 PMC9537763

[ref37] Stevenson T. (1997). Drug therapy in the management of Parkinson’s disease. Br. J. Nurs. 6, 144–150. doi: 10.12968/bjon.1997.6.3.144, PMID: 9104119

[ref38] Sullivan D. R. Sarma N. Hough C. L. Mularski R. A. Osborne M. L. Dirksen K. M.. (2023). Differences in US regional healthcare allocation guidelines during the COVID-19 pandemic. J. Gen. Intern. Med. 38, 269–272. doi: 10.1007/s11606-022-07861-2, PMID: 36348220 PMC9643918

[ref39] Verhaegen J. C. F. Vorimore C. Galletta C. Rakhra K. Slullitel P. A. Beaule P. E.. (2024). How to best identify acetabular retroversion on radiographs: thresholds to guide clinical practice. Am. J. Sports Med. 52, 2728–2739. doi: 10.1177/03635465241265087, PMID: 39166331

[ref40] Waller S. Williams L. Morales-Briceño H. Fung V. S. C. (2021). The initial diagnosis and management of Parkinson’s disease. Aust. J. Gen. Pract. 50, 793–800. doi: 10.31128/AJGP-07-21-6087, PMID: 34713282

[ref41] Wang M. Jiang Z. You M. Wang T. Ma L. Li X.. (2023). An autoregressive integrated moving average model for predicting varicella outbreaks - China, 2019. China CDC Wkly 5, 698–702. doi: 10.46234/ccdcw2023.134, PMID: 37593138 PMC10427340

[ref42] Xu T. Dong W. Liu J. Yin P. Wang Z. Zhang L.. (2024). Disease burden of Parkinson’s disease in China and its provinces from 1990 to 2021: findings from the global burden of disease study 2021. Lancet Reg Health West Pac 46:101078. doi: 10.1016/j.lanwpc.2024.101078 38745974 PMC11091691

[ref43] Yu R. L. Wu R. M. (2022). Mild cognitive impairment in patients with Parkinson’s disease: an updated mini-review and future outlook. Front. Aging Neurosci. 14:943438. doi: 10.3389/fnagi.2022.943438, PMID: 36147702 PMC9485585

[ref44] Zhao Z. Wang Z. Garcia-Campayo J. Perez H. M. (2022). The dissemination strategy of an urban smart medical tourism image by big data analysis technology. Int. J. Environ. Res. Public Health 19. doi: 10.3390/ijerph192215330, PMID: 36430048 PMC9690489

[ref45] Zhu J. Cui Y. Zhang J. Yan R. Su D. Zhao D.. (2024). Temporal trends in the prevalence of Parkinson’s disease from 1980 to 2023: a systematic review and meta-analysis. Lancet Healthy Longev 5, e464–e479. doi: 10.1016/S2666-7568(24)00094-1, PMID: 38945129

