# Supplementary material for: The burden of Parkinson’s disease, 1990–2021: a systematic analysis of the Global Burden of Disease study 2021
Source: Front Aging Neurosci. 2025 Jun 16;17:1596392. doi: 10.3389/fnagi.2025.1596392 (PMC12206750; doi:10.3389/fnagi.2025.1596392)
Supplement: Supplementary file 1 [file Data_Sheet_1.PDF]

Supplementary materials for: Global, regional, and national burden of Parkinson's disease, 1990-2021: a systematic analysis for the Global Burden of Disease Study 2021

contents

Table S1: ASIR of PD by country in 1990 2

Table S2: ASIR of PD by country in 2021 11

Table S3: ASPR of PD by country in 1990 20

Table S4: ASPR of PD by country in 2021 30

Table S5: ASMR of PD by country in 1990 39

Table S6: ASMR of PD by country in 2021 49

Table S7: ASDR of PD by country in 1990 58

Table S8: ASDR of PD by country in 2021 68

Table S1: ASIR of PD by country in 1990

location	val	upper	lower	Rate_1990
Netherlands	18.78	20.45	16.77	18.78 (16.77 to 20.45)
Israel	18.45	21.73	16.07	18.45 (16.07 to 21.73)
Cyprus	17.52	20.37	15.43	17.52 (15.43 to 20.37)
Italy	17.47	19.99	14.88	17.47 (14.88 to 19.99)
United Arab Emirates	17.34	19.16	15.64	17.34 (15.64 to 19.16)
Spain	17.12	19.1	15.84	17.12 (15.84 to 19.1)
Qatar	16.92	19.09	15.02	16.92 (15.02 to 19.09)
Bulgaria	16.79	18.13	15.71	16.79 (15.71 to 18.13)
Iceland	15.72	17.62	13.86	15.72 (13.86 to 17.62)
Brunei Darussalam	15.66	17.07	14.34	15.66 (14.34 to 17.07)
Nauru	15.43	17.31	13.89	15.43 (13.89 to 17.31)
Palau	15.29	17.71	13.62	15.29 (13.62 to 17.71)
Greenland	14.95	16.87	13.24	14.95 (13.24 to 16.87)
Canada	14.82	15.7	14.09	14.82 (14.09 to 15.7)
Luxembourg	14.62	16.89	12.92	14.62 (12.92 to 16.89)
United Kingdom	14.45	16.53	12.55	14.45 (12.55 to 16.53)
Saudi Arabia	14.13	15.76	12.48	14.13 (12.48 to 15.76)
Monaco	14.1	16.26	12.16	14.1 (12.16 to 16.26)
Switzerland	13.67	15.56	11.81	13.67 (11.81 to 15.56)
Malta	13.67	15.72	11.96	13.67 (11.96 to 15.72)

France	13.66	14.85	12.57	13.66<br>(12.57 to 14.85)
Greece	13.49	15.15	11.92	13.49<br>(11.92 to 15.15)
Oman	13.44	15.05	11.78	13.44<br>(11.78 to 15.05)
Northern Mariana Islands	13.42	15.17	11.9	13.42<br>(11.9 to 15.17)
United States Virgin Islands	13.38	14.62	12.18	13.38<br>(12.18 to 14.62)
Austria	13.34	14.91	11.9	13.34<br>(11.9 to 14.91)
Argentina	13.29	14.36	12.25	13.29<br>(12.25 to 14.36)
Guam	13.29	15.66	11.53	13.29<br>(11.53 to 15.66)
Bosnia and Herzegovina	13.25	14.59	11.86	13.25<br>(11.86 to 14.59)
Andorra	13.24	15.52	11.38	13.24<br>(11.38 to 15.52)
San Marino	13.2	15.19	11.37	13.2<br>(11.37 to 15.19)
Marshall Islands	13.18	14.74	11.55	13.18<br>(11.55 to 14.74)
North Macedonia	13.17	14.72	11.73	13.17<br>(11.73 to 14.72)
Ireland	13.08	15.06	11.75	13.08<br>(11.75 to 15.06)
Bolivia (Plurinational State of)	13.08	14	12.15	13.08<br>(12.15 to 14)
Kiribati	13.05	14.44	11.78	13.05<br>(11.78 to 14.44)
Niue	12.96	14.51	11.43	12.96<br>(11.43 to 14.51)
Vanuatu	12.94	14.58	11.68	12.94<br>(11.68 to 14.58)
China	12.83	14.73	10.92	12.83<br>(10.92 to 14.73)
Belgium	12.68	14.41	11.47	12.68<br>(11.47 to 14.41)
Seychelles	12.67	14.15	11.51	12.67<br>(11.51 to 14.15)
High-middle SDI	12.66	14.06	11.27	12.66<br>(11.27 to 14.06)

Chile	12.62	13.71	11.68	12.62<br>(11.68 to 13.71)
Micronesia (Federated States of)	12.57	13.97	11.41	12.57<br>(11.41 to 13.97)
Montenegro	12.52	14.21	11.06	12.52<br>(11.06 to 14.21)
Egypt	12.5	13.95	11.37	12.5<br>(11.37 to 13.95)
Tajikistan	12.43	14.08	10.83	12.43<br>(10.83 to 14.08)
Cook Islands	12.41	14.03	11	12.41<br>(11 to 14.03)
Bahrain	12.28	13.7	10.92	12.28<br>(10.92 to 13.7)
Finland	12.27	13.95	11.06	12.27<br>(11.06 to 13.95)
Bermuda	12.26	13.38	11.23	12.26<br>(11.23 to 13.38)
Albania	12.24	13.62	10.89	12.24<br>(10.89 to 13.62)
Fiji	12.12	13.81	10.73	12.12<br>(10.73 to 13.81)
Uruguay	12.05	13.07	11.04	12.05<br>(11.04 to 13.07)
Solomon Islands	12	13.45	10.65	12<br>(10.65 to 13.45)
Honduras	11.85	13.07	10.82	11.85<br>(10.82 to 13.07)
Slovenia	11.81	12.94	10.76	11.81<br>(10.76 to 12.94)
Palestine	11.78	13.09	10.41	11.78<br>(10.41 to 13.09)
Samoa	11.77	13.17	10.39	11.77<br>(10.39 to 13.17)
Poland	11.77	13.66	9.99	11.77<br>(9.99 to 13.66)
Germany	11.74	12.2	11.14	11.74<br>(11.14 to 12.2)
Estonia	11.7	13.43	10.35	11.7<br>(10.35 to 13.43)
Croatia	11.68	13.16	10.43	11.68<br>(10.43 to 13.16)
Maldives	11.64	12.79	10.56	11.64<br>(10.56 to 12.79)

Serbia	11.6	12.61	10.43	11.6<br>(10.43 to 12.61)
Belarus	11.59	12.77	10.38	11.59<br>(10.38 to 12.77)
High SDI	11.4	12.56	10.35	11.4<br>(10.35 to 12.56)
Cameroon	11.39	12.52	10.26	11.39<br>(10.26 to 12.52)
Algeria	11.38	12.56	10.28	11.38<br>(10.28 to 12.56)
Peru	11.32	12.63	10.09	11.32<br>(10.09 to 12.63)
Ivoire	11.28	12.4	10.24	11.28<br>(10.24 to 12.4)
Thailand	11.27	12.5	10.2	11.27<br>(10.2 to 12.5)
Ukraine	11.27	13.14	9.66	11.27<br>(9.66 to 13.14)
Democratic People's Republic of Korea	11.26	12.76	10.03	11.26<br>(10.03 to 12.76)
Latvia	11.18	12.31	10.17	11.18<br>(10.17 to 12.31)
United States of America	11.13	12.89	9.39	11.13<br>(9.39 to 12.89)
Uzbekistan	10.92	12.41	9.56	10.92<br>(9.56 to 12.41)
Guinea-Bissau	10.9	12.02	9.8	10.9<br>(9.8 to 12.02)
Nicaragua	10.88	11.81	9.87	10.88<br>(9.87 to 11.81)
Middle SDI	10.88	12.26	9.48	10.88<br>(9.48 to 12.26)
Republic of Moldova	10.87	12.27	9.66	10.87<br>(9.66 to 12.27)
Senegal	10.77	11.77	9.69	10.77<br>(9.69 to 11.77)
Jordan	10.68	11.79	9.69	10.68<br>(9.69 to 11.79)
Pakistan	10.5	12.15	9.02	10.5<br>(9.02 to 12.15)
Zimbabwe	10.49	11.76	9.39	10.49<br>(9.39 to 11.76)
Romania	10.48	11.38	9.54	10.48<br>(9.54 to 11.38)

Slovakia	10.41	11.48	9.21	10.41<br>(9.21 to 11.48)
Saint Lucia	10.41	11.49	9.63	10.41<br>(9.63 to 11.49)
Afghanistan	10.41	11.47	9.44	10.41<br>(9.44 to 11.47)
Mauritania	10.39	11.46	9.36	10.39<br>(9.36 to 11.46)
Russian Federation	10.38	12.04	8.86	10.38<br>(8.86 to 12.04)
Saint Kitts and Nevis	10.38	11.32	9.48	10.38<br>(9.48 to 11.32)
Viet Nam	10.35	11.45	9.15	10.35<br>(9.15 to 11.45)
Guinea	10.35	11.42	9.15	10.35<br>(9.15 to 11.42)
Benin	10.3	11.4	9.29	10.3<br>(9.29 to 11.4)
Burkina Faso	10.27	11.31	9.24	10.27<br>(9.24 to 11.31)
Gabon	10.23	11.35	9.25	10.23<br>(9.25 to 11.35)
India	10.19	11.77	8.69	10.19<br>(8.69 to 11.77)
Georgia	10.16	11.29	9.13	10.16<br>(9.13 to 11.29)
Czechia	10.16	11.05	9.18	10.16<br>(9.18 to 11.05)
Portugal	10.15	11.42	8.92	10.15<br>(8.92 to 11.42)
Kazakhstan	10.13	11.48	8.87	10.13<br>(8.87 to 11.48)
Sao Tome and Principe	10.11	11.45	8.96	10.11<br>(8.96 to 11.45)
Costa Rica	10.1	11.12	9.02	10.1<br>(9.02 to 11.12)
Denmark	10.09	11.14	8.94	10.09<br>(8.94 to 11.14)
Azerbaijan	10.05	11.36	8.85	10.05<br>(8.85 to 11.36)
Tonga	10.05	11.4	8.72	10.05<br>(8.72 to 11.4)
Iran (Islamic Republic of)	10.04	11.56	8.5	10.04<br>(8.5 to 11.56)

Gambia	9.98	11.14	8.94	9.98<br>(8.94 to 11.14)
Low-middle SDI	9.95	11.1	8.79	9.95<br>(8.79 to 11.1)
Nigeria	9.94	11.51	8.4	9.94<br>(8.4 to 11.51)
Taiwan (Province of China)	9.93	10.42	9.36	9.93<br>(9.36 to 10.42)
Ecuador	9.9	11.23	8.93	9.9<br>(8.93 to 11.23)
Hungary	9.84	10.72	9.01	9.84<br>(9.01 to 10.72)
Malaysia	9.81	10.88	8.83	9.81<br>(8.83 to 10.88)
Liberia	9.78	10.84	8.86	9.78<br>(8.86 to 10.84)
Syrian Arab Republic	9.77	11.02	8.81	9.77<br>(8.81 to 11.02)
Haiti	9.75	10.52	9.07	9.75<br>(9.07 to 10.52)
Papua New Guinea	9.74	11.12	8.66	9.74<br>(8.66 to 11.12)
Australia	9.72	10.54	8.61	9.72<br>(8.61 to 10.54)
Guatemala	9.72	10.81	8.79	9.72<br>(8.79 to 10.81)
Tunisia	9.72	11.17	8.5	9.72<br>(8.5 to 11.17)
Bangladesh	9.71	10.85	8.78	9.71<br>(8.78 to 10.85)
Mongolia	9.69	10.82	8.81	9.69<br>(8.81 to 10.82)
Lithuania	9.65	11.25	8.69	9.65<br>(8.69 to 11.25)
Kuwait	9.65	10.89	8.72	9.65<br>(8.72 to 10.89)
Turkiye	9.6	10.7	8.63	9.6<br>(8.63 to 10.7)
Sri Lanka	9.59	10.69	8.52	9.59<br>(8.52 to 10.69)
Mauritius	9.59	10.74	8.72	9.59<br>(8.72 to 10.74)
Lebanon	9.57	10.72	8.57	9.57<br>(8.57 to 10.72)

Antigua and Barbuda	9.52	10.45	8.72	9.52<br>(8.72 to 10.45)
Dominica	9.51	10.37	8.68	9.51<br>(8.68 to 10.37)
Bahamas	9.49	10.39	8.66	9.49<br>(8.66 to 10.39)
Mexico	9.48	10.87	8.16	9.48<br>(8.16 to 10.87)
Togo	9.48	10.44	8.56	9.48<br>(8.56 to 10.44)
Philippines	9.45	10.84	8.05	9.45<br>(8.05 to 10.84)
Sudan	9.44	10.44	8.52	9.44<br>(8.52 to 10.44)
Iraq	9.43	10.63	8.4	9.43<br>(8.4 to 10.63)
Sweden	9.37	10.89	8	9.37<br>(8 to 10.89)
Sierra Leone	9.3	10.17	8.37	9.3<br>(8.37 to 10.17)
Brazil	9.29	10.7	7.9	9.29<br>(7.9 to 10.7)
Lao People's Democratic Republic	9.27	10.06	8.46	9.27<br>(8.46 to 10.06)
Panama	9.22	10.07	8.38	9.22<br>(8.38 to 10.07)
Congo	9.21	10.16	8.24	9.21<br>(8.24 to 10.16)
Guyana	9.17	9.96	8.48	9.17<br>(8.48 to 9.96)
Chad	9.13	10.15	8.22	9.13<br>(8.22 to 10.15)
Morocco	9.09	10.3	8.15	9.09<br>(8.15 to 10.3)
Niger	9.08	10.08	8.09	9.08<br>(8.09 to 10.08)
Low SDI	9.08	10.06	8.18	9.08<br>(8.18 to 10.06)
Armenia	9.05	9.87	8.21	9.05<br>(8.21 to 9.87)
Libya	9	10.04	8.09	9<br>(8.09 to 10.04)
Kyrgyzstan	8.98	9.89	8.09	8.98<br>(8.09 to 9.89)

El Salvador	8.98	9.85	8.11	8.98<br>(8.11 to 9.85)
Puerto Rico	8.98	9.57	8.05	8.98<br>(8.05 to 9.57)
New Zealand	8.93	10.42	7.49	8.93<br>(7.49 to 10.42)
Barbados	8.92	9.81	8.19	8.92<br>(8.19 to 9.81)
Bhutan	8.85	9.99	7.8	8.85<br>(7.8 to 9.99)
Mali	8.83	9.76	8	8.83<br>(8 to 9.76)
Colombia	8.76	9.59	7.87	8.76<br>(7.87 to 9.59)
Eswatini	8.76	9.8	7.92	8.76<br>(7.92 to 9.8)
Zambia	8.68	9.51	7.86	8.68<br>(7.86 to 9.51)
Venezuela (Bolivarian Republic of)	8.67	9.7	7.89	8.67<br>(7.89 to 9.7)
Belize	8.65	9.46	7.91	8.65<br>(7.91 to 9.46)
American Samoa	8.65	9.88	7.37	8.65<br>(7.37 to 9.88)
Paraguay	8.6	9.66	7.67	8.6<br>(7.67 to 9.66)
Indonesia	8.56	9.79	7.26	8.56<br>(7.26 to 9.79)
Namibia	8.54	9.5	7.62	8.54<br>(7.62 to 9.5)
Timor-Leste	8.52	9.42	7.68	8.52<br>(7.68 to 9.42)
Cabo Verde	8.52	9.64	7.49	8.52<br>(7.49 to 9.64)
Singapore	8.47	9.31	7.75	8.47<br>(7.75 to 9.31)
Botswana	8.44	9.49	7.48	8.44<br>(7.48 to 9.49)
Saint Vincent and the Grenadines	8.42	9.1	7.69	8.42<br>(7.69 to 9.1)
Comoros	8.39	9.4	7.54	8.39<br>(7.54 to 9.4)
Trinidad and Tobago	8.36	9.06	7.67	8.36<br>(7.67 to 9.06)

Myanmar	8.33	9.17	7.53	8.33<br>(7.53 to 9.17)
Ethiopia	8.33	9.57	7.09	8.33<br>(7.09 to 9.57)
Ghana	8.3	9.27	7.36	8.3<br>(7.36 to 9.27)
Central African Republic	8.28	9.21	7.5	8.28<br>(7.5 to 9.21)
Cambodia	8.22	9	7.43	8.22<br>(7.43 to 9)
Grenada	8.2	9.1	7.54	8.2<br>(7.54 to 9.1)
Jamaica	8.19	8.89	7.45	8.19<br>(7.45 to 8.89)
Uganda	8.18	9.15	7.31	8.18<br>(7.31 to 9.15)
Rwanda	8.13	9.02	7.3	8.13<br>(7.3 to 9.02)
Equatorial Guinea	8.05	8.91	7.27	8.05<br>(7.27 to 8.91)
Burundi	8.03	8.99	7.28	8.03<br>(7.28 to 8.99)
Angola	8.02	8.93	7.23	8.02<br>(7.23 to 8.93)
Japan	8.01	9.29	6.83	8.01<br>(6.83 to 9.29)
Malawi	7.93	8.79	7.16	7.93<br>(7.16 to 8.79)
Dominican Republic	7.91	8.76	7.24	7.91<br>(7.24 to 8.76)
Kenya	7.89	9.15	6.67	7.89<br>(6.67 to 9.15)
Turkmenistan	7.88	8.79	7	7.88<br>(7 to 8.79)
South Africa	7.87	9.1	6.69	7.87<br>(6.69 to 9.1)
Eritrea	7.81	8.76	7.04	7.81<br>(7.04 to 8.76)
Mozambique	7.76	8.76	6.99	7.76<br>(6.99 to 8.76)
South Sudan	7.74	8.7	6.88	7.74<br>(6.88 to 8.7)
Yemen	7.72	8.57	6.96	7.72<br>(6.96 to 8.57)

Democratic Republic of the Congo	7.7	8.56	6.9	7.7<br>(6.9 to 8.56)
Cuba	7.69	8.34	7.06	7.69<br>(7.06 to 8.34)
Nepal	7.67	8.63	6.86	7.67<br>(6.86 to 8.63)
Djibouti	7.6	8.52	6.74	7.6<br>(6.74 to 8.52)
Republic of Korea	7.46	8.36	6.67	7.46<br>(6.67 to 8.36)
United Republic of Tanzania	7.44	8.23	6.7	7.44<br>(6.7 to 8.23)
Somalia	7.4	8.28	6.69	7.4<br>(6.69 to 8.28)
Suriname	7.3	7.98	6.66	7.3<br>(6.66 to 7.98)
Lesotho	7.06	7.91	6.26	7.06<br>(6.26 to 7.91)
Madagascar	6.89	7.84	6.13	6.89<br>(6.13 to 7.84)
Norway	5.02	5.76	4.3	5.02<br>(4.3 to 5.76)

Table S2: ASIR of PD by country in 2021

location	val	upper	lower	Rate_2021
China	24.34	28.3	20.67	24.34<br>(20.67 to 28.3)
Qatar	24.22	27.95	21.16	24.22<br>(21.16 to 27.95)
Israel	21.87	26.12	18.51	21.87<br>(18.51 to 26.12)
Germany	21.53	22.31	20.78	21.53<br>(20.78 to 22.31)
Taiwan (Province of China)	21.29	22.24	20.47	21.29<br>(20.47 to 22.24)
Canada	21.05	21.99	20.04	21.05<br>(20.04 to 21.99)
Oman	20.18	22.94	17.24	20.18<br>(17.24 to 22.94)
Iceland	20.15	23.4	17.31	20.15<br>(17.31 to 23.4)
Spain	19.97	22.74	17.46	19.97<br>(17.46 to 22.74)
United Arab Emirates	19.56	22.48	17.31	19.56<br>(17.31 to 22.48)

Netherlands	19.11	21.25	17.14	19.11<br>(17.14 to 21.25)
Bolivia (Plurinational State of)	19.09	21.21	17.03	19.09<br>(17.03 to 21.21)
Saudi Arabia	18.89	21.72	16.58	18.89<br>(16.58 to 21.72)
Luxembourg	18.52	21.21	15.91	18.52<br>(15.91 to 21.21)
High-middle SDI	18.49	21.03	16.31	18.49<br>(16.31 to 21.03)
Monaco	18.21	21.81	15.46	18.21<br>(15.46 to 21.81)
Cyprus	18.03	21.26	15.39	18.03<br>(15.39 to 21.26)
Honduras	18	20.02	16.19	18<br>(16.19 to 20.02)
Ireland	17.64	19.83	15.16	17.64<br>(15.16 to 19.83)
Tajikistan	17.55	19.72	15.63	17.55<br>(15.63 to 19.72)
Malta	17.55	20.12	15.74	17.55<br>(15.74 to 20.12)
France	17.49	19.7	14.47	17.49<br>(14.47 to 19.7)
Egypt	17.28	19.07	15.53	17.28<br>(15.53 to 19.07)
Finland	17.23	20.92	14.77	17.23<br>(14.77 to 20.92)
Austria	17.11	20.34	13.81	17.11<br>(13.81 to 20.34)
Democratic People's Republic of Korea	17.05	20.14	14.77	17.05<br>(14.77 to 20.14)
Switzerland	16.95	19.67	14.49	16.95<br>(14.49 to 19.67)
Greenland	16.95	18.91	14.95	16.95<br>(14.95 to 18.91)
Middle SDI	16.87	19.11	14.67	16.87<br>(14.67 to 19.11)
Ecuador	16.63	18.75	14.57	16.63<br>(14.57 to 18.75)
United Kingdom	16.6	18.77	14.43	16.6<br>(14.43 to 18.77)
Brunei Darussalam	16.48	18.44	15.03	16.48<br>(15.03 to 18.44)

Bahrain	16.28	18.27	14.53	16.28<br>(14.53 to 18.27)
Belgium	16.24	18.14	14.45	16.24<br>(14.45 to 18.14)
Peru	16.21	18.6	14.24	16.21<br>(14.24 to 18.6)
Greece	15.95	18.35	14.05	15.95<br>(14.05 to 18.35)
United States Virgin Islands	15.89	17.24	14.59	15.89<br>(14.59 to 17.24)
Andorra	15.85	18.38	14.07	15.85<br>(14.07 to 18.38)
Chile	15.69	17.35	13.98	15.69<br>(13.98 to 17.35)
Denmark	15.53	18.04	13.14	15.53<br>(13.14 to 18.04)
Palau	15.36	17.61	13.44	15.36<br>(13.44 to 17.61)
Seychelles	15.31	17.15	13.85	15.31<br>(13.85 to 17.15)
High SDI	14.99	16.04	13.97	14.99<br>(13.97 to 16.04)
North Macedonia	14.93	16.55	13.51	14.93<br>(13.51 to 16.55)
Nauru	14.9	16.36	13.35	14.9<br>(13.35 to 16.36)
Nicaragua	14.89	16.43	13.29	14.89<br>(13.29 to 16.43)
Sweden	14.74	17.05	12.36	14.74<br>(12.36 to 17.05)
Uruguay	14.68	16.41	13.28	14.68<br>(13.28 to 16.41)
San Marino	14.55	17.82	11.98	14.55<br>(11.98 to 17.82)
Palestine	14.27	15.91	12.61	14.27<br>(12.61 to 15.91)
United States of America	14.25	15.52	13.04	14.25<br>(13.04 to 15.52)
Marshall Islands	14.23	16.17	12.59	14.23<br>(12.59 to 16.17)
Italy	13.93	15.96	11.83	13.93<br>(11.83 to 15.96)
Northern Mariana Islands	13.87	15.51	12.32	13.87<br>(12.32 to 15.51)

Viet Nam	13.77	15.61	12.33	13.77<br>(12.33 to 15.61)
Argentina	13.67	15.75	12.39	13.67<br>(12.39 to 15.75)
Kiribati	13.58	15.02	12.45	13.58<br>(12.45 to 15.02)
Portugal	13.54	15.25	11.79	13.54<br>(11.79 to 15.25)
Micronesia (Federated States of)	13.47	14.87	12.1	13.47<br>(12.1 to 14.87)
Bulgaria	13.46	14.86	12.23	13.46<br>(12.23 to 14.86)
Uzbekistan	13.38	14.48	12.31	13.38<br>(12.31 to 14.48)
Montenegro	13.26	14.55	11.79	13.26<br>(11.79 to 14.55)
Niue	13.26	15.03	11.6	13.26<br>(11.6 to 15.03)
Azerbaijan	13.12	14.63	11.63	13.12<br>(11.63 to 14.63)
Iran (Islamic Republic of)	13.05	14.91	11.16	13.05<br>(11.16 to 14.91)
Albania	13.03	14.44	11.43	13.03<br>(11.43 to 14.44)
Iraq	13.02	14.96	11.4	13.02<br>(11.4 to 14.96)
Morocco	13	14.38	11.63	13<br>(11.63 to 14.38)
Turkiye	12.97	14.88	11.36	12.97<br>(11.36 to 14.88)
Algeria	12.97	14.57	11.56	12.97<br>(11.56 to 14.57)
Sao Tome and Principe	12.97	14.55	11.59	12.97<br>(11.59 to 14.55)
Vanuatu	12.96	14.8	11.64	12.96<br>(11.64 to 14.8)
Syrian Arab Republic	12.93	14.59	11.49	12.93<br>(11.49 to 14.59)
Bosnia and Herzegovina	12.89	14.35	11.36	12.89<br>(11.36 to 14.35)
Serbia	12.85	14.21	11.31	12.85<br>(11.31 to 14.21)
Bermuda	12.83	14.13	11.66	12.83<br>(11.66 to 14.13)

Maldives	12.66	14.4	11.26	12.66 (11.26 to 14.4)
Senegal	12.6	13.96	11.46	12.6 (11.46 to 13.96)
Cook Islands	12.6	14.96	10.8	12.6 (10.8 to 14.96)
Antigua and Barbuda	12.58	14.04	11.45	12.58 (11.45 to 14.04)
Poland	12.56	13.89	11.33	12.56 (11.33 to 13.89)
Kazakhstan	12.56	13.76	11.32	12.56 (11.32 to 13.76)
Croatia	12.55	13.83	11.13	12.55 (11.13 to 13.83)
Solomon Islands	12.54	14.07	11.2	12.54 (11.2 to 14.07)
Panama	12.52	14.31	11.03	12.52 (11.03 to 14.31)
Cameroon	12.51	14.07	11.23	12.51 (11.23 to 14.07)
Nigeria	12.49	14.29	10.66	12.49 (10.66 to 14.29)
Pakistan	12.45	14.16	10.71	12.45 (10.71 to 14.16)
Samoa	12.44	13.81	11.07	12.44 (11.07 to 13.81)
Belarus	12.42	13.64	11.25	12.42 (11.25 to 13.64)
Ivoire	12.42	13.71	11.19	12.42 (11.19 to 13.71)
Mexico	12.35	14	10.66	12.35 (10.66 to 14)
Gambia	12.29	13.8	10.94	12.29 (10.94 to 13.8)
Costa Rica	12.28	14.14	10.76	12.28 (10.76 to 14.14)
Bhutan	12.28	14.05	10.69	12.28 (10.69 to 14.05)
Tunisia	12.26	13.69	10.56	12.26 (10.56 to 13.69)
Slovenia	12.22	13.49	10.59	12.22 (10.59 to 13.49)
Fiji	12.16	13.78	10.46	12.16 (10.46 to 13.78)

Australia	12.13	13.83	11.03	12.13<br>(11.03 to 13.83)
Norway	12.02	13.8	10.27	12.02<br>(10.27 to 13.8)
Cabo Verde	11.97	13.45	10.56	11.97<br>(10.56 to 13.45)
Guam	11.97	14.51	10.72	11.97<br>(10.72 to 14.51)
Low-middle SDI	11.95	13.14	10.71	11.95<br>(10.71 to 13.14)
Malaysia	11.92	13.6	10.55	11.92<br>(10.55 to 13.6)
India	11.91	13.5	10.31	11.91<br>(10.31 to 13.5)
El Salvador	11.9	13.27	10.59	11.9<br>(10.59 to 13.27)
Sudan	11.83	13.29	10.37	11.83<br>(10.37 to 13.29)
Guinea-Bissau	11.82	13.17	10.64	11.82<br>(10.64 to 13.17)
Guinea	11.75	12.93	10.6	11.75<br>(10.6 to 12.93)
Saint Kitts and Nevis	11.71	12.87	10.67	11.71<br>(10.67 to 12.87)
Gabon	11.66	12.99	10.55	11.66<br>(10.55 to 12.99)
Thailand	11.64	12.92	10.46	11.64<br>(10.46 to 12.92)
Libya	11.58	12.86	10.3	11.58<br>(10.3 to 12.86)
Afghanistan	11.56	12.69	10.33	11.56<br>(10.33 to 12.69)
Czechia	11.55	12.87	10.46	11.55<br>(10.46 to 12.87)
Romania	11.54	12.54	10.54	11.54<br>(10.54 to 12.54)
Lebanon	11.54	13.01	10.16	11.54<br>(10.16 to 13.01)
Estonia	11.53	13.04	10.23	11.53<br>(10.23 to 13.04)
Saint Lucia	11.53	12.64	10.43	11.53<br>(10.43 to 12.64)
Benin	11.48	12.59	10.24	11.48<br>(10.24 to 12.59)

Sri Lanka	11.42	13.18	9.91	11.42 (9.91 to 13.18)
Ukraine	11.36	13	9.68	11.36 (9.68 to 13)
Jordan	11.35	12.42	10.38	11.35 (10.38 to 12.42)
Kuwait	11.31	13.23	9.4	11.31 (9.4 to 13.23)
Mauritania	11.3	12.96	10.06	11.3 (10.06 to 12.96)
Zimbabwe	11.25	12.52	10.01	11.25 (10.01 to 12.52)
Dominica	11.15	12.13	10.15	11.15 (10.15 to 12.13)
Indonesia	11.14	12.67	9.58	11.14 (9.58 to 12.67)
Barbados	11.14	12.31	10.07	11.14 (10.07 to 12.31)
Burkina Faso	11.13	12.26	10.14	11.13 (10.14 to 12.26)
Mauritius	11.1	12.37	9.67	11.1 (9.67 to 12.37)
Equatorial Guinea	11.09	12.56	9.86	11.09 (9.86 to 12.56)
Venezuela (Bolivarian Republic of)	11.08	12.23	10.03	11.08 (10.03 to 12.23)
Bahamas	11.06	11.95	10.21	11.06 (10.21 to 11.95)
Latvia	11.04	12.28	10	11.04 (10 to 12.28)
Republic of Korea	11.01	12.19	9.77	11.01 (9.77 to 12.19)
Colombia	10.93	12.5	9.58	10.93 (9.58 to 12.5)
Guatemala	10.92	12.18	9.61	10.92 (9.61 to 12.18)
Tonga	10.89	12.56	9.56	10.89 (9.56 to 12.56)
Brazil	10.84	12.31	9.38	10.84 (9.38 to 12.31)
Nepal	10.78	12.2	9.56	10.78 (9.56 to 12.2)
Russian Federation	10.77	12.33	9.24	10.77 (9.24 to 12.33)

Hungary	10.67	11.78	9.86	10.67<br>(9.86 to 11.78)
Timor-Leste	10.66	11.88	9.54	10.66<br>(9.54 to 11.88)
Slovakia	10.63	11.89	9.53	10.63<br>(9.53 to 11.89)
Lithuania	10.61	11.62	9.4	10.61<br>(9.4 to 11.62)
Grenada	10.59	11.69	9.62	10.59<br>(9.62 to 11.69)
Togo	10.57	12.04	9.13	10.57<br>(9.13 to 12.04)
Bangladesh	10.54	11.85	9.37	10.54<br>(9.37 to 11.85)
Papua New Guinea	10.53	11.93	9.19	10.53<br>(9.19 to 11.93)
Haiti	10.53	11.4	9.78	10.53<br>(9.78 to 11.4)
Liberia	10.49	11.52	9.34	10.49<br>(9.34 to 11.52)
Belize	10.46	11.41	9.6	10.46<br>(9.6 to 11.41)
Lao People's Democratic Republic	10.44	11.6	9.36	10.44<br>(9.36 to 11.6)
Botswana	10.36	11.65	9.25	10.36<br>(9.25 to 11.65)
Paraguay	10.34	11.46	9.14	10.34<br>(9.14 to 11.46)
Sierra Leone	10.33	11.3	9.26	10.33<br>(9.26 to 11.3)
Myanmar	10.29	11.28	9.3	10.29<br>(9.3 to 11.28)
Dominican Republic	10.28	11.16	9.35	10.28<br>(9.35 to 11.16)
Guyana	10.28	11.05	9.56	10.28<br>(9.56 to 11.05)
Eswatini	10.27	11.42	9.14	10.27<br>(9.14 to 11.42)
Chad	10.26	11.54	9.22	10.26<br>(9.22 to 11.54)
Ghana	10.23	11.48	9.18	10.23<br>(9.18 to 11.48)
Low SDI	10.21	11.29	9.2	10.21<br>(9.2 to 11.29)

Singapore	10.2	11.52	8.96	10.2 (8.96 to 11.52)
Yemen	10.2	11.3	9.08	10.2 (9.08 to 11.3)
New Zealand	10.17	11.71	8.67	10.17 (8.67 to 11.71)
Saint Vincent and the Grenadines	10.17	11.15	9.37	10.17 (9.37 to 11.15)
Congo	10.06	11.17	8.98	10.06 (8.98 to 11.17)
Puerto Rico	10.01	10.98	9.01	10.01 (9.01 to 10.98)
Namibia	10	11.14	8.94	10 (8.94 to 11.14)
Philippines	9.95	11.3	8.5	9.95 (8.5 to 11.3)
Cambodia	9.94	11.03	8.95	9.94 (8.95 to 11.03)
Armenia	9.89	10.93	8.83	9.89 (8.83 to 10.93)
Georgia	9.86	10.8	9.06	9.86 (9.06 to 10.8)
Niger	9.74	10.96	8.67	9.74 (8.67 to 10.96)
South Africa	9.65	11.07	8.2	9.65 (8.2 to 11.07)
Mali	9.62	10.56	8.72	9.62 (8.72 to 10.56)
Jamaica	9.53	10.62	8.72	9.53 (8.72 to 10.62)
Cuba	9.47	10.3	8.78	9.47 (8.78 to 10.3)
Lesotho	9.2	10.28	8.28	9.2 (8.28 to 10.28)
Zambia	9.17	10.2	8.15	9.17 (8.15 to 10.2)
Trinidad and Tobago	9.15	10.45	8.35	9.15 (8.35 to 10.45)
Kenya	9.15	10.38	7.8	9.15 (7.8 to 10.38)
Djibouti	9.13	10.07	8.13	9.13 (8.13 to 10.07)
Mongolia	9.05	10.26	8.15	9.05 (8.15 to 10.26)

Uganda	9.05	10	8.06	9.05<br>(8.06 to 10)
Comoros	9.03	10.11	8.01	9.03<br>(8.01 to 10.11)
Angola	9	10.08	7.99	9<br>(7.99 to 10.08)
Eritrea	8.93	9.83	8.03	8.93<br>(8.03 to 9.83)
Suriname	8.92	9.79	8.1	8.92<br>(8.1 to 9.79)
American Samoa	8.9	10.16	7.6	8.9<br>(7.6 to 10.16)
Kyrgyzstan	8.89	9.86	7.92	8.89<br>(7.92 to 9.86)
Republic of Moldova	8.82	10.04	7.8	8.82<br>(7.8 to 10.04)
United Republic of Tanzania	8.77	9.45	8.21	8.77<br>(8.21 to 9.45)
Ethiopia	8.76	10.05	7.47	8.76<br>(7.47 to 10.05)
Mozambique	8.74	9.76	7.83	8.74<br>(7.83 to 9.76)
Malawi	8.56	9.46	7.62	8.56<br>(7.62 to 9.46)
Central African Republic	8.5	9.43	7.69	8.5<br>(7.69 to 9.43)
Burundi	8.48	9.4	7.58	8.48<br>(7.58 to 9.4)
Rwanda	8.32	9.31	7.48	8.32<br>(7.48 to 9.31)
Turkmenistan	8.3	9.1	7.45	8.3<br>(7.45 to 9.1)
Democratic Republic of the Congo	8.08	9.06	7.21	8.08<br>(7.21 to 9.06)
South Sudan	7.95	8.96	6.9	7.95<br>(6.9 to 8.96)
Somalia	7.52	8.44	6.72	7.52<br>(6.72 to 8.44)
Madagascar	7.49	8.37	6.61	7.49<br>(6.61 to 8.37)
Japan	7.28	8.28	6.29	7.28<br>(6.29 to 8.28)

Table S3: ASPR of PD by country in 1990

location	val	upper	lower	Rate_1990

Israel	164.48	199.54	138.47	164.48 (138.47 to 199.54)
Italy	160.72	187.75	136.22	160.72 (136.22 to 187.75)
Netherlands	162.18	181.79	143.17	162.18 (143.17 to 181.79)
Spain	144.93	160.98	131.43	144.93 (131.43 to 160.98)
Cyprus	127.45	152.96	104.07	127.45 (104.07 to 152.96)
United Kingdom	128.91	150.75	110.51	128.91 (110.51 to 150.75)
Iceland	128.23	150.68	108.98	128.23 (108.98 to 150.68)
Bulgaria	125.96	141.59	114.04	125.96 (114.04 to 141.59)
Luxembourg	115.92	140.6	97.56	115.92 (97.56 to 140.6)
San Marino	119.54	140.07	100.38	119.54 (100.38 to 140.07)
Monaco	115.42	136.78	94.19	115.42 (94.19 to 136.78)
Estonia	109.64	133.78	92.14	109.64 (92.14 to 133.78)
Canada	124.09	133.52	116.2	124.09 (116.2 to 133.52)
Greece	110.12	131.62	91.92	110.12 (91.92 to 131.62)
Switzerland	112.59	131.31	94.3	112.59 (94.3 to 131.31)
Malta	107.09	130.17	87.84	107.09 (87.84 to 130.17)
Andorra	108.47	129.62	88.09	108.47 (88.09 to 129.62)
Palau	106.44	128.71	87.07	106.44 (87.07 to 128.71)
Austria	107.34	126.54	91.75	107.34 (91.75 to 126.54)
Guam	101.71	125.13	82.06	101.71 (82.06 to 125.13)
Ireland	105.04	123.06	90.78	105.04 (90.78 to 123.06)
Qatar	101.06	122.2	80.21	101.06 (80.21 to 122.2)

France	108.48	121.46	94.78	108.48 (94.78 to 121.46)
Belgium	102.59	119.37	89.53	102.59 (89.53 to 119.37)
Nauru	95.65	118.94	77.28	95.65 (77.28 to 118.94)
Northern Mariana Islands	101.06	118.92	84.7	101.06 (84.7 to 118.92)
Ukraine	98.16	118.28	80.76	98.16 (80.76 to 118.28)
Finland	101.28	118.21	87.52	101.28 (87.52 to 118.21)
High-middle SDI	102.66	117.98	90.25	102.66 (90.25 to 117.98)
Bosnia and Herzegovina	99.12	116.87	80.56	99.12 (80.56 to 116.87)
Belarus	97.97	116.21	81.51	97.97 (81.51 to 116.21)
United Arab Emirates	97.37	115.61	78.61	97.37 (78.61 to 115.61)
Montenegro	98.14	115.09	80.48	98.14 (80.48 to 115.09)
Greenland	93.74	114.88	75.05	93.74 (75.05 to 114.88)
Niue	93.29	112.81	76.25	93.29 (76.25 to 112.81)
Cook Islands	92.83	112.44	76.56	92.83 (76.56 to 112.44)
Slovenia	96.52	111.39	83.6	96.52 (83.6 to 111.39)
North Macedonia	92.23	110.2	76.15	92.23 (76.15 to 110.2)
Republic of Moldova	91.41	110.09	74.83	91.41 (74.83 to 110.09)
Peru	92.93	110.01	78.92	92.93 (78.92 to 110.01)
China	91.77	109.65	75.88	91.77 (75.88 to 109.65)
Croatia	90.61	108.52	75.04	90.61 (75.04 to 108.52)
Argentina	97.51	108.28	85.2	97.51 (85.2 to 108.28)
Albania	90.43	108.19	73.89	90.43 (73.89 to 108.19)

Latvia	94.12	108.12	80.94	94.12<br>(80.94 to 108.12)
Poland	89.15	107.5	73.62	89.15<br>(73.62 to 107.5)
Marshall Islands	87.3	105.93	70.66	87.3<br>(70.66 to 105.93)
Bolivia (Plurinational State of)	94.86	105.51	84.55	94.86<br>(84.55 to 105.51)
Lithuania	88.01	105.4	74.61	88.01<br>(74.61 to 105.4)
Russian Federation	87.24	105.2	72.21	87.24<br>(72.21 to 105.2)
United States of America	86.92	104.03	72.15	86.92<br>(72.15 to 104.03)
High SDI	91.5	103.08	81.85	91.5<br>(81.85 to 103.08)
Vanuatu	86.52	103.06	72.17	86.52<br>(72.17 to 103.06)
Egypt	85.39	101.49	70.68	85.39<br>(70.68 to 101.49)
Tajikistan	83.53	101.41	66.25	83.53<br>(66.25 to 101.41)
Samoa	84.58	100.35	68.93	84.58<br>(68.93 to 100.35)
Fiji	84.35	100.3	68.85	84.35<br>(68.85 to 100.3)
Germany	93.95	100.28	87.75	93.95<br>(87.75 to 100.28)
Ecuador	82.42	100.28	69.22	82.42<br>(69.22 to 100.28)
Seychelles	83.73	99.96	70.96	83.73<br>(70.96 to 99.96)
Democratic People's Republic of Korea	82.9	99.37	68.43	82.9<br>(68.43 to 99.37)
Serbia	85.55	99.21	71.59	85.55<br>(71.59 to 99.21)
Chile	86.22	98.16	74.94	86.22<br>(74.94 to 98.16)
Georgia	81.89	97.9	68.64	81.89<br>(68.64 to 97.9)
Saudi Arabia	81.2	97.59	64.61	81.2<br>(64.61 to 97.59)
Uzbekistan	80.34	97.56	64.75	80.34<br>(64.75 to 97.56)

Uruguay	85.64	96.43	73.96	85.64<br>(73.96 to 96.43)
Oman	80.21	95.99	63.34	80.21<br>(63.34 to 95.99)
Slovakia	81.08	95.74	67.3	81.08<br>(67.3 to 95.74)
United States Virgin Islands	80.42	95.46	65.27	80.42<br>(65.27 to 95.46)
Micronesia (Federated States of)	79.94	95.44	67.08	79.94<br>(67.08 to 95.44)
Solomon Islands	78.43	94.5	63.4	78.43<br>(63.4 to 94.5)
Bermuda	79.97	94.43	67.71	79.97<br>(67.71 to 94.43)
Kiribati	78.84	94.37	64.8	78.84<br>(64.8 to 94.37)
Costa Rica	80.52	94.3	67	80.52<br>(67 to 94.3)
Denmark	81.31	93.57	67.97	81.31<br>(67.97 to 93.57)
Czechia	80.38	93.3	66.51	80.38<br>(66.51 to 93.3)
Kazakhstan	76.31	91.57	61.77	76.31<br>(61.77 to 91.57)
Portugal	77.99	91.5	64.74	77.99<br>(64.74 to 91.5)
Bahrain	76.74	91.45	62.89	76.74<br>(62.89 to 91.45)
Azerbaijan	75.27	91.33	62.05	75.27<br>(62.05 to 91.33)
Sweden	76.54	91.2	63.9	76.54<br>(63.9 to 91.2)
Thailand	75.09	91.05	62.87	75.09<br>(62.87 to 91.05)
Tonga	76.53	90.3	62.17	76.53<br>(62.17 to 90.3)
Maldives	76.15	89.68	62.81	76.15<br>(62.81 to 89.68)
Middle SDI	76.14	89.48	65.02	76.14<br>(65.02 to 89.48)
Brunei Darussalam	74.68	88.47	61.65	74.68<br>(61.65 to 88.47)
Nicaragua	76.64	87.4	65.09	76.64<br>(65.09 to 87.4)

Honduras	73.05	87.12	60.75	73.05<br>(60.75 to 87.12)
Palestine	72.91	86.81	58.42	72.91<br>(58.42 to 86.81)
Romania	74.9	86.44	62.42	74.9<br>(62.42 to 86.44)
Viet Nam	71.57	86.1	58.54	71.57<br>(58.54 to 86.1)
Papua New Guinea	70	85.75	57.5	70<br>(57.5 to 85.75)
Hungary	75.61	85.62	65.44	75.61<br>(65.44 to 85.62)
Algeria	72.07	85.43	59.62	72.07<br>(59.62 to 85.43)
Kuwait	72.71	85.21	61.89	72.71<br>(61.89 to 85.21)
Panama	73.77	84.6	62.17	73.77<br>(62.17 to 84.6)
Armenia	72.87	84.11	63.21	72.87<br>(63.21 to 84.11)
Mauritius	71.17	83.79	61.04	71.17<br>(61.04 to 83.79)
Kyrgyzstan	70.61	83.69	58.98	70.61<br>(58.98 to 83.69)
Mongolia	67.66	83.44	56.2	67.66<br>(56.2 to 83.44)
Sri Lanka	70.87	83.24	57.72	70.87<br>(57.72 to 83.24)
Malaysia	69.71	82.83	57.05	69.71<br>(57.05 to 82.83)
American Samoa	69.39	82.79	57.22	69.39<br>(57.22 to 82.79)
Iran (Islamic Republic of)	69.29	82.47	57.92	69.29<br>(57.92 to 82.47)
Pakistan	68.53	81.9	57.06	68.53<br>(57.06 to 81.9)
Tunisia	66.31	81.53	53.53	66.31<br>(53.53 to 81.53)
Paraguay	67.36	80.94	55.55	67.36<br>(55.55 to 80.94)
Brazil	68.18	80.62	57.72	68.18<br>(57.72 to 80.62)
Colombia	67.81	79.72	56.54	67.81<br>(56.54 to 79.72)

Mexico	67.38	79.5	57.19	67.38<br>(57.19 to 79.5)
El Salvador	67.4	78.91	57.7	67.4<br>(57.7 to 78.91)
Philippines	65.83	78.88	54.5	65.83<br>(54.5 to 78.88)
India	65.86	78.69	54.89	65.86<br>(54.89 to 78.69)
Ivoire	65.74	78.23	54.5	65.74<br>(54.5 to 78.23)
Gabon	65.13	78.16	54.36	65.13<br>(54.36 to 78.16)
Australia	66.74	78.06	54.31	66.74<br>(54.31 to 78.06)
Bangladesh	65.33	77.96	52.97	65.33<br>(52.97 to 77.96)
Japan	65.5	77.64	55.39	65.5<br>(55.39 to 77.64)
Lebanon	66.02	77.53	54.51	66.02<br>(54.51 to 77.53)
Cameroon	66.09	77.4	53.84	66.09<br>(53.84 to 77.4)
Turkmenistan	63.74	77.26	52.61	63.74<br>(52.61 to 77.26)
Bahamas	65.04	77.16	54.97	65.04<br>(54.97 to 77.16)
Bhutan	62.78	76.87	49.76	62.78<br>(49.76 to 76.87)
Zimbabwe	64.04	76.61	52.5	64.04<br>(52.5 to 76.61)
Saint Lucia	64.49	76.44	54.36	64.49<br>(54.36 to 76.44)
Sao Tome and Principe	64.02	76.39	51.91	64.02<br>(51.91 to 76.39)
Low-middle SDI	65.78	76.2	56.26	65.78<br>(56.26 to 76.2)
Turkiye	63.18	76.15	52.73	63.18<br>(52.73 to 76.15)
Puerto Rico	66.44	76.12	56.32	66.44<br>(56.32 to 76.12)
Venezuela (Bolivarian Republic of)	65.37	76.06	55.11	65.37<br>(55.11 to 76.06)
Guatemala	62.77	75.95	52.65	62.77<br>(52.65 to 75.95)

Antigua and Barbuda	64.77	75.52	55.63	64.77 (55.63 to 75.52)
Senegal	62.34	74.96	50.8	62.34 (50.8 to 74.96)
Iraq	61.89	74.93	51.23	61.89 (51.23 to 74.93)
Syrian Arab Republic	62.28	74.69	52.16	62.28 (52.16 to 74.69)
Afghanistan	61.46	74.49	51.73	61.46 (51.73 to 74.49)
Guinea-Bissau	62.05	74.48	49.72	62.05 (49.72 to 74.48)
Nigeria	61.63	73.86	51.11	61.63 (51.11 to 73.86)
Saint Kitts and Nevis	62.09	73.28	52.22	62.09 (52.22 to 73.28)
Liberia	59.5	72.95	48.64	59.5 (48.64 to 72.95)
Libya	62.55	72.67	52.06	62.55 (52.06 to 72.67)
Barbados	61.91	72.6	53.05	61.91 (53.05 to 72.6)
Jordan	62.2	72.41	51.57	62.2 (51.57 to 72.41)
Mauritania	60.52	72.41	49.56	60.52 (49.56 to 72.41)
Benin	61.08	72.26	50.22	61.08 (50.22 to 72.26)
Singapore	61.47	72.1	52.62	61.47 (52.62 to 72.1)
Dominica	61.02	71.98	50.47	61.02 (50.47 to 71.98)
Morocco	60.03	71.85	49.05	60.03 (49.05 to 71.85)
Indonesia	59.98	71.75	49.68	59.98 (49.68 to 71.75)
New Zealand	59	71.67	48.41	59 (48.41 to 71.67)
Gambia	60.11	71.54	48.99	60.11 (48.99 to 71.54)
Cabo Verde	59.54	71.34	48.94	59.54 (48.94 to 71.34)
Sudan	60.18	71.25	50.06	60.18 (50.06 to 71.25)

Lao People's Democratic Republic	60.07	70.96	49.85	60.07<br>(49.85 to 70.96)
Burkina Faso	59.97	70.47	48.35	59.97<br>(48.35 to 70.47)
Guinea	59	70.41	47.02	59<br>(47.02 to 70.41)
Belize	59.57	70.19	50.74	59.57<br>(50.74 to 70.19)
Taiwan (Province of China)	64.22	69.82	58.04	64.22<br>(58.04 to 69.82)
Congo	58.8	69.52	47.39	58.8<br>(47.39 to 69.52)
Eswatini	57.62	69.52	47.92	57.62<br>(47.92 to 69.52)
Jamaica	58.69	68.95	49.05	58.69<br>(49.05 to 68.95)
Togo	58	68.89	47.95	58<br>(47.95 to 68.89)
Botswana	55	68.28	43.88	55<br>(43.88 to 68.28)
Timor-Leste	57.46	67.5	47.97	57.46<br>(47.97 to 67.5)
Niger	55.49	67.09	44.29	55.49<br>(44.29 to 67.09)
Low SDI	57.34	66.75	49.02	57.34<br>(49.02 to 66.75)
Namibia	55.22	66.56	45.16	55.22<br>(45.16 to 66.56)
Saint Vincent and the Grenadines	56.37	66.18	46.78	56.37<br>(46.78 to 66.18)
South Africa	54.97	66.14	46.02	54.97<br>(46.02 to 66.14)
Sierra Leone	55.63	66.05	45.51	55.63<br>(45.51 to 66.05)
Cambodia	56.19	65.82	46.9	56.19<br>(46.9 to 65.82)
Trinidad and Tobago	56.42	65.7	48.16	56.42<br>(48.16 to 65.7)
Chad	55.09	65.51	44.53	55.09<br>(44.53 to 65.51)
Grenada	54.79	65.45	46.67	54.79<br>(46.67 to 65.45)
Guyana	55.32	65.12	46.44	55.32<br>(46.44 to 65.12)

Myanmar	55.62	64.88	45.94	55.62<br>(45.94 to 64.88)
Nepal	52.96	64.32	42.78	52.96<br>(42.78 to 64.32)
Mali	54.86	64.3	45.76	54.86<br>(45.76 to 64.3)
Ghana	54.14	63.85	44.18	54.14<br>(44.18 to 63.85)
Dominican Republic	52.86	63.46	44.76	52.86<br>(44.76 to 63.46)
Kenya	53	63.38	44	53<br>(44 to 63.38)
Haiti	53.61	63.31	45.01	53.61<br>(45.01 to 63.31)
Zambia	52.71	62.89	43.47	52.71<br>(43.47 to 62.89)
Angola	51.54	62.42	42.73	51.54<br>(42.73 to 62.42)
Democratic Republic of the Congo	50.94	62.29	40.69	50.94<br>(40.69 to 62.29)
Uganda	52.06	62.08	42.67	52.06<br>(42.67 to 62.08)
Comoros	52.36	61.81	43.42	52.36<br>(43.42 to 61.81)
Ethiopia	51.47	61.46	42.59	51.47<br>(42.59 to 61.46)
Central African Republic	51.45	61.31	42.06	51.45<br>(42.06 to 61.31)
Republic of Korea	50.86	61.12	41.71	50.86<br>(41.71 to 61.12)
Cuba	52.73	61.1	44.88	52.73<br>(44.88 to 61.1)
South Sudan	49.6	60.75	40.35	49.6<br>(40.35 to 60.75)
Rwanda	50.2	60.21	41.59	50.2<br>(41.59 to 60.21)
Burundi	49.54	60.15	40.74	49.54<br>(40.74 to 60.15)
Equatorial Guinea	51.5	60	42.51	51.5<br>(42.51 to 60)
Eritrea	48.91	59.59	40.78	48.91<br>(40.78 to 59.59)
Djibouti	49.26	59.31	39.65	49.26<br>(39.65 to 59.31)

Yemen	50.13	59.08	41.71	50.13<br>(41.71 to 59.08)
Malawi	49.79	58.49	41.21	49.79<br>(41.21 to 58.49)
Suriname	49.47	58.41	41.52	49.47<br>(41.52 to 58.41)
Mozambique	47.2	58.28	38.77	47.2<br>(38.77 to 58.28)
Lesotho	47.71	56.89	39.02	47.71<br>(39.02 to 56.89)
Somalia	46.22	56.43	37.89	46.22<br>(37.89 to 56.43)
Madagascar	44.79	54.9	36.5	44.79<br>(36.5 to 54.9)
United Republic of Tanzania	47.11	54.72	39.42	47.11<br>(39.42 to 54.72)
Norway	26.38	31.01	22.3	26.38<br>(22.3 to 31.01)

Table S4: ASPR of PD by country in 2021

location	val	upper	lower	Rate_2021
China	245.73	289.24	208.28	245.73<br>(208.28 to 289.24)
Israel	199.71	240.28	166.21	199.71<br>(166.21 to 240.28)
Canada	197.61	209.27	184.67	197.61<br>(184.67 to 209.27)
Taiwan (Province of China)	194.47	204.06	185.6	194.47<br>(185.6 to 204.06)
Germany	186.45	194.45	178.73	186.45<br>(178.73 to 194.45)
Spain	177.82	207.53	146.98	177.82<br>(146.98 to 207.53)
High-middle SDI	173.39	200.71	151.36	173.39<br>(151.36 to 200.71)
Iceland	172.6	207.73	140.72	172.6<br>(140.72 to 207.73)
Netherlands	168.81	192.2	148.57	168.81<br>(148.57 to 192.2)
Democratic People's Republic of Korea	161.47	191.36	132.14	161.47<br>(132.14 to 191.36)
Qatar	161.22	198.1	128.85	161.22<br>(128.85 to 198.1)
Peru	160.74	194.05	134.43	160.74<br>(134.43 to 194.05)

Bolivia (Plurinational State of)	160.33	192.73	132.7	160.33 (132.7 to 192.73)
Luxembourg	157.37	186.49	130.18	157.37 (130.18 to 186.49)
Monaco	157.08	193.96	127.95	157.08 (127.95 to 193.96)
Middle SDI	154.79	179.22	133.73	154.79 (133.73 to 179.22)
Ecuador	154.51	181.84	126.86	154.51 (126.86 to 181.84)
Ireland	153.91	176.14	128.4	153.91 (128.4 to 176.14)
Cyprus	152.47	185.55	124.46	152.47 (124.46 to 185.55)
Finland	152.37	188.12	126.25	152.37 (126.25 to 188.12)
France	152.06	177.46	118.77	152.06 (118.77 to 177.46)
Malta	148.84	175.41	126.92	148.84 (126.92 to 175.41)
Switzerland	148.82	176.94	122.58	148.82 (122.58 to 176.94)
Austria	146.48	176.85	112.37	146.48 (112.37 to 176.85)
Belgium	142.14	163.39	121.64	142.14 (121.64 to 163.39)
United Kingdom	141.1	163.16	121.2	141.1 (121.2 to 163.16)
United Arab Emirates	140.65	173.95	112.67	140.65 (112.67 to 173.95)
Andorra	140.34	164.99	118.02	140.34 (118.02 to 164.99)
Greece	137.11	165.04	116.09	137.11 (116.09 to 165.04)
San Marino	136.33	169.22	109.86	136.33 (109.86 to 169.22)
Egypt	135.75	155.89	115.05	135.75 (115.05 to 155.89)
Denmark	135.73	164.14	108.01	135.73 (108.01 to 164.14)
Oman	135.68	161.63	106.19	135.68 (106.19 to 161.63)
Saudi Arabia	133.32	162.56	109.53	133.32 (109.53 to 162.56)

High SDI	125.17	135.55	115.35	125.17 (115.35 to 135.55)
Sweden	123.14	145.94	101.86	123.14 (101.86 to 145.94)
Chile	119.69	139.52	100.48	119.69 (100.48 to 139.52)
Bahrain	116.21	139.07	97.46	116.21 (97.46 to 139.07)
Greenland	116.03	137.41	95.35	116.03 (95.35 to 137.41)
Uruguay	115.28	136.37	100.16	115.28 (100.16 to 136.37)
Honduras	114.74	137.78	93.72	114.74 (93.72 to 137.78)
Palau	114.71	146.4	93.39	114.71 (93.39 to 146.4)
Italy	113.18	132.49	93.93	113.18 (93.93 to 132.49)
Portugal	112.31	132.41	90.59	112.31 (90.59 to 132.41)
United States of America	111.85	121.45	102.83	111.85 (102.83 to 121.45)
Nicaragua	111.76	130.73	91.94	111.76 (91.94 to 130.73)
Northern Mariana Islands	109.02	126.85	91.74	109.02 (91.74 to 126.85)
Panama	108.73	127.08	90.78	108.73 (90.78 to 127.08)
Costa Rica	108.07	131.66	91.81	108.07 (91.81 to 131.66)
Seychelles	105.5	125.54	89.28	105.5 (89.28 to 125.54)
Belarus	104.37	121.46	88.36	104.37 (88.36 to 121.46)
Cook Islands	104.23	128.59	85.04	104.23 (85.04 to 128.59)
Iran (Islamic Republic of)	103.68	122.38	86.54	103.68 (86.54 to 122.38)
North Macedonia	103.48	121.51	86.96	103.48 (86.96 to 121.51)
Poland	103.34	115.62	93.22	103.34 (93.22 to 115.62)
Viet Nam	103.23	124.63	87	103.23 (87 to 124.63)

Argentina	103.07	124.5	87.56	103.07 (87.56 to 124.5)
United States Virgin Islands	102.91	122.2	88.55	102.91 (88.55 to 122.2)
Niue	102.87	125.12	87.76	102.87 (87.76 to 125.12)
Tajikistan	102.83	123.82	82.4	102.83 (82.4 to 123.82)
Estonia	102.73	119.43	87.63	102.73 (87.63 to 119.43)
Palestine	101.51	118.72	84.61	101.51 (84.61 to 118.72)
Slovenia	101.16	116.38	84.51	101.16 (84.51 to 116.38)
Mexico	100.92	118.59	85.6	100.92 (85.6 to 118.59)
Montenegro	100.84	115.95	83.69	100.84 (83.69 to 115.95)
El Salvador	99.89	114.58	84.1	99.89 (84.1 to 114.58)
Nauru	99.61	117.88	83.34	99.61 (83.34 to 117.88)
Croatia	99.47	118.07	81.05	99.47 (81.05 to 118.07)
Albania	98.96	116.15	79.1	98.96 (79.1 to 116.15)
Turkiye	98.93	119.83	81.08	98.93 (81.08 to 119.83)
Marshall Islands	98.5	120.15	80.34	98.5 (80.34 to 120.15)
Guam	98.3	123.57	84.12	98.3 (84.12 to 123.57)
Colombia	97.33	113.01	81.54	97.33 (81.54 to 113.01)
Brunei Darussalam	96.97	118.4	80.56	96.97 (80.56 to 118.4)
Bosnia and Herzegovina	96.97	118.59	78.82	96.97 (78.82 to 118.59)
Kuwait	96.39	117.02	77.51	96.39 (77.51 to 117.02)
Norway	96.32	113.97	80.36	96.32 (80.36 to 113.97)
Tunisia	96.25	113.7	78.01	96.25 (78.01 to 113.7)

Iraq	95.97	115.65	78.21	95.97<br>(78.21 to 115.65)
Morocco	95.94	113.87	78.94	95.94<br>(78.94 to 113.87)
Ukraine	95.78	113.3	79.74	95.78<br>(79.74 to 113.3)
Algeria	95.76	114.35	78.73	95.76<br>(78.73 to 114.35)
Samoa	95.54	112.59	79.97	95.54<br>(79.97 to 112.59)
Syrian Arab Republic	95.39	114.17	79.21	95.39<br>(79.21 to 114.17)
Bermuda	95.39	108.75	82.1	95.39<br>(82.1 to 108.75)
Bulgaria	95.12	111.83	79.51	95.12<br>(79.51 to 111.83)
Czechia	94.67	110.5	81.69	94.67<br>(81.69 to 110.5)
Serbia	94.15	109.98	74.69	94.15<br>(74.69 to 109.98)
India	93.9	111.05	78.33	93.9<br>(78.33 to 111.05)
Maldives	93.81	113.67	78.39	93.81<br>(78.39 to 113.67)
Bhutan	93.8	113.94	74.71	93.8<br>(74.71 to 113.94)
Kazakhstan	93.39	106.97	79.56	93.39<br>(79.56 to 106.97)
Latvia	93.38	113.2	80.77	93.38<br>(80.77 to 113.2)
Sri Lanka	93.13	113.9	75.38	93.13<br>(75.38 to 113.9)
Venezuela (Bolivarian Republic of)	93.01	107.68	80.8	93.01<br>(80.8 to 107.68)
Lebanon	92.56	111.38	77.33	92.56<br>(77.33 to 111.38)
Malaysia	92.24	114.51	76.38	92.24<br>(76.38 to 114.51)
Lithuania	91.99	106.57	77.61	91.99<br>(77.61 to 106.57)
Low-middle SDI	91.89	106.58	79.39	91.89<br>(79.39 to 106.58)
Libya	91.6	106.18	76.88	91.6<br>(76.88 to 106.18)

Vanuatu	91.01	109.91	75.9	91.01<br>(75.9 to 109.91)
Uzbekistan	90.81	104.1	76.43	90.81<br>(76.43 to 104.1)
Thailand	90.7	105.02	77.7	90.7<br>(77.7 to 105.02)
Brazil	90.59	106.45	77.08	90.59<br>(77.08 to 106.45)
Micronesia (Federated States of)	90.39	107.74	73.38	90.39<br>(73.38 to 107.74)
Russian Federation	90.27	107.45	75.14	90.27<br>(75.14 to 107.45)
Australia	89.93	108.9	76.29	89.93<br>(76.29 to 108.9)
Fiji	89.34	109.39	70.9	89.34<br>(70.9 to 109.39)
Azerbaijan	89.25	106.24	71.04	89.25<br>(71.04 to 106.24)
Pakistan	89.08	105.65	73.86	89.08<br>(73.86 to 105.65)
Mauritius	89	104.78	72.44	89<br>(72.44 to 104.78)
Sudan	88.81	107.92	72.19	88.81<br>(72.19 to 107.92)
Republic of Korea	88.74	103.16	73.9	88.74<br>(73.9 to 103.16)
Antigua and Barbuda	88.47	108.12	74.34	88.47<br>(74.34 to 108.12)
Paraguay	88.44	101.66	74.49	88.44<br>(74.49 to 101.66)
Romania	87.87	99.82	74.46	87.87<br>(74.46 to 99.82)
Tonga	86.8	103.52	73.11	86.8<br>(73.11 to 103.52)
Solomon Islands	86.37	104.19	70.12	86.37<br>(70.12 to 104.19)
Kiribati	85.45	103.22	71.4	85.45<br>(71.4 to 103.22)
Slovakia	85.31	100.92	71.73	85.31<br>(71.73 to 100.92)
Bangladesh	85.18	102.04	70.64	85.18<br>(70.64 to 102.04)
Sao Tome and Principe	84.98	102.83	70.21	84.98<br>(70.21 to 102.83)

Hungary	84.93	98.56	74.06	84.93 (74.06 to 98.56)
Guatemala	84.49	101.63	69.46	84.49 (69.46 to 101.63)
Cabo Verde	84.18	98.81	68.96	84.18 (68.96 to 98.81)
Nepal	83.38	99.79	68.53	83.38 (68.53 to 99.79)
Puerto Rico	82.74	95.41	68.25	82.74 (68.25 to 95.41)
Singapore	81.35	96.88	68.38	81.35 (68.38 to 96.88)
Barbados	80.67	95.78	68.7	80.67 (68.7 to 95.78)
Bahamas	79.9	93.13	68.58	79.9 (68.58 to 93.13)
Saint Lucia	79.88	94.04	65.7	79.88 (65.7 to 94.04)
Afghanistan	79.83	93.25	65.93	79.83 (65.93 to 93.25)
Gabon	79.82	95.74	67.43	79.82 (67.43 to 95.74)
Saint Kitts and Nevis	79.69	95.61	67.28	79.69 (67.28 to 95.61)
Indonesia	79.42	94.44	65.71	79.42 (65.71 to 94.44)
Republic of Moldova	78.97	93.66	64.58	78.97 (64.58 to 93.66)
Nigeria	78.46	93.47	64.74	78.46 (64.74 to 93.47)
Senegal	78.18	93.92	65.1	78.18 (65.1 to 93.92)
Armenia	78.04	92.97	65.04	78.04 (65.04 to 92.97)
Cameroon	77.81	93.79	64.03	77.81 (64.03 to 93.79)
Equatorial Guinea	77.75	92.87	62.38	77.75 (62.38 to 92.87)
Papua New Guinea	77.56	91.54	62.84	77.56 (62.84 to 91.54)
Ivoire	77.39	90.29	63.35	77.39 (63.35 to 90.29)
Gambia	76.05	91.98	61.72	76.05 (61.72 to 91.98)

Belize	75.7	88.72	63.92	75.7<br>(63.92 to 88.72)
Jordan	75.66	86.24	65.65	75.66<br>(65.65 to 86.24)
Myanmar	75.54	87.26	62.89	75.54<br>(62.89 to 87.26)
Dominica	75.49	87.82	63.32	75.49<br>(63.32 to 87.82)
Grenada	74.78	89.49	63.19	74.78<br>(63.19 to 89.49)
Philippines	74.67	88.47	62.35	74.67<br>(62.35 to 88.47)
Yemen	74.38	88.47	62.02	74.38<br>(62.02 to 88.47)
Lao People's Democratic Republic	74.26	89.27	62.19	74.26<br>(62.19 to 89.27)
Timor-Leste	74.2	89.79	60.88	74.2<br>(60.88 to 89.79)
Georgia	74.11	86.14	64.71	74.11<br>(64.71 to 86.14)
Cambodia	73.94	86.59	62.53	73.94<br>(62.53 to 86.59)
Cuba	73.84	84.84	64.17	73.84<br>(64.17 to 84.84)
American Samoa	73.81	86.46	62.36	73.81<br>(62.36 to 86.46)
Dominican Republic	73.62	85.84	62.34	73.62<br>(62.34 to 85.84)
Mauritania	73.3	89.7	59.75	73.3<br>(59.75 to 89.7)
Jamaica	73.21	88.59	62.13	73.21<br>(62.13 to 88.59)
Low SDI	72.87	83.78	63.07	72.87<br>(63.07 to 83.78)
Benin	71.95	86.59	58.13	71.95<br>(58.13 to 86.59)
Guinea-Bissau	70.83	86.55	58.11	70.83<br>(58.11 to 86.55)
Guinea	70.72	82.95	58.7	70.72<br>(58.7 to 82.95)
Saint Vincent and the Grenadines	70.48	83.05	59.66	70.48<br>(59.66 to 83.05)
Botswana	70.42	85.33	57.33	70.42<br>(57.33 to 85.33)

Congo	70.27	85.9	57.88	70.27<br>(57.88 to 85.9)
Zimbabwe	70	82.97	56.4	70<br>(56.4 to 82.97)
Liberia	69.61	80.55	56.57	69.61<br>(56.57 to 80.55)
Kyrgyzstan	69.52	82.4	58.08	69.52<br>(58.08 to 82.4)
Trinidad and Tobago	69.08	82.4	57.38	69.08<br>(57.38 to 82.4)
Guyana	68.98	80.73	59.47	68.98<br>(59.47 to 80.73)
New Zealand	68.81	83.28	56.87	68.81<br>(56.87 to 83.28)
Namibia	68.8	81.42	56.3	68.8<br>(56.3 to 81.42)
Burkina Faso	68.76	81.13	57.59	68.76<br>(57.59 to 81.13)
Eswatini	68.75	81.87	56.22	68.75<br>(56.22 to 81.87)
Ghana	68.5	82.57	56.71	68.5<br>(56.71 to 82.57)
South Africa	68.26	81	57.02	68.26<br>(57.02 to 81)
Togo	68.15	83.61	53.05	68.15<br>(53.05 to 83.61)
Mongolia	67.51	81.27	56.11	67.51<br>(56.11 to 81.27)
Turkmenistan	66.75	76.7	56.03	66.75<br>(56.03 to 76.7)
Sierra Leone	65.48	76.67	54.1	65.48<br>(54.1 to 76.67)
Suriname	65.3	77.34	53.56	65.3<br>(53.56 to 77.34)
Haiti	64.28	74.31	54.59	64.28<br>(54.59 to 74.31)
Chad	63.85	77.78	52.36	63.85<br>(52.36 to 77.78)
Angola	63.33	77.38	51.28	63.33<br>(51.28 to 77.38)
Mali	62.59	74.78	52.48	62.59<br>(52.48 to 74.78)
Kenya	62.55	73.85	52.25	62.55<br>(52.25 to 73.85)

Niger	61.62	75.17	50.56	61.62<br>(50.56 to 75.17)
Uganda	60.94	71.11	49.96	60.94<br>(49.96 to 71.11)
Ethiopia	60.76	71.88	50.31	60.76<br>(50.31 to 71.88)
Djibouti	60.72	71.85	50.25	60.72<br>(50.25 to 71.85)
Zambia	60.69	71.69	49.62	60.69<br>(49.62 to 71.69)
Comoros	60.55	73.9	48.34	60.55<br>(48.34 to 73.9)
Lesotho	60.17	71.89	50.25	60.17<br>(50.25 to 71.89)
United Republic of Tanzania	59.68	66.13	53.4	59.68<br>(53.4 to 66.13)
Democratic Republic of the Congo	58.88	70.34	47.74	58.88<br>(47.74 to 70.34)
Eritrea	58.22	68.55	47.9	58.22<br>(47.9 to 68.55)
Malawi	57.56	67.67	47.63	57.56<br>(47.63 to 67.67)
Rwanda	56.76	68.48	46.16	56.76<br>(46.16 to 68.48)
Central African Republic	56.25	67.67	46.61	56.25<br>(46.61 to 67.67)
Burundi	56.22	66.67	46.66	56.22<br>(46.66 to 66.67)
Mozambique	56.2	67.47	46.16	56.2<br>(46.16 to 67.47)
South Sudan	54.11	64.72	42.13	54.11<br>(42.13 to 64.72)
Madagascar	53.03	63.24	42.63	53.03<br>(42.63 to 63.24)
Japan	52.95	62.4	44.63	52.95<br>(44.63 to 62.4)
Somalia	49.02	60.17	40.07	49.02<br>(40.07 to 60.17)

Table S5: ASMR of PD by country in 1990

location	val	upper	lower	Rate_1990
Qatar	12.99	14.9	11.17	12.99<br>(11.17 to 14.9)
Cyprus	12.78	15.17	10.57	12.78<br>(10.57 to 15.17)

Nauru	8.87	11.32	6.68	8.87 (6.68 to 11.32)
Saudi Arabia	8.72	10.91	6.65	8.72 (6.65 to 10.91)
Saint Kitts and Nevis	8.68	9.07	8.24	8.68 (8.24 to 9.07)
Bahrain	8.24	9.2	7.31	8.24 (7.31 to 9.2)
United States Virgin Islands	7.91	9.79	6.16	7.91 (6.16 to 9.79)
Tajikistan	7.89	13.06	5.4	7.89 (5.4 to 13.06)
Greenland	7.83	10.53	4.85	7.83 (4.85 to 10.53)
Afghanistan	7.59	10.65	5.48	7.59 (5.48 to 10.65)
Egypt	7.55	8.9	6.67	7.55 (6.67 to 8.9)
Seychelles	7.09	8.01	6.05	7.09 (6.05 to 8.01)
Serbia	7.04	7.88	6.2	7.04 (6.2 to 7.88)
Haiti	7.02	8.65	5.02	7.02 (5.02 to 8.65)
Saint Lucia	6.82	7.11	6.47	6.82 (6.47 to 7.11)
Bolivia (Plurinational State of)	6.81	8.21	5.17	6.81 (5.17 to 8.21)
Bulgaria	6.71	7.06	6.35	6.71 (6.35 to 7.06)
Marshall Islands	6.68	7.55	5.8	6.68 (5.8 to 7.55)
Honduras	6.57	7.61	5.55	6.57 (5.55 to 7.61)
Micronesia (Federated States of)	6.56	7.68	5.5	6.56 (5.5 to 7.68)
Turkiye	6.56	7.63	5.62	6.56 (5.62 to 7.63)
Cameroon	6.53	7.71	5.5	6.53 (5.5 to 7.71)
Guinea-Bissau	6.47	7.74	5.18	6.47 (5.18 to 7.74)
Dominica	6.41	7.91	5.32	6.41 (5.32 to 7.91)

Palestine	6.33	7.53	5.26	6.33 (5.26 to 7.53)
Niue	6.31	7.22	5.44	6.31 (5.44 to 7.22)
Kuwait	6.27	6.78	5.46	6.27 (5.46 to 6.78)
Bermuda	6.21	7.13	4.92	6.21 (4.92 to 7.13)
Gabon	6.21	8.01	4.74	6.21 (4.74 to 8.01)
Vanuatu	6.19	7.64	5.01	6.19 (5.01 to 7.64)
China	6.11	6.78	5.38	6.11 (5.38 to 6.78)
Ivoire	5.98	6.93	5.08	5.98 (5.08 to 6.93)
Samoa	5.9	7.23	4.85	5.9 (4.85 to 7.23)
Maldives	5.89	6.77	4.61	5.89 (4.61 to 6.77)
Cook Islands	5.87	6.78	5	5.87 (5 to 6.78)
Albania	5.83	6.59	5.14	5.83 (5.14 to 6.59)
Croatia	5.82	6.15	5.47	5.82 (5.47 to 6.15)
Thailand	5.81	6.85	4.84	5.81 (4.84 to 6.85)
Syrian Arab Republic	5.8	7.25	4.81	5.8 (4.81 to 7.25)
Solomon Islands	5.77	6.98	4.6	5.77 (4.6 to 6.98)
United Arab Emirates	5.68	7.39	3.96	5.68 (3.96 to 7.39)
American Samoa	5.67	6.43	4.81	5.67 (4.81 to 6.43)
Iceland	5.67	6.02	5.09	5.67 (5.09 to 6.02)
Mali	5.67	6.72	4.74	5.67 (4.74 to 6.72)
North Macedonia	5.6	6.3	4.95	5.6 (4.95 to 6.3)
Fiji	5.6	6.5	4.84	5.6 (4.84 to 6.5)

Oman	5.6	7.02	4.29	5.6<br>(4.29 to 7.02)
Northern Mariana Islands	5.6	6.95	4.27	5.6<br>(4.27 to 6.95)
Palau	5.59	6.58	4.75	5.59<br>(4.75 to 6.58)
Kiribati	5.54	6.56	4.5	5.54<br>(4.5 to 6.56)
Netherlands	5.53	5.83	5.03	5.53<br>(5.03 to 5.83)
Luxembourg	5.47	5.73	5.17	5.47<br>(5.17 to 5.73)
Rwanda	5.46	7.38	3.84	5.46<br>(3.84 to 7.38)
Guatemala	5.4	5.57	5.12	5.4<br>(5.12 to 5.57)
Democratic People's Republic of Korea	5.37	6.5	4.21	5.37<br>(4.21 to 6.5)
Jordan	5.33	6.49	4.36	5.33<br>(4.36 to 6.49)
Lebanon	5.31	6.89	3.41	5.31<br>(3.41 to 6.89)
Chile	5.3	5.52	4.94	5.3<br>(4.94 to 5.52)
Congo	5.27	6.3	4.48	5.27<br>(4.48 to 6.3)
Taiwan (Province of China)	5.25	5.46	4.92	5.25<br>(4.92 to 5.46)
Bosnia and Herzegovina	5.24	5.8	4.62	5.24<br>(4.62 to 5.8)
Guam	5.22	6.22	4.15	5.22<br>(4.15 to 6.22)
Malta	5.21	5.49	4.79	5.21<br>(4.79 to 5.49)
Argentina	5.19	5.41	4.83	5.19<br>(4.83 to 5.41)
Peru	5.18	5.89	4.46	5.18<br>(4.46 to 5.89)
Monaco	5.17	6.38	3.95	5.17<br>(3.95 to 6.38)
Liberia	5.15	6.04	4.45	5.15<br>(4.45 to 6.04)
Mexico	5.14	5.29	4.87	5.14<br>(4.87 to 5.29)

Algeria	5.1	6.08	4.25	5.1<br>(4.25 to 6.08)
Burkina Faso	5.08	6.53	3.93	5.08<br>(3.93 to 6.53)
Zimbabwe	5.06	5.97	4.15	5.06<br>(4.15 to 5.97)
Lao People's Democratic Republic	5.05	6.25	4.01	5.05<br>(4.01 to 6.25)
United Kingdom	5.05	5.22	4.66	5.05<br>(4.66 to 5.22)
Israel	5.02	5.28	4.53	5.02<br>(4.53 to 5.28)
Iraq	5.02	6.16	4.1	5.02<br>(4.1 to 6.16)
Guinea	5.02	6.28	3.97	5.02<br>(3.97 to 6.28)
Papua New Guinea	5	6.64	3.82	5<br>(3.82 to 6.64)
High-middle SDI	4.99	5.27	4.56	4.99<br>(4.56 to 5.27)
Mauritania	4.99	6.09	3.91	4.99<br>(3.91 to 6.09)
Antigua and Barbuda	4.95	5.23	4.57	4.95<br>(4.57 to 5.23)
Middle SDI	4.95	5.41	4.46	4.95<br>(4.46 to 5.41)
Pakistan	4.94	6.01	3.97	4.94<br>(3.97 to 6.01)
Eswatini	4.94	6.38	3.78	4.94<br>(3.78 to 6.38)
Senegal	4.94	5.87	4.04	4.94<br>(4.04 to 5.87)
Nigeria	4.93	5.71	4.16	4.93<br>(4.16 to 5.71)
Brunei Darussalam	4.92	6.94	2.43	4.92<br>(2.43 to 6.94)
Sri Lanka	4.91	5.56	4.41	4.91<br>(4.41 to 5.56)
Sao Tome and Principe	4.89	5.4	4.32	4.89<br>(4.32 to 5.4)
Benin	4.88	5.81	4.03	4.88<br>(4.03 to 5.81)
Uruguay	4.86	5.09	4.54	4.86<br>(4.54 to 5.09)

Cambodia	4.85	5.88	3.97	4.85 (3.97 to 5.88)
Montenegro	4.84	5.74	4.1	4.84 (4.1 to 5.74)
Spain	4.82	5.05	4.38	4.82 (4.38 to 5.05)
Puerto Rico	4.81	5.02	4.5	4.81 (4.5 to 5.02)
Central African Republic	4.79	5.64	3.93	4.79 (3.93 to 5.64)
Trinidad and Tobago	4.79	4.98	4.54	4.79 (4.54 to 4.98)
Romania	4.78	4.99	4.51	4.78 (4.51 to 4.99)
Canada	4.76	4.97	4.35	4.76 (4.35 to 4.97)
Ireland	4.75	4.93	4.42	4.75 (4.42 to 4.93)
Italy	4.74	4.98	4.24	4.74 (4.24 to 4.98)
Saint Vincent and the Grenadines	4.73	4.99	4.41	4.73 (4.41 to 4.99)
Slovakia	4.72	5.28	4.25	4.72 (4.25 to 5.28)
Libya	4.71	6.31	3.38	4.71 (3.38 to 6.31)
Dominican Republic	4.65	5.59	4	4.65 (4 to 5.59)
Greece	4.62	4.85	4.25	4.62 (4.25 to 4.85)
Poland	4.62	4.8	4.32	4.62 (4.32 to 4.8)
Andorra	4.62	6.11	3.47	4.62 (3.47 to 6.11)
Bangladesh	4.61	5.89	3.65	4.61 (3.65 to 5.89)
Burundi	4.61	5.88	3.32	4.61 (3.32 to 5.88)
Low SDI	4.61	5.56	3.8	4.61 (3.8 to 5.56)
Sierra Leone	4.59	5.37	3.85	4.59 (3.85 to 5.37)
Austria	4.59	4.81	4.21	4.59 (4.21 to 4.81)

France	4.58	4.79	4.2	4.58<br>(4.2 to 4.79)
Ethiopia	4.57	6	3.59	4.57<br>(3.59 to 6)
Australia	4.57	4.79	4.17	4.57<br>(4.17 to 4.79)
Guyana	4.57	4.94	4.19	4.57<br>(4.19 to 4.94)
Latvia	4.56	4.98	4.14	4.56<br>(4.14 to 4.98)
Mauritius	4.54	4.75	4.23	4.54<br>(4.23 to 4.75)
Viet Nam	4.54	5.64	3.75	4.54<br>(3.75 to 5.64)
Equatorial Guinea	4.53	5.59	3.59	4.53<br>(3.59 to 5.59)
Belarus	4.53	5.13	3.95	4.53<br>(3.95 to 5.13)
Belgium	4.52	4.77	4.09	4.52<br>(4.09 to 4.77)
Bhutan	4.51	5.8	3.28	4.51<br>(3.28 to 5.8)
Gambia	4.49	5.69	3.34	4.49<br>(3.34 to 5.69)
Republic of Moldova	4.46	4.71	4.19	4.46<br>(4.19 to 4.71)
Republic of Korea	4.45	5.81	3.81	4.45<br>(3.81 to 5.81)
Botswana	4.44	5.55	3.53	4.44<br>(3.53 to 5.55)
Morocco	4.42	5.42	3.59	4.42<br>(3.59 to 5.42)
Niger	4.41	5.45	3.29	4.41<br>(3.29 to 5.45)
Slovenia	4.41	4.65	4.13	4.41<br>(4.13 to 4.65)
Iran (Islamic Republic of)	4.4	5.06	2.75	4.4<br>(2.75 to 5.06)
Sudan	4.37	5.85	3.49	4.37<br>(3.49 to 5.85)
Hungary	4.36	4.54	4.13	4.36<br>(4.13 to 4.54)
Azerbaijan	4.36	5.77	3.35	4.36<br>(3.35 to 5.77)

Democratic Republic of the Congo	4.36	5.39	3.39	4.36 (3.39 to 5.39)
Low-middle SDI	4.36	5.19	3.66	4.36 (3.66 to 5.19)
Comoros	4.36	5.44	3.4	4.36 (3.4 to 5.44)
Switzerland	4.36	4.62	3.93	4.36 (3.93 to 4.62)
Tonga	4.35	6.04	3.34	4.35 (3.34 to 6.04)
South Sudan	4.34	5.48	3.45	4.34 (3.45 to 5.48)
Czechia	4.33	4.5	4.11	4.33 (4.11 to 4.5)
Sweden	4.32	4.52	3.93	4.32 (3.93 to 4.52)
Barbados	4.31	4.53	4.03	4.31 (4.03 to 4.53)
Togo	4.29	5.32	3.38	4.29 (3.38 to 5.32)
Brazil	4.28	4.49	3.82	4.28 (3.82 to 4.49)
Estonia	4.28	4.64	3.94	4.28 (3.94 to 4.64)
Namibia	4.27	5.32	3.5	4.27 (3.5 to 5.32)
Myanmar	4.25	5.19	3.57	4.25 (3.57 to 5.19)
El Salvador	4.24	4.98	3.69	4.24 (3.69 to 4.98)
Finland	4.23	4.45	3.82	4.23 (3.82 to 4.45)
Yemen	4.22	5.84	3.14	4.22 (3.14 to 5.84)
High SDI	4.2	4.39	3.81	4.2 (3.81 to 4.39)
Bahamas	4.18	4.47	3.88	4.18 (3.88 to 4.47)
Eritrea	4.18	5.09	3.25	4.18 (3.25 to 5.09)
Angola	4.16	5.12	3.39	4.16 (3.39 to 5.12)
New Zealand	4.12	4.32	3.76	4.12 (3.76 to 4.32)

Germany	4.11	4.33	3.73	4.11<br>(3.73 to 4.33)
Philippines	4.07	4.59	3.6	4.07<br>(3.6 to 4.59)
Georgia	4.07	4.75	3.54	4.07<br>(3.54 to 4.75)
Nepal	4.06	5.06	3.2	4.06<br>(3.2 to 5.06)
Norway	4.06	4.25	3.67	4.06<br>(3.67 to 4.25)
Uganda	4.05	5.46	2.64	4.05<br>(2.64 to 5.46)
Cabo Verde	4.03	4.94	3.28	4.03<br>(3.28 to 4.94)
Chad	4.02	4.92	3.06	4.02<br>(3.06 to 4.92)
Indonesia	4.01	5.07	3.2	4.01<br>(3.2 to 5.07)
Ecuador	4	4.16	3.75	4<br>(3.75 to 4.16)
Portugal	4	4.16	3.71	4<br>(3.71 to 4.16)
Tunisia	4	5.23	3.17	4<br>(3.17 to 5.23)
India	3.96	5.26	2.98	3.96<br>(2.98 to 5.26)
Grenada	3.96	4.28	3.61	3.96<br>(3.61 to 4.28)
Somalia	3.93	5.14	2.76	3.93<br>(2.76 to 5.14)
Mozambique	3.92	5.34	2.92	3.92<br>(2.92 to 5.34)
Mongolia	3.91	4.75	3.33	3.91<br>(3.33 to 4.75)
United Republic of Tanzania	3.88	5.01	2.93	3.88<br>(2.93 to 5.01)
Ghana	3.87	4.57	3.2	3.87<br>(3.2 to 4.57)
Russian Federation	3.87	3.97	3.64	3.87<br>(3.64 to 3.97)
Colombia	3.86	4.03	3.57	3.86<br>(3.57 to 4.03)
Zambia	3.86	4.99	2.96	3.86<br>(2.96 to 4.99)

Suriname	3.85	4.49	3.42	3.85<br>(3.42 to 4.49)
Cuba	3.85	3.98	3.62	3.85<br>(3.62 to 3.98)
United States of America	3.82	4.03	3.36	3.82<br>(3.36 to 4.03)
Timor-Leste	3.8	4.79	2.87	3.8<br>(2.87 to 4.79)
Malaysia	3.8	4.48	3.28	3.8<br>(3.28 to 4.48)
Singapore	3.77	3.93	3.55	3.77<br>(3.55 to 3.93)
Kazakhstan	3.75	4.01	3.48	3.75<br>(3.48 to 4.01)
Malawi	3.74	4.84	2.7	3.74<br>(2.7 to 4.84)
Costa Rica	3.72	3.93	3.39	3.72<br>(3.39 to 3.93)
Armenia	3.71	4.11	3.36	3.71<br>(3.36 to 4.11)
Jamaica	3.7	3.87	3.41	3.7<br>(3.41 to 3.87)
Venezuela (Bolivarian Republic of)	3.64	3.83	3.35	3.64<br>(3.35 to 3.83)
Ukraine	3.61	3.98	3.21	3.61<br>(3.21 to 3.98)
Kyrgyzstan	3.6	4.21	3.14	3.6<br>(3.14 to 4.21)
Panama	3.58	3.78	3.26	3.58<br>(3.26 to 3.78)
Belize	3.56	3.76	3.27	3.56<br>(3.27 to 3.76)
Lithuania	3.55	3.84	3.27	3.55<br>(3.27 to 3.84)
Lesotho	3.54	4.51	2.82	3.54<br>(2.82 to 4.51)
Djibouti	3.47	4.83	2.41	3.47<br>(2.41 to 4.83)
Nicaragua	3.46	4.08	3.03	3.46<br>(3.03 to 4.08)
Madagascar	3.45	4.55	2.68	3.45<br>(2.68 to 4.55)
Denmark	3.39	3.57	3.11	3.39<br>(3.11 to 3.57)

San Marino	3.35	3.82	2.78	3.35 (2.78 to 3.82)
Kenya	3.24	4.34	2.46	3.24 (2.46 to 4.34)
Turkmenistan	3.2	3.39	2.99	3.2 (2.99 to 3.39)
Paraguay	3.17	3.68	2.68	3.17 (2.68 to 3.68)
Japan	3.16	3.32	2.83	3.16 (2.83 to 3.32)
South Africa	3	3.72	2.45	3 (2.45 to 3.72)
Uzbekistan	2.62	3.54	2.04	2.62 (2.04 to 3.54)

Table S6: ASMR of PD by country in 2021

location	val	upper	lower	Rate_2021
Honduras	9.65	11.65	7.76	9.65 (7.76 to 11.65)
Libya	6.28	10.42	4.01	6.28 (4.01 to 10.42)
Saudi Arabia	8.2	10.1	6.78	8.2 (6.78 to 10.1)
Saint Kitts and Nevis	9.09	9.93	7.98	9.09 (7.98 to 9.93)
Nauru	7.39	9.44	5.35	7.39 (5.35 to 9.44)
Afghanistan	7.19	9.19	5.44	7.19 (5.44 to 9.19)
Bolivia (Plurinational State of)	6.76	8.77	5.19	6.76 (5.19 to 8.77)
Greenland	6.76	8.64	5.12	6.76 (5.12 to 8.64)
Haiti	6.54	8.6	4.81	6.54 (4.81 to 8.6)
Guinea-Bissau	6.91	8.3	5.43	6.91 (5.43 to 8.3)
Tajikistan	6.65	8.15	5.39	6.65 (5.39 to 8.15)
Qatar	6.63	8.11	5.27	6.63 (5.27 to 8.11)
Cameroon	6.33	8.05	4.98	6.33 (4.98 to 8.05)
Montenegro	6.78	8	5.74	6.78 (5.74 to 8)

Dominica	6.59	7.98	5.38	6.59 (5.38 to 7.98)
Marshall Islands	6.48	7.9	5.29	6.48 (5.29 to 7.9)
Bahrain	6.8	7.86	5.79	6.8 (5.79 to 7.86)
Monaco	6.22	7.75	4.77	6.22 (4.77 to 7.75)
North Macedonia	6.66	7.74	5.58	6.66 (5.58 to 7.74)
Seychelles	6.46	7.7	5.1	6.46 (5.1 to 7.7)
Pakistan	6.19	7.51	5.17	6.19 (5.17 to 7.51)
Guinea	5.9	7.48	4.71	5.9 (4.71 to 7.48)
Eswatini	5.5	7.42	3.91	5.5 (3.91 to 7.42)
Syrian Arab Republic	6.09	7.42	4.85	6.09 (4.85 to 7.42)
Micronesia (Federated States of)	5.96	7.42	4.76	5.96 (4.76 to 7.42)
Senegal	5.94	7.37	4.69	5.94 (4.69 to 7.37)
Ivoire	5.99	7.33	5.01	5.99 (5.01 to 7.33)
Iraq	6.01	7.25	4.61	6.01 (4.61 to 7.25)
Gabon	5.82	7.19	4.7	5.82 (4.7 to 7.19)
Mali	5.98	7.15	4.95	5.98 (4.95 to 7.15)
Egypt	6.11	7.14	5.22	6.11 (5.22 to 7.14)
Zimbabwe	5.76	7.06	4.73	5.76 (4.73 to 7.06)
Indonesia	5.57	7.01	4.33	5.57 (4.33 to 7.01)
Oman	5.87	6.99	4.87	5.87 (4.87 to 6.99)
Mozambique	4.75	6.99	3.17	4.75 (3.17 to 6.99)
Sao Tome and Principe	5.86	6.97	4.84	5.86 (4.84 to 6.97)

Saint Lucia	6.03	6.95	5.04	6.03<br>(5.04 to 6.95)
Kiribati	5.65	6.93	4.7	5.65<br>(4.7 to 6.93)
Gambia	5.54	6.93	4.13	5.54<br>(4.13 to 6.93)
Iceland	6.23	6.91	5.17	6.23<br>(5.17 to 6.91)
Samoa	5.37	6.89	4.36	5.37<br>(4.36 to 6.89)
Solomon Islands	5.3	6.86	4.26	5.3<br>(4.26 to 6.86)
Bulgaria	6.25	6.86	5.61	6.25<br>(5.61 to 6.86)
Morocco	5.78	6.82	4.51	5.78<br>(4.51 to 6.82)
Albania	5.63	6.79	4.59	5.63<br>(4.59 to 6.79)
Mauritania	5.05	6.79	3.64	5.05<br>(3.64 to 6.79)
Liberia	5.12	6.78	3.72	5.12<br>(3.72 to 6.78)
Togo	5.13	6.78	3.88	5.13<br>(3.88 to 6.78)
Viet Nam	5.54	6.76	4.64	5.54<br>(4.64 to 6.76)
Vanuatu	5.62	6.72	4.45	5.62<br>(4.45 to 6.72)
Niue	5.84	6.68	4.92	5.84<br>(4.92 to 6.68)
Bhutan	5.65	6.65	4.74	5.65<br>(4.74 to 6.65)
Eritrea	5.16	6.65	3.84	5.16<br>(3.84 to 6.65)
Papua New Guinea	4.69	6.63	3.55	4.69<br>(3.55 to 6.63)
Nigeria	5.79	6.63	4.87	5.79<br>(4.87 to 6.63)
Burkina Faso	5.14	6.62	3.93	5.14<br>(3.93 to 6.62)
Cabo Verde	5.54	6.61	4.23	5.54<br>(4.23 to 6.61)
Equatorial Guinea	5.06	6.59	3.8	5.06<br>(3.8 to 6.59)

Bahamas	5.56	6.57	4.66	5.56 (4.66 to 6.57)
Grenada	5.99	6.56	5.32	5.99 (5.32 to 6.56)
Antigua and Barbuda	6.14	6.55	5.63	6.14 (5.63 to 6.55)
Yemen	4.7	6.54	3.36	4.7 (3.36 to 6.54)
Chad	5.17	6.54	4.12	5.17 (4.12 to 6.54)
Lao People's Democratic Republic	5.16	6.49	4.17	5.16 (4.17 to 6.49)
Rwanda	4.78	6.47	3.06	4.78 (3.06 to 6.47)
Fiji	5.39	6.43	4.42	5.39 (4.42 to 6.43)
Cambodia	5.31	6.41	4.25	5.31 (4.25 to 6.41)
Peru	5.11	6.41	4.02	5.11 (4.02 to 6.41)
Djibouti	4.19	6.37	2.92	4.19 (2.92 to 6.37)
Lesotho	4.97	6.32	3.75	4.97 (3.75 to 6.32)
Turkiye	5.27	6.31	4.37	5.27 (4.37 to 6.31)
Comoros	4.54	6.31	3.17	4.54 (3.17 to 6.31)
Bangladesh	4.53	6.31	3.29	4.53 (3.29 to 6.31)
Brunei Darussalam	4.53	6.3	2.47	4.53 (2.47 to 6.3)
Nepal	5.05	6.3	3.99	5.05 (3.99 to 6.3)
Barbados	5.31	6.29	4.38	5.31 (4.38 to 6.29)
United States of America	5.84	6.24	5.01	5.84 (5.01 to 6.24)
Myanmar	4.81	6.23	3.89	4.81 (3.89 to 6.23)
Bosnia and Herzegovina	5.12	6.22	4.18	5.12 (4.18 to 6.22)
Tonga	4.45	6.21	3.32	4.45 (3.32 to 6.21)

Thailand	4.98	6.21	3.9	4.98 (3.9 to 6.21)
Burundi	4.48	6.2	2.7	4.48 (2.7 to 6.2)
Palau	5.19	6.19	4.33	5.19 (4.33 to 6.19)
Niger	4.89	6.18	3.83	4.89 (3.83 to 6.18)
American Samoa	5.09	6.15	4.22	5.09 (4.22 to 6.15)
South Sudan	4.58	6.14	3.41	4.58 (3.41 to 6.14)
Namibia	5.08	6.13	4.15	5.08 (4.15 to 6.13)
United Arab Emirates	5.14	6.12	4.09	5.14 (4.09 to 6.12)
Democratic People's Republic of Korea	4.89	6.1	3.54	4.89 (3.54 to 6.1)
Malawi	4.28	6.1	2.76	4.28 (2.76 to 6.1)
Guyana	5.11	6.09	4.23	5.11 (4.23 to 6.09)
Congo	4.83	6.09	3.99	4.83 (3.99 to 6.09)
Benin	5.06	6.08	4.17	5.06 (4.17 to 6.08)
Saint Vincent and the Grenadines	5.54	6.07	5.01	5.54 (5.01 to 6.07)
Angola	4.65	6.04	3.57	4.65 (3.57 to 6.04)
United States Virgin Islands	4.84	6.03	3.93	4.84 (3.93 to 6.03)
Sierra Leone	4.88	5.99	3.88	4.88 (3.88 to 5.99)
Algeria	4.97	5.98	4.08	4.97 (4.08 to 5.98)
Uruguay	5.62	5.95	5.06	5.62 (5.06 to 5.95)
Low SDI	4.92	5.89	4.06	4.92 (4.06 to 5.89)
China	5.03	5.89	4.17	5.03 (4.17 to 5.89)
Northern Mariana Islands	4.96	5.81	4.08	4.96 (4.08 to 5.81)

Kazakhstan	5.24	5.8	4.67	5.24<br>(4.67 to 5.8)
Greece	5.39	5.79	4.74	5.39<br>(4.74 to 5.79)
Uganda	4.45	5.79	2.97	4.45<br>(2.97 to 5.79)
Sudan	4.34	5.78	3.34	4.34<br>(3.34 to 5.78)
Luxembourg	5.28	5.78	4.67	5.28<br>(4.67 to 5.78)
Ecuador	4.79	5.75	3.98	4.79<br>(3.98 to 5.75)
Democratic Republic of the Congo	4.24	5.75	2.82	4.24<br>(2.82 to 5.75)
Palestine	5.03	5.72	4.32	5.03<br>(4.32 to 5.72)
Paraguay	4.58	5.71	3.64	4.58<br>(3.64 to 5.71)
Ghana	4.73	5.71	3.85	4.73<br>(3.85 to 5.71)
Dominican Republic	4.52	5.71	3.59	4.52<br>(3.59 to 5.71)
Norway	5.32	5.69	4.64	5.32<br>(4.64 to 5.69)
Ethiopia	3.65	5.65	2.59	3.65<br>(2.59 to 5.65)
Kenya	4.29	5.63	3.22	4.29<br>(3.22 to 5.63)
United Republic of Tanzania	4	5.62	2.84	4<br>(2.84 to 5.62)
Central African Republic	4.54	5.61	3.36	4.54<br>(3.36 to 5.61)
Zambia	4.02	5.6	3	4.02<br>(3 to 5.6)
Belize	5.05	5.59	4.45	5.05<br>(4.45 to 5.59)
Romania	5.07	5.59	4.61	5.07<br>(4.61 to 5.59)
Netherlands	5.16	5.58	4.48	5.16<br>(4.48 to 5.58)
Malaysia	4.91	5.56	4.35	4.91<br>(4.35 to 5.56)
Belarus	4.8	5.55	4.1	4.8<br>(4.1 to 5.55)

Serbia	4.84	5.55	4.22	4.84<br>(4.22 to 5.55)
Somalia	3.88	5.54	2.38	3.88<br>(2.38 to 5.54)
Jamaica	4.54	5.54	3.67	4.54<br>(3.67 to 5.54)
Timor-Leste	4.31	5.52	3.34	4.31<br>(3.34 to 5.52)
Low-middle SDI	4.9	5.51	4.26	4.9<br>(4.26 to 5.51)
Tunisia	4.14	5.46	3.05	4.14<br>(3.05 to 5.46)
Finland	4.99	5.42	4.24	4.99<br>(4.24 to 5.42)
India	4.59	5.41	3.79	4.59<br>(3.79 to 5.41)
United Kingdom	5.11	5.4	4.51	5.11<br>(4.51 to 5.4)
Taiwan (Province of China)	4.92	5.34	4.32	4.92<br>(4.32 to 5.34)
Cyprus	4.69	5.34	4.03	4.69<br>(4.03 to 5.34)
Croatia	4.92	5.32	4.34	4.92<br>(4.34 to 5.32)
Cook Islands	4.05	5.31	3.16	4.05<br>(3.16 to 5.31)
Malta	4.81	5.31	4.08	4.81<br>(4.08 to 5.31)
High-middle SDI	4.81	5.27	4.23	4.81<br>(4.23 to 5.27)
Sri Lanka	4.12	5.27	2.98	4.12<br>(2.98 to 5.27)
El Salvador	4.42	5.26	3.62	4.42<br>(3.62 to 5.26)
Venezuela (Bolivarian Republic of)	4.31	5.26	3.49	4.31<br>(3.49 to 5.26)
Poland	4.85	5.25	4.34	4.85<br>(4.34 to 5.25)
Germany	4.88	5.25	4.18	4.88<br>(4.18 to 5.25)
New Zealand	4.84	5.22	4.21	4.84<br>(4.21 to 5.22)
Middle SDI	4.71	5.2	4.16	4.71<br>(4.16 to 5.2)

Canada	4.85	5.18	4.26	4.85<br>(4.26 to 5.18)
Slovenia	4.63	5.17	3.96	4.63<br>(3.96 to 5.17)
Czechia	4.62	5.09	4.03	4.62<br>(4.03 to 5.09)
Spain	4.7	5.09	4.04	4.7<br>(4.04 to 5.09)
Philippines	4.31	5.08	3.71	4.31<br>(3.71 to 5.08)
High SDI	4.75	5.08	4.12	4.75<br>(4.12 to 5.08)
Austria	4.71	5.05	4.04	4.71<br>(4.04 to 5.05)
Chile	4.77	5.05	4.25	4.77<br>(4.25 to 5.05)
Trinidad and Tobago	4.16	5.04	3.25	4.16<br>(3.25 to 5.04)
Cuba	4.5	5.03	3.95	4.5<br>(3.95 to 5.03)
Argentina	4.71	5	4.27	4.71<br>(4.27 to 5)
Australia	4.64	4.99	4.02	4.64<br>(4.02 to 4.99)
Denmark	4.63	4.96	4.07	4.63<br>(4.07 to 4.96)
Botswana	4.02	4.95	3.36	4.02<br>(3.36 to 4.95)
Israel	4.56	4.94	3.89	4.56<br>(3.89 to 4.94)
Andorra	3.78	4.93	2.67	3.78<br>(2.67 to 4.93)
Italy	4.55	4.92	3.87	4.55<br>(3.87 to 4.92)
Maldives	4.1	4.86	3.3	4.1<br>(3.3 to 4.86)
Bermuda	3.91	4.86	3.17	3.91<br>(3.17 to 4.86)
Belgium	4.45	4.85	3.79	4.45<br>(3.79 to 4.85)
Ireland	4.44	4.83	3.8	4.44<br>(3.8 to 4.83)
Mexico	4.36	4.83	3.9	4.36<br>(3.9 to 4.83)

Suriname	3.84	4.82	2.9	3.84<br>(2.9 to 4.82)
Estonia	4.32	4.82	3.72	4.32<br>(3.72 to 4.82)
Latvia	4.37	4.81	3.86	4.37<br>(3.86 to 4.81)
France	4.43	4.8	3.82	4.43<br>(3.82 to 4.8)
Panama	4.06	4.8	3.17	4.06<br>(3.17 to 4.8)
Jordan	3.99	4.8	3.23	3.99<br>(3.23 to 4.8)
Sweden	4.32	4.76	3.72	4.32<br>(3.72 to 4.76)
Madagascar	3.3	4.74	2.3	3.3<br>(2.3 to 4.74)
Lebanon	3.96	4.71	3.37	3.96<br>(3.37 to 4.71)
Ukraine	3.86	4.71	3.04	3.86<br>(3.04 to 4.71)
Lithuania	4.21	4.68	3.74	4.21<br>(3.74 to 4.68)
Hungary	4.25	4.65	3.78	4.25<br>(3.78 to 4.65)
Puerto Rico	4.02	4.63	3.32	4.02<br>(3.32 to 4.63)
Georgia	4.17	4.63	3.7	4.17<br>(3.7 to 4.63)
Republic of Korea	3.93	4.63	3.04	3.93<br>(3.04 to 4.63)
Azerbaijan	3.86	4.63	3.18	3.86<br>(3.18 to 4.63)
Russian Federation	4.3	4.61	3.93	4.3<br>(3.93 to 4.61)
South Africa	4.24	4.6	3.78	4.24<br>(3.78 to 4.6)
Switzerland	4.22	4.58	3.57	4.22<br>(3.57 to 4.58)
Iran (Islamic Republic of)	4.08	4.56	2.23	4.08<br>(2.23 to 4.56)
Kuwait	3.78	4.56	3.08	3.78<br>(3.08 to 4.56)
Slovakia	4	4.54	3.44	4<br>(3.44 to 4.54)

Brazil	4.24	4.54	3.71	4.24<br>(3.71 to 4.54)
Mauritius	4.24	4.51	3.83	4.24<br>(3.83 to 4.51)
Colombia	3.83	4.46	3.22	3.83<br>(3.22 to 4.46)
Portugal	4.11	4.41	3.56	4.11<br>(3.56 to 4.41)
Turkmenistan	3.54	4.28	2.83	3.54<br>(2.83 to 4.28)
Costa Rica	3.82	4.25	3.3	3.82<br>(3.3 to 4.25)
Mongolia	3.58	4.24	2.99	3.58<br>(2.99 to 4.24)
Armenia	3.81	4.23	3.36	3.81<br>(3.36 to 4.23)
Guatemala	3.64	4.09	3.18	3.64<br>(3.18 to 4.09)
Nicaragua	3.25	3.83	2.69	3.25<br>(2.69 to 3.83)
Japan	3.52	3.83	2.91	3.52<br>(2.91 to 3.83)
Uzbekistan	3.09	3.5	2.7	3.09<br>(2.7 to 3.5)
Kyrgyzstan	3.02	3.44	2.58	3.02<br>(2.58 to 3.44)
Singapore	3.05	3.27	2.67	3.05<br>(2.67 to 3.27)
Republic of Moldova	2.85	3.14	2.57	2.85<br>(2.57 to 3.14)
Guam	1.99	2.43	1.58	1.99<br>(1.58 to 2.43)
San Marino	1.72	2.35	1.16	1.72<br>(1.16 to 2.35)

Table S7: ASDR of PD by country in 1990

location	val	upper	lower	Rate_1990
Qatar	199.42	227.31	173.24	199.42<br>(173.24 to 227.31)
Cyprus	179.63	205.21	150.92	179.63<br>(150.92 to 205.21)
Nauru	157.22	198.9	120.73	157.22<br>(120.73 to 198.9)
Saudi Arabia	141.09	174.61	110.57	141.09<br>(110.57 to 174.61)

Saint Kitts and Nevis	137.08	143.88	129.87	137.08<br>(129.87 to 143.88)
Afghanistan	133.6	184.28	92.56	133.6<br>(92.56 to 184.28)
Greenland	132.4	174.56	87.25	132.4<br>(87.25 to 174.56)
Bahrain	129.91	143.6	116.09	129.91<br>(116.09 to 143.6)
United States Virgin Islands	127.7	155.27	102.73	127.7<br>(102.73 to 155.27)
Tajikistan	123.32	184.66	91.16	123.32<br>(91.16 to 184.66)
Egypt	121.89	137.19	109.13	121.89<br>(109.13 to 137.19)
Marshall Islands	121.76	137.75	105.84	121.76<br>(105.84 to 137.75)
Seychelles	121.5	135.2	104.98	121.5<br>(104.98 to 135.2)
Haiti	118.12	145.82	83.03	118.12<br>(83.03 to 145.82)
Micronesia (Federated States of)	116.81	135.37	98.25	116.81<br>(98.25 to 135.37)
Bolivia (Plurinational State of)	116.2	139.65	89.97	116.2<br>(89.97 to 139.65)
Niue	112.04	127.29	96.46	112.04<br>(96.46 to 127.29)
Vanuatu	111.85	135.72	91.45	111.85<br>(91.45 to 135.72)
Serbia	110.09	121.66	98.17	110.09<br>(98.17 to 121.66)
Bulgaria	109.56	117.03	102.17	109.56<br>(102.17 to 117.03)
Honduras	108.85	123.92	94.14	108.85<br>(94.14 to 123.92)
Guinea-Bissau	107.21	128.02	84.91	107.21<br>(84.91 to 128.02)
Gabon	105.4	132.79	81.47	105.4<br>(81.47 to 132.79)
China	105.26	116.71	93.23	105.26<br>(93.23 to 116.71)
Kuwait	105.16	114.17	94.27	105.16<br>(94.27 to 114.17)
Cook Islands	104.14	118.74	89.89	104.14<br>(89.89 to 118.74)

Saint Lucia	103.93	109.25	98.95	103.93<br>(98.95 to 109.25)
Solomon Islands	103.76	125.74	80.56	103.76<br>(80.56 to 125.74)
Cameroon	103.19	120.11	88.17	103.19<br>(88.17 to 120.11)
United Arab Emirates	103.15	132.79	74.98	103.15<br>(74.98 to 132.79)
Dominica	103.11	124.96	86.95	103.11<br>(86.95 to 124.96)
Samoa	103.03	127.08	85.14	103.03<br>(85.14 to 127.08)
Palau	101.98	118.34	88.28	101.98<br>(88.28 to 118.34)
Turkiye	101.88	115.61	88.33	101.88<br>(88.33 to 115.61)
Bermuda	101.44	114.91	80.69	101.44<br>(80.69 to 114.91)
Netherlands	101.15	109.46	91.98	101.15<br>(91.98 to 109.46)
Kiribati	101.05	115.84	85.28	101.05<br>(85.28 to 115.84)
Northern Mariana Islands	100.87	121.63	80.14	100.87<br>(80.14 to 121.63)
Iceland	100.82	108.62	91.64	100.82<br>(91.64 to 108.62)
Oman	100.54	124.39	80.01	100.54<br>(80.01 to 124.39)
Fiji	100.2	114.61	88.47	100.2<br>(88.47 to 114.61)
Maldives	99.26	113.66	80.15	99.26<br>(80.15 to 113.66)
Palestine	98.97	117.1	83.97	98.97<br>(83.97 to 117.1)
American Samoa	98.05	109.58	84.8	98.05<br>(84.8 to 109.58)
Democratic People's Republic of Korea	97.73	118.01	77.9	97.73<br>(77.9 to 118.01)
Albania	96.47	107.63	86	96.47<br>(86 to 107.63)
Bosnia and Herzegovina	96.4	106.33	86.15	96.4<br>(86.15 to 106.33)
Croatia	96.32	102.49	90.07	96.32<br>(90.07 to 102.49)

North Macedonia	96.27	107.05	86.52	96.27<br>(86.52 to 107.05)
Thailand	95.97	110.23	82.11	95.97<br>(82.11 to 110.23)
Luxembourg	95.82	102.73	89.43	95.82<br>(89.43 to 102.73)
Ivoire	95.48	109.84	81.69	95.48<br>(81.69 to 109.84)
Israel	93.36	101.84	84.97	93.36<br>(84.97 to 101.84)
Guam	92.84	107.12	75.85	92.84<br>(75.85 to 107.12)
Rwanda	92.77	125.29	69	92.77<br>(69 to 125.29)
United Kingdom	92.61	99.06	85.99	92.61<br>(85.99 to 99.06)
Syrian Arab Republic	92.36	111.08	77.95	92.36<br>(77.95 to 111.08)
Papua New Guinea	91.79	119.76	71.44	91.79<br>(71.44 to 119.76)
Malta	91.56	98.26	84.09	91.56<br>(84.09 to 98.26)
Congo	90.98	109.57	76.61	90.98<br>(76.61 to 109.57)
Monaco	90.66	109.47	72.48	90.66<br>(72.48 to 109.47)
Italy	90.15	98.5	81.45	90.15<br>(81.45 to 98.5)
Taiwan (Province of China)	90.1	94.3	84.98	90.1<br>(84.98 to 94.3)
Peru	89.59	100.87	79.12	89.59<br>(79.12 to 100.87)
Mali	89.35	104.16	75.66	89.35<br>(75.66 to 104.16)
Argentina	89.33	94.82	83.27	89.33<br>(83.27 to 94.82)
Lao People's Democratic Republic	89.05	110.41	71.59	89.05<br>(71.59 to 110.41)
High-middle SDI	88.94	94.95	82.06	88.94<br>(82.06 to 94.95)
Chile	88.52	93.25	82.62	88.52<br>(82.62 to 93.25)
Brunei Darussalam	88.31	119.19	51.81	88.31<br>(51.81 to 119.19)

Lebanon	87.94	113.31	58.91	87.94<br>(58.91 to 113.31)
Spain	87.36	94.12	79.67	87.36<br>(79.67 to 94.12)
Jordan	87.13	104.75	72.63	87.13<br>(72.63 to 104.75)
Iraq	86.99	105.05	72.98	86.99<br>(72.98 to 105.05)
Middle SDI	85.83	93.47	78.01	85.83<br>(78.01 to 93.47)
Canada	85.79	92.19	79.4	85.79<br>(79.4 to 92.19)
Uruguay	85.26	89.79	80.04	85.26<br>(80.04 to 89.79)
Montenegro	84.72	97.91	73.21	84.72<br>(73.21 to 97.91)
Central African Republic	84.59	99.12	69.58	84.59<br>(69.58 to 99.12)
Cambodia	84.3	99.48	71.27	84.3<br>(71.27 to 99.48)
Guatemala	84.07	87.93	80.11	84.07<br>(80.11 to 87.93)
Antigua and Barbuda	83.23	89.06	77.56	83.23<br>(77.56 to 89.06)
Ireland	83.22	89	77.07	83.22<br>(77.07 to 89)
Zimbabwe	82.84	95.91	68.68	82.84<br>(68.68 to 95.91)
Eswatini	82.82	104.6	64.55	82.82<br>(64.55 to 104.6)
Slovakia	82.77	91.78	75.28	82.77<br>(75.28 to 91.78)
Pakistan	82.58	96.84	69.7	82.58<br>(69.7 to 96.84)
Andorra	82.03	104.96	64.05	82.03<br>(64.05 to 104.96)
Liberia	81.92	95.23	70.3	81.92<br>(70.3 to 95.23)
Burkina Faso	81.53	103.84	64.35	81.53<br>(64.35 to 103.84)
Sri Lanka	81.23	90.42	73.75	81.23<br>(73.75 to 90.42)
Romania	81.2	85.86	76.58	81.2<br>(76.58 to 85.86)

Ethiopia	80.97	104.68	66.97	80.97 (66.97 to 104.68)
Austria	80.84	86.78	75.13	80.84 (75.13 to 86.78)
Mexico	80.79	84.69	76.85	80.79 (76.85 to 84.69)
Poland	80.71	85.38	75.92	80.71 (75.92 to 85.38)
Guinea	80.55	98.42	64.9	80.55 (64.9 to 98.42)
Guyana	80.23	87.03	73.64	80.23 (73.64 to 87.03)
Libya	80.12	103.01	61.23	80.12 (61.23 to 103.01)
Mauritania	80.11	97.64	63.36	80.11 (63.36 to 97.64)
Belarus	80.06	90.66	70.42	80.06 (70.42 to 90.66)
Algeria	80.03	93.7	67.24	80.03 (67.24 to 93.7)
Slovenia	80.03	85.82	74.57	80.03 (74.57 to 85.82)
Latvia	79.98	87.58	72.27	79.98 (72.27 to 87.58)
Burundi	79.85	100.48	60.39	79.85 (60.39 to 100.48)
Equatorial Guinea	79.75	95.76	64.28	79.75 (64.28 to 95.76)
Senegal	79.74	94.08	66.3	79.74 (66.3 to 94.08)
Bangladesh	79.7	100.73	65.54	79.7 (65.54 to 100.73)
Greece	79.67	85.72	73.47	79.67 (73.47 to 85.72)
France	79.31	85.02	73.34	79.31 (73.34 to 85.02)
Belgium	79.16	84.94	73.03	79.16 (73.03 to 84.94)
Mauritius	79.13	83.37	74.37	79.13 (74.37 to 83.37)
Tonga	78.96	106.56	62.83	78.96 (62.83 to 106.56)
Low SDI	78.87	94.4	66.71	78.87 (66.71 to 94.4)

Benin	78.84	91.89	66.73	78.84 (66.73 to 91.89)
Nigeria	78.78	91.4	67.27	78.78 (67.27 to 91.4)
Viet Nam	78.73	94.86	66.21	78.73 (66.21 to 94.86)
Switzerland	78.66	85.12	71.43	78.66 (71.43 to 85.12)
Bhutan	78.55	98.84	58.63	78.55 (58.63 to 98.84)
Estonia	78.44	86.42	71.15	78.44 (71.15 to 86.42)
Sao Tome and Principe	78.02	86.3	69.11	78.02 (69.11 to 86.3)
Republic of Moldova	77.66	83.81	71.88	77.66 (71.88 to 83.81)
Sudan	77.63	99.2	63.78	77.63 (63.78 to 99.2)
Puerto Rico	77.46	81.5	73.21	77.46 (73.21 to 81.5)
Trinidad and Tobago	77.28	80.98	73.39	77.28 (73.39 to 80.98)
Czechia	77.01	81.63	72.5	77.01 (72.5 to 81.63)
Finland	76.83	82.35	70.99	76.83 (70.99 to 82.35)
Morocco	76.76	90.87	63.79	76.76 (63.79 to 90.87)
Saint Vincent and the Grenadines	76.7	81.41	71.35	76.7 (71.35 to 81.41)
Bahamas	76.52	82.38	70.75	76.52 (70.75 to 82.38)
Iran (Islamic Republic of)	76.36	86.97	49.77	76.36 (49.77 to 86.97)
Australia	76.06	81.03	70.74	76.06 (70.74 to 81.03)
Low-middle SDI	75.94	88.95	65.7	75.94 (65.7 to 88.95)
Hungary	75.94	80.33	71.78	75.94 (71.78 to 80.33)
Myanmar	75.41	90.4	63.19	75.41 (63.19 to 90.4)
Comoros	74.93	91.62	60.54	74.93 (60.54 to 91.62)

South Sudan	74.88	92.31	61.06	74.88<br>(61.06 to 92.31)
Democratic Republic of the Congo	74.76	89.8	59.93	74.76<br>(59.93 to 89.8)
Yemen	74.7	99.23	57.23	74.7<br>(57.23 to 99.23)
High SDI	74.27	79.07	68.79	74.27<br>(68.79 to 79.07)
Eritrea	74.09	88.94	61.4	74.09<br>(61.4 to 88.94)
Georgia	73.79	83.95	65.34	73.79<br>(65.34 to 83.95)
El Salvador	73.74	83.61	66.43	73.74<br>(66.43 to 83.61)
Republic of Korea	73.67	94.34	65.23	73.67<br>(65.23 to 94.34)
Sierra Leone	73.56	86.66	62.21	73.56<br>(62.21 to 86.66)
Azerbaijan	73.49	90.65	59.83	73.49<br>(59.83 to 90.65)
Angola	73.24	88.48	61.71	73.24<br>(61.71 to 88.48)
Sweden	73.21	77.86	67.59	73.21<br>(67.59 to 77.86)
Gambia	73.05	91.72	56.35	73.05<br>(56.35 to 91.72)
Botswana	72.93	88.91	58.27	72.93<br>(58.27 to 88.91)
Germany	72.92	77.83	67.71	72.92<br>(67.71 to 77.83)
Dominican Republic	72.22	85.22	62.36	72.22<br>(62.36 to 85.22)
Niger	72.21	88.12	56.34	72.21<br>(56.34 to 88.12)
Namibia	72.05	86.43	60.46	72.05<br>(60.46 to 86.43)
Brazil	71.83	76.35	66.4	71.83<br>(66.4 to 76.35)
Barbados	71.48	76	66.71	71.48<br>(66.71 to 76)
India	70.85	90.98	56.08	70.85<br>(56.08 to 90.98)
Indonesia	70.73	85.15	58.82	70.73<br>(58.82 to 85.15)

Russian Federation	70.58	75.23	66.35	70.58<br>(66.35 to 75.23)
Malaysia	70.13	79.04	62.21	70.13<br>(62.21 to 79.04)
Mongolia	69.59	80.72	60.4	69.59<br>(60.4 to 80.72)
Togo	69.59	84.96	55.64	69.59<br>(55.64 to 84.96)
Ecuador	69.47	74.56	64.33	69.47<br>(64.33 to 74.56)
New Zealand	69.38	73.72	64.38	69.38<br>(64.38 to 73.72)
Uganda	69.37	92.67	49.46	69.37<br>(49.46 to 92.67)
Nepal	69.03	84.42	56.46	69.03<br>(56.46 to 84.42)
Somalia	68.97	88.27	51.03	68.97<br>(51.03 to 88.27)
Ukraine	68.87	77.43	61.04	68.87<br>(61.04 to 77.43)
Kazakhstan	68.6	74.33	63.65	68.6<br>(63.65 to 74.33)
Grenada	68.33	74.03	62.5	68.33<br>(62.5 to 74.03)
Tunisia	68.07	84.87	56.08	68.07<br>(56.08 to 84.87)
United States of America	68.05	73.13	62.2	68.05<br>(62.2 to 73.13)
Singapore	67.9	71.55	63.88	67.9<br>(63.88 to 71.55)
Portugal	67.48	72.25	62.63	67.48<br>(62.63 to 72.25)
Philippines	67.37	75.98	59.47	67.37<br>(59.47 to 75.98)
Colombia	67.22	71.11	63.1	67.22<br>(63.1 to 71.11)
Zambia	67.1	82.9	53.75	67.1<br>(53.75 to 82.9)
Mozambique	66.12	85.6	51.52	66.12<br>(51.52 to 85.6)
Costa Rica	66.12	71.35	60.71	66.12<br>(60.71 to 71.35)
Timor-Leste	66.11	81.13	52.4	66.11<br>(52.4 to 81.13)

Suriname	66.11	73.58	59.65	66.11 (59.65 to 73.58)
Chad	65.99	79.91	53.19	65.99 (53.19 to 79.91)
United Republic of Tanzania	65.98	81.99	51.81	65.98 (51.81 to 81.99)
Armenia	65.93	72.71	59.91	65.93 (59.91 to 72.71)
Cabo Verde	65.16	77.78	54.36	65.16 (54.36 to 77.78)
Lithuania	64.75	71.17	58.86	64.75 (58.86 to 71.17)
Malawi	64.69	79.75	49.61	64.69 (49.61 to 79.75)
Venezuela (Bolivarian Republic of)	64.08	68.24	59.28	64.08 (59.28 to 68.24)
Kyrgyzstan	63.96	72.68	56.12	63.96 (56.12 to 72.68)
Norway	63.95	66.83	59.33	63.95 (59.33 to 66.83)
Ghana	63.69	74.83	53.8	63.69 (53.8 to 74.83)
Panama	63.46	68.2	58.71	63.46 (58.71 to 68.2)
Cuba	63.35	66.72	59.57	63.35 (59.57 to 66.72)
San Marino	63.13	71.83	54.38	63.13 (54.38 to 71.83)
Belize	62.8	67.52	58.32	62.8 (58.32 to 67.52)
Jamaica	62.77	66.6	58.42	62.77 (58.42 to 66.6)
Nicaragua	61.6	70.39	54.96	61.6 (54.96 to 70.39)
Denmark	61.35	66.2	56.98	61.35 (56.98 to 66.2)
Djibouti	59.98	80.22	43.6	59.98 (43.6 to 80.22)
Madagascar	59.88	74.06	49.05	59.88 (49.05 to 74.06)
Lesotho	59.71	73.24	49.03	59.71 (49.03 to 73.24)
Turkmenistan	58.3	63.14	54.04	58.3 (54.04 to 63.14)

Kenya	55.82	71.31	44.43	55.82 (44.43 to 71.31)
Paraguay	55.74	63.99	48.14	55.74 (48.14 to 63.99)
Japan	55.56	59.42	50.87	55.56 (50.87 to 59.42)
Uzbekistan	52.12	65.57	42.11	52.12 (42.11 to 65.57)
South Africa	50.05	60.79	41.83	50.05 (41.83 to 60.79)

Table S8: ASDR of PD by country in 2021

location	val	upper	lower	Rate_2021
Honduras	157.67	188.02	131.01	157.67 (131.01 to 188.02)
Saint Kitts and Nevis	143.73	159.25	125.16	143.73 (125.16 to 159.25)
Saudi Arabia	138.08	165.19	116.74	138.08 (116.74 to 165.19)
Nauru	132.58	168.86	99.42	132.58 (99.42 to 168.86)
Afghanistan	126.51	159.77	94.53	126.51 (94.53 to 159.77)
Bolivia (Plurinational State of)	120.78	152.85	95.43	120.78 (95.43 to 152.85)
Marshall Islands	116.75	142.42	95.37	116.75 (95.37 to 142.42)
Greenland	116.37	142.87	90.96	116.37 (90.96 to 142.87)
Qatar	115.82	141.07	94.76	115.82 (94.76 to 141.07)
Guinea-Bissau	111.32	131.01	87.92	111.32 (87.92 to 131.01)
Seychelles	110.54	129.22	90.89	110.54 (90.89 to 129.22)
Tajikistan	109.6	129.51	92.1	109.6 (92.1 to 129.51)
Iceland	109.41	121.75	95.68	109.41 (95.68 to 121.75)
Haiti	109.19	140.97	81.21	109.19 (81.21 to 140.97)
Monaco	108.74	129.19	87.36	108.74 (87.36 to 129.19)
Libya	108.34	170.84	73.78	108.34 (73.78 to 170.84)

Dominica	108.09	128.81	90.04	108.09<br>(90.04 to 128.81)
China	107.96	125.52	91.1	107.96<br>(91.1 to 125.52)
Bahrain	107.57	124.67	93.12	107.57<br>(93.12 to 124.67)
Montenegro	107.06	124.53	93.44	107.06<br>(93.44 to 124.53)
Egypt	106.47	123.45	92.78	106.47<br>(92.78 to 123.45)
Micronesia (Federated States of)	106.16	130.3	86.03	106.16<br>(86.03 to 130.3)
North Macedonia	105.22	121.32	89.52	105.22<br>(89.52 to 121.32)
Niue	103.73	118.03	88.56	103.73<br>(88.56 to 118.03)
Democratic People's Republic of Korea	103.16	123.42	80.72	103.16<br>(80.72 to 123.42)
Bulgaria	103.13	113.44	92.57	103.13<br>(92.57 to 113.44)
Pakistan	103.02	122.14	87.36	103.02<br>(87.36 to 122.14)
Oman	102.33	119.24	86.16	102.33<br>(86.16 to 119.24)
Vanuatu	102.2	120.89	83.03	102.2<br>(83.03 to 120.89)
Cameroon	101.82	128.87	80.97	101.82<br>(80.97 to 128.87)
Morocco	101.46	118.67	80.79	101.46<br>(80.79 to 118.67)
Antigua and Barbuda	101.33	108.43	92.38	101.33<br>(92.38 to 108.43)
Grenada	101.2	111.03	90.95	101.2<br>(90.95 to 111.03)
Iraq	100.59	117.99	80.61	100.59<br>(80.61 to 117.99)
Kiribati	100.16	120.82	84.48	100.16<br>(84.48 to 120.82)
Taiwan (Province of China)	98.99	108.57	88.36	98.99<br>(88.36 to 108.57)
Gabon	98.62	119.27	81.48	98.62<br>(81.48 to 119.27)
United States of America	97.67	104.7	87.38	97.67<br>(87.38 to 104.7)

Uruguay	97.59	104.79	90.01	97.59<br>(90.01 to 104.79)
Peru	97.23	117.53	79.86	97.23<br>(79.86 to 117.53)
Syrian Arab Republic	97.1	118.88	79.38	97.1<br>(79.38 to 118.88)
Zimbabwe	96.85	117.42	79.51	96.85<br>(79.51 to 117.42)
Ivoire	96.48	118.18	80.67	96.48<br>(80.67 to 118.18)
Viet Nam	96.09	112.83	81.43	96.09<br>(81.43 to 112.83)
Saint Lucia	95.92	110.32	81.33	95.92<br>(81.33 to 110.32)
Canada	95.86	104.71	85.19	95.86<br>(85.19 to 104.71)
Eswatini	95.64	127.63	69.27	95.64<br>(69.27 to 127.63)
Solomon Islands	95.39	118.06	77.35	95.39<br>(77.35 to 118.06)
Greece	95.02	104.6	86.15	95.02<br>(86.15 to 104.6)
Sao Tome and Principe	94.99	109.54	80.14	94.99<br>(80.14 to 109.54)
Germany	94.95	104.23	84.23	94.95<br>(84.23 to 104.23)
United Arab Emirates	94.92	111.52	78.82	94.92<br>(78.82 to 111.52)
Senegal	94.91	114.88	76.34	94.91<br>(76.34 to 114.88)
Samoa	94.89	120.01	79.08	94.89<br>(79.08 to 120.01)
Fiji	94.88	112.75	78.57	94.88<br>(78.57 to 112.75)
Bhutan	94.86	109.92	79.93	94.86<br>(79.93 to 109.92)
Bahamas	94.53	111.38	80.77	94.53<br>(80.77 to 111.38)
Luxembourg	94.4	104.9	84.21	94.4<br>(84.21 to 104.9)
Guinea	94.34	117	75.43	94.34<br>(75.43 to 117)
Netherlands	94.29	103.13	84.28	94.29<br>(84.28 to 103.13)

High-middle SDI	94.16	104.78	84.06	94.16<br>(84.06 to 104.78)
Mali	93.83	110.88	77.67	93.83<br>(77.67 to 110.88)
Palau	93.27	110.87	78.44	93.27<br>(78.44 to 110.87)
Indonesia	93.21	113.79	74.95	93.21<br>(74.95 to 113.79)
Finland	92.47	101.79	82.15	92.47<br>(82.15 to 101.79)
United Kingdom	92.01	99.76	83.89	92.01<br>(83.89 to 99.76)
Middle SDI	91.07	100.96	80.79	91.07<br>(80.79 to 100.96)
Northern Mariana Islands	90.85	104.49	76.92	90.85<br>(76.92 to 104.49)
Spain	90.67	100.55	79.96	90.67<br>(79.96 to 100.55)
Israel	90.6	102	79.74	90.6<br>(79.74 to 102)
Nigeria	90.34	103.95	75.8	90.34<br>(75.8 to 103.95)
Albania	90.23	106.1	75.7	90.23<br>(75.7 to 106.1)
Gambia	89.75	109.53	68.04	89.75<br>(68.04 to 109.53)
Cambodia	89.74	108.2	72.08	89.74<br>(72.08 to 108.2)
Norway	89.47	96.52	80.77	89.47<br>(80.77 to 96.52)
Bosnia and Herzegovina	89.29	106.1	73.34	89.29<br>(73.34 to 106.1)
Guyana	89.04	106.8	74.38	89.04<br>(74.38 to 106.8)
Kazakhstan	89.02	97.7	80.08	89.02<br>(80.08 to 97.7)
Cabo Verde	88.91	104.39	70	88.91<br>(70 to 104.39)
Malta	88.57	99.08	78.57	88.57<br>(78.57 to 99.08)
Saint Vincent and the Grenadines	88.55	97.69	80.31	88.55<br>(80.31 to 97.69)
Lao People's Democratic Republic	88.44	109.38	71.31	88.44<br>(71.31 to 109.38)

Ecuador	88.26	104.09	75.12	88.26<br>(75.12 to 104.09)
American Samoa	88.14	106.08	74.85	88.14<br>(74.85 to 106.08)
Romania	87.21	95.23	79.36	87.21<br>(79.36 to 95.23)
Turkiye	86.75	100.68	73.01	86.75<br>(73.01 to 100.68)
Papua New Guinea	86.4	116.91	66.38	86.4<br>(66.38 to 116.91)
Thailand	86.35	106.25	70.57	86.35<br>(70.57 to 106.25)
Equatorial Guinea	86.33	111.91	66.49	86.33<br>(66.49 to 111.91)
Eritrea	86.32	106.76	66.7	86.32<br>(66.7 to 106.76)
Belize	86.23	95.7	76.59	86.23<br>(76.59 to 95.7)
Low-middle SDI	86.15	95.69	76.86	86.15<br>(76.86 to 95.69)
Barbados	86.14	100.44	72.23	86.14<br>(72.23 to 100.44)
Austria	85.71	94.19	77.36	85.71<br>(77.36 to 94.19)
Malaysia	85.64	95.91	76.95	85.64<br>(76.95 to 95.91)
High SDI	85.43	92.02	77.18	85.43<br>(77.18 to 92.02)
Lesotho	85.43	105.86	65.42	85.43<br>(65.42 to 105.86)
Namibia	85.37	101.68	70.52	85.37<br>(70.52 to 101.68)
Nepal	85.36	103.49	69.42	85.36<br>(69.42 to 103.49)
Poland	84.91	93.04	76.58	84.91<br>(76.58 to 93.04)
Belarus	84.7	96.61	73.25	84.7<br>(73.25 to 96.61)
Chad	84.19	104.28	67.44	84.19<br>(67.44 to 104.28)
Palestine	84.08	93.93	73.33	84.08<br>(73.33 to 93.93)
Chile	83.87	90.21	76.05	83.87<br>(76.05 to 90.21)

Cyprus	83.71	94.85	73	83.71<br>(73 to 94.85)
Denmark	83.68	92.05	75.4	83.68<br>(75.4 to 92.05)
Myanmar	83.67	104.62	68.7	83.67<br>(68.7 to 104.62)
Croatia	83.64	91.37	75.63	83.64<br>(75.63 to 91.37)
Belgium	83.62	91.87	74.19	83.62<br>(74.19 to 91.87)
Serbia	83.4	92.92	73.46	83.4<br>(73.46 to 92.92)
United States Virgin Islands	83.31	100.65	68.75	83.31<br>(68.75 to 100.65)
France	83.16	92.33	73.27	83.16<br>(73.27 to 92.33)
Low SDI	83.09	97.88	70.4	83.09<br>(70.4 to 97.88)
Ireland	83.04	91.28	74.24	83.04<br>(74.24 to 91.28)
Congo	82.99	100.95	68.88	82.99<br>(68.88 to 100.95)
Togo	82.92	106.76	63.27	82.92<br>(63.27 to 106.76)
Burkina Faso	82.55	105.11	65.36	82.55<br>(65.36 to 105.11)
Brunei Darussalam	82.5	108.96	51.8	82.5<br>(51.8 to 108.96)
Argentina	82.4	88.84	75.58	82.4<br>(75.58 to 88.84)
Liberia	82.37	105.49	60.77	82.37<br>(60.77 to 105.49)
India	82.31	95.13	69.34	82.31<br>(69.34 to 95.13)
Yemen	82.23	109.94	62.19	82.23<br>(62.19 to 109.94)
Benin	81.54	97.16	67.29	81.54<br>(67.29 to 97.16)
Mauritania	80.31	104.59	60.07	80.31<br>(60.07 to 104.59)
Mozambique	80.23	111.88	56.73	80.23<br>(56.73 to 111.88)
Algeria	80.08	95.92	66.95	80.08<br>(66.95 to 95.92)

Central African Republic	79.98	97.75	61.9	79.98<br>(61.9 to 97.75)
Tonga	79.97	108.32	62.04	79.97<br>(62.04 to 108.32)
Angola	79.77	98.9	63.53	79.77<br>(63.53 to 98.9)
El Salvador	79.65	92.84	67.44	79.65<br>(67.44 to 92.84)
Czechia	79.58	87.81	70.93	79.58<br>(70.93 to 87.81)
Slovenia	79.58	88.8	69.24	79.58<br>(69.24 to 88.8)
Switzerland	79.34	88.16	69.3	79.34<br>(69.3 to 88.16)
Paraguay	79.3	96.73	64.44	79.3<br>(64.44 to 96.73)
Italy	79.29	86.84	69.96	79.29<br>(69.96 to 86.84)
Sudan	79.13	101.2	64.03	79.13<br>(64.03 to 101.2)
Sierra Leone	78.84	97.12	63.2	78.84<br>(63.2 to 97.12)
Rwanda	78.75	104.5	54.2	78.75<br>(54.2 to 104.5)
Niger	78.13	97.37	62.95	78.13<br>(62.95 to 97.37)
Mexico	78.05	87.3	69.74	78.05<br>(69.74 to 87.3)
South Sudan	77.88	101.75	60.33	77.88<br>(60.33 to 101.75)
Australia	77.84	83.99	69.23	77.84<br>(69.23 to 83.99)
New Zealand	77.69	83.37	69.33	77.69<br>(69.33 to 83.37)
Jamaica	77.66	92.88	64.15	77.66<br>(64.15 to 92.88)
Ghana	77.32	92.3	63.12	77.32<br>(63.12 to 92.3)
Dominican Republic	77.1	94.87	63.13	77.1<br>(63.13 to 94.87)
Venezuela (Bolivarian Republic of)	76.97	93.24	63.43	76.97<br>(63.43 to 93.24)
Sweden	76.92	85.71	68.18	76.92<br>(68.18 to 85.71)

Philippines	76.87	87.67	66.61	76.87 (66.61 to 87.67)
Cuba	76.56	85.49	68.17	76.56 (68.17 to 85.49)
Latvia	76.54	84.43	68.33	76.54 (68.33 to 84.43)
Bangladesh	76.47	104.08	58.1	76.47 (58.1 to 104.08)
Mauritius	76.26	81.44	69.76	76.26 (69.76 to 81.44)
Comoros	76.23	101.63	56.47	76.23 (56.47 to 101.63)
Russian Federation	76.19	82.7	70.04	76.19 (70.04 to 82.7)
Burundi	75.92	100.07	48.92	75.92 (48.92 to 100.07)
Cook Islands	75.58	96.63	61.47	75.58 (61.47 to 96.63)
Estonia	75.53	84.08	67.26	75.53 (67.26 to 84.08)
Hungary	75.22	82.78	67.38	75.22 (67.38 to 82.78)
Uganda	75.14	96.15	53.42	75.14 (53.42 to 96.15)
Timor-Leste	74.46	91.76	59.41	74.46 (59.41 to 91.76)
Brazil	74.42	80.24	67.13	74.42 (67.13 to 80.24)
Panama	74.36	87.59	60.99	74.36 (60.99 to 87.59)
Georgia	73.79	81.2	66.19	73.79 (66.19 to 81.2)
Iran (Islamic Republic of)	73.79	83.31	45.29	73.79 (45.29 to 83.31)
Democratic Republic of the Congo	73.51	94.33	51.71	73.51 (51.71 to 94.33)
Lithuania	73.39	80.94	65.59	73.39 (65.59 to 80.94)
Portugal	73.37	79.83	65.94	73.37 (65.94 to 79.83)
Malawi	73.36	99.39	50.78	73.36 (50.78 to 99.39)
Tunisia	73.27	93.15	56.23	73.27 (56.23 to 93.15)

Ukraine	72.79	87.61	59.17	72.79<br>(59.17 to 87.61)
Sri Lanka	72.69	90.67	54.83	72.69<br>(54.83 to 90.67)
Andorra	72.67	92.06	55.78	72.67<br>(55.78 to 92.06)
Kenya	72.45	91.29	56.43	72.45<br>(56.43 to 91.29)
Trinidad and Tobago	72.41	86.32	59.81	72.41<br>(59.81 to 86.32)
Djibouti	71.34	102.4	52.05	71.34<br>(52.05 to 102.4)
Costa Rica	71.01	78.95	63.46	71.01<br>(63.46 to 78.95)
Puerto Rico	70.91	81.34	61.06	70.91<br>(61.06 to 81.34)
Colombia	70.59	80.84	61.27	70.59<br>(61.27 to 80.84)
Slovakia	70.38	79	61.5	70.38<br>(61.5 to 79)
South Africa	69.96	76.09	62.41	69.96<br>(62.41 to 76.09)
Zambia	69.79	91.64	53.94	69.79<br>(53.94 to 91.64)
Azerbaijan	69.12	80.32	58.7	69.12<br>(58.7 to 80.32)
Maldives	69.03	79.93	58.24	69.03<br>(58.24 to 79.93)
Somalia	68.52	93.41	45.98	68.52<br>(45.98 to 93.41)
United Republic of Tanzania	68.41	92.72	51.64	68.41<br>(51.64 to 92.72)
Republic of Korea	68.24	78.91	55.55	68.24<br>(55.55 to 78.91)
Botswana	67.92	81.87	57.76	67.92<br>(57.76 to 81.87)
Suriname	67.87	81.8	53.68	67.87<br>(53.68 to 81.8)
Bermuda	67.53	81.47	56.56	67.53<br>(56.56 to 81.47)
Lebanon	67.12	78.28	57.75	67.12<br>(57.75 to 78.28)
Jordan	66.34	79.01	55.37	66.34<br>(55.37 to 79.01)

Armenia	66.16	73.29	58.09	66.16<br>(58.09 to 73.29)
Turkmenistan	65.31	78.69	53.79	65.31<br>(53.79 to 78.69)
Kuwait	65.22	76.2	55.06	65.22<br>(55.06 to 76.2)
Guatemala	64.76	72.96	57.18	64.76<br>(57.18 to 72.96)
Nicaragua	64.24	73.81	54.19	64.24<br>(54.19 to 73.81)
Ethiopia	62.85	92.52	46.4	62.85<br>(46.4 to 92.52)
Mongolia	62.7	72.15	53.55	62.7<br>(53.55 to 72.15)
Uzbekistan	60.31	67.69	53.08	60.31<br>(53.08 to 67.69)
Japan	58.54	63.3	50.93	58.54<br>(50.93 to 63.3)
Madagascar	57.65	78.54	42.67	57.65<br>(42.67 to 78.54)
Republic of Moldova	56.8	62.63	51.56	56.8<br>(51.56 to 62.63)
Kyrgyzstan	56	63.93	48.76	56<br>(48.76 to 63.93)
Singapore	55.65	60.55	50.09	55.65<br>(50.09 to 60.55)
Guam	49.16	57.48	41.01	49.16<br>(41.01 to 57.48)
San Marino	43.51	54.95	33.07	43.51<br>(33.07 to 54.95)

Table S9: Incidence of PD by sex across GBD regions in 2021

location	sex	val	upper	lower	Number_2021
East Asia	Male	309394	361404	263136	309394<br>(263136 to 361404)
East Asia	Female	213705	248177	180960	213705<br>(180960 to 248177)
Oceania	Male	399	449	344	399<br>(344 to 449)
Oceania	Female	292	331	254	292<br>(254 to 331)
Southeast Asia	Male	36389	39994	33114	36389<br>(33114 to 39994)
Southeast Asia	Female	31214	34825	28156	31214<br>(28156 to 34825)

Central Asia	Male	4410	4950	3924	4410<br>(3924 to 4950)
Central Asia	Female	4304	4638	3943	4304<br>(3943 to 4638)
Southern Latin America	Male	7328	8216	6692	7328<br>(6692 to 8216)
Southern Latin America	Female	5567	6374	5042	5567<br>(5042 to 6374)
Central Europe	Male	14994	16243	13736	14994<br>(13736 to 16243)
Central Europe	Female	13548	14452	12579	13548<br>(12579 to 14452)
Western Europe	Male	97862	105102	90948	97862<br>(90948 to 105102)
Western Europe	Female	77620	84006	70963	77620<br>(70963 to 84006)
High-income North America	Male	63300	68354	58563	63300<br>(58563 to 68354)
High-income North America	Female	39545	42616	36710	39545<br>(36710 to 42616)
High-income Asia Pacific	Male	23431	26221	20861	23431<br>(20861 to 26221)
High-income Asia Pacific	Female	17509	19736	15525	17509<br>(15525 to 19736)
Central Latin America	Male	15920	17363	14541	15920<br>(14541 to 17363)
Central Latin America	Female	13095	14484	11849	13095<br>(11849 to 14484)
Eastern Europe	Male	18392	21118	15858	18392<br>(15858 to 21118)
Eastern Europe	Female	21046	23909	18260	21046<br>(18260 to 23909)
Australasia	Male	4100	4552	3781	4100<br>(3781 to 4552)
Australasia	Female	2610	3042	2237	2610<br>(2237 to 3042)
Central Sub-Saharan Africa	Male	2152	2491	1859	2152<br>(1859 to 2491)
Central Sub-Saharan Africa	Female	1708	1942	1498	1708<br>(1498 to 1942)
Eastern Sub-Saharan Africa	Male	7489	8248	6761	7489<br>(6761 to 8248)
Eastern Sub-Saharan Africa	Female	4996	5520	4490	4996<br>(4490 to 5520)

Andean Latin America	Male	5778	6634	5131	5778<br>(5131 to 6634)
Andean Latin America	Female	4010	4431	3588	4010<br>(3588 to 4431)
Caribbean	Male	2902	3132	2683	2902<br>(2683 to 3132)
Caribbean	Female	2436	2622	2262	2436<br>(2262 to 2622)
South Asia	Male	88346	100258	76980	88346<br>(76980 to 100258)
South Asia	Female	74281	84728	64394	74281<br>(64394 to 84728)
Tropical Latin America	Male	13824	15661	12021	13824<br>(12021 to 15661)
Tropical Latin America	Female	13477	15290	11648	13477<br>(11648 to 15290)
North Africa and Middle East	Male	33428	36795	30054	33428<br>(30054 to 36795)
North Africa and Middle East	Female	20952	23367	18753	20952<br>(18753 to 23367)
Southern Sub-Saharan Africa	Male	2405	2731	2100	2405<br>(2100 to 2731)
Southern Sub-Saharan Africa	Female	2467	2825	2139	2467<br>(2139 to 2825)
Western Sub-Saharan Africa	Male	9900	10887	8986	9900<br>(8986 to 10887)
Western Sub-Saharan Africa	Female	8616	9563	7691	8616<br>(7691 to 9563)

Table S10: ASIR of PD by sex across GBD regions in 2021

location	sex	val	upper	lower	Rate_2021
East Asia	Male	31.01	36.1	26.47	31.01<br>(26.47 to 36.1)
East Asia	Female	18.63	21.51	15.85	18.63<br>(15.85 to 21.51)
Oceania	Male	13.06	14.87	11.43	13.06<br>(11.43 to 14.87)
Oceania	Female	9.23	10.37	8.24	9.23<br>(8.24 to 10.37)
Southeast Asia	Male	13.82	15.05	12.65	13.82<br>(12.65 to 15.05)
Southeast Asia	Female	9.62	10.64	8.71	9.62<br>(8.71 to 10.64)
Western Europe	Male	22.12	23.7	20.63	22.12<br>(20.63 to 23.7)

Western Europe	Female	14.2	15.32	13.03	14.2 (13.03 to 15.32)
Southern Latin America	Male	19.26	21.52	17.67	19.26 (17.67 to 21.52)
Southern Latin America	Female	10.72	12.22	9.75	10.72 (9.75 to 12.22)
Central Asia	Male	15.3	16.69	13.84	15.3 (13.84 to 16.69)
Central Asia	Female	10.22	10.9	9.4	10.22 (9.4 to 10.9)
High-income North America	Male	20.43	22.04	18.89	20.43 (18.89 to 22.04)
High-income North America	Female	10.52	11.3	9.78	10.52 (9.78 to 11.3)
Central Europe	Male	15.52	16.76	14.36	15.52 (14.36 to 16.76)
Central Europe	Female	9.89	10.55	9.18	9.89 (9.18 to 10.55)
Central Latin America	Male	14.48	15.8	13.24	14.48 (13.24 to 15.8)
Central Latin America	Female	9.86	10.89	8.93	9.86 (8.93 to 10.89)
Eastern Sub-Saharan Africa	Male	11.41	12.53	10.34	11.41 (10.34 to 12.53)
Eastern Sub-Saharan Africa	Female	6.49	7.13	5.86	6.49 (5.86 to 7.13)
Australasia	Male	15.42	17.05	14.2	15.42 (14.2 to 17.05)
Australasia	Female	8.76	10.31	7.46	8.76 (7.46 to 10.31)
Caribbean	Male	11.79	12.68	10.9	11.79 (10.9 to 12.68)
Caribbean	Female	8.28	8.91	7.7	8.28 (7.7 to 8.91)
Andean Latin America	Male	20.86	23.85	18.63	20.86 (18.63 to 23.85)
Andean Latin America	Female	13.04	14.41	11.67	13.04 (11.67 to 14.41)
North Africa and Middle East	Male	16.83	18.53	15.21	16.83 (15.21 to 18.53)
North Africa and Middle East	Female	10.33	11.47	9.3	10.33 (9.3 to 11.47)
Central Sub-Saharan Africa	Male	11.31	12.6	10.12	11.31 (10.12 to 12.6)

Central Sub-Saharan Africa	Female	6.63	7.45	5.88	6.63<br>(5.88 to 7.45)
Tropical Latin America	Male	12.46	14.05	10.81	12.46<br>(10.81 to 14.05)
Tropical Latin America	Female	9.49	10.75	8.2	9.49<br>(8.2 to 10.75)
Western Sub-Saharan Africa	Male	13.16	14.47	11.98	13.16<br>(11.98 to 14.47)
Western Sub-Saharan Africa	Female	10.57	11.7	9.55	10.57<br>(9.55 to 11.7)
Eastern Europe	Male	14.33	16.18	12.46	14.33<br>(12.46 to 16.18)
Eastern Europe	Female	9.14	10.35	7.97	9.14<br>(7.97 to 10.35)
High-income Asia Pacific	Male	10.65	11.82	9.54	10.65<br>(9.54 to 11.82)
High-income Asia Pacific	Female	6.5	7.22	5.82	6.5<br>(5.82 to 7.22)
South Asia	Male	13.45	15.16	11.79	13.45<br>(11.79 to 15.16)
South Asia	Female	10.33	11.7	8.92	10.33<br>(8.92 to 11.7)
Southern Sub-Saharan Africa	Male	12.34	13.85	10.83	12.34<br>(10.83 to 13.85)
Southern Sub-Saharan Africa	Female	8.28	9.4	7.23	8.28<br>(7.23 to 9.4)

Table S11: Prevalence of PD by sex across GBD regions in 2021

location	sex	val	upper	lower	Number_2021
East Asia	Male	2986706	3547516	2510652	2986706<br>(2510652 to 3547516)
East Asia	Female	2225777	2622633	1910783	2225777<br>(1910783 to 2622633)
Southeast Asia	Male	269529	307765	235660	269529<br>(235660 to 307765)
Southeast Asia	Female	233421	270157	203190	233421<br>(203190 to 270157)
Oceania	Male	2727	3223	2267	2727<br>(2267 to 3223)
Oceania	Female	2218	2632	1822	2218<br>(1822 to 2632)
Central Europe	Male	110858	124018	97577	110858<br>(97577 to 124018)
Central Europe	Female	116956	127503	104987	116956<br>(104987 to 127503)

Eastern Europe	Male	144361	171035	120976	144361<br>(120976 to 171035)
Eastern Europe	Female	188212	221354	158823	188212<br>(158823 to 221354)
Central Asia	Male	28860	34174	24039	28860<br>(24039 to 34174)
Central Asia	Female	31448	35699	27282	31448<br>(27282 to 35699)
High-income Asia Pacific	Male	150994	174287	129659	150994<br>(129659 to 174287)
High-income Asia Pacific	Female	144713	166063	125085	144713<br>(125085 to 166063)
High-income North America	Male	496753	534633	462967	496753<br>(462967 to 534633)
High-income North America	Female	346594	371425	321971	346594<br>(321971 to 371425)
Western Europe	Male	809362	899718	732276	809362<br>(732276 to 899718)
Western Europe	Female	789962	863890	706968	789962<br>(706968 to 863890)
Australasia	Male	27351	32127	23734	27351<br>(23734 to 32127)
Australasia	Female	22691	28694	17860	22691<br>(17860 to 28694)
Southern Latin America	Male	52118	62814	43943	52118<br>(43943 to 62814)
Southern Latin America	Female	46348	54275	39987	46348<br>(39987 to 54275)
Caribbean	Male	20968	24276	18315	20968<br>(18315 to 24276)
Caribbean	Female	19150	21729	16612	19150<br>(16612 to 21729)
Andean Latin America	Male	56166	67875	46880	56166<br>(46880 to 67875)
Andean Latin America	Female	36479	42591	30726	36479<br>(30726 to 42591)
Central Latin America	Male	131380	149760	115538	131380<br>(115538 to 149760)
Central Latin America	Female	110183	126082	96928	110183<br>(96928 to 126082)
Tropical Latin America	Male	115604	138156	98176	115604<br>(98176 to 138156)
Tropical Latin America	Female	113375	132242	96199	113375<br>(96199 to 132242)

North Africa and Middle East	Male	243141	278459	210008	243141 (210008 to 278459)
North Africa and Middle East	Female	161243	188188	140696	161243 (140696 to 188188)
South Asia	Male	644373	768095	546038	644373 (546038 to 768095)
South Asia	Female	620843	736262	524712	620843 (524712 to 736262)
Central Sub-Saharan Africa	Male	14884	17961	12090	14884 (12090 to 17961)
Central Sub-Saharan Africa	Female	12906	15516	10561	12906 (10561 to 15516)
Western Sub-Saharan Africa	Male	65399	75024	57162	65399 (57162 to 75024)
Western Sub-Saharan Africa	Female	53096	61019	45649	53096 (45649 to 61019)
Eastern Sub-Saharan Africa	Male	50423	58192	43327	50423 (43327 to 58192)
Eastern Sub-Saharan Africa	Female	35630	41105	30473	35630 (30473 to 41105)
Southern Sub-Saharan Africa	Male	16684	19342	14240	16684 (14240 to 19342)
Southern Sub-Saharan Africa	Female	17386	20355	14785	17386 (14785 to 20355)

Table S12: ASPR of PD by sex across GBD regions in 2021

location	sex	val	upper	lower	Rate_2021
East Asia	Male	302.81	353.52	256.31	302.81 (256.31 to 353.52)
East Asia	Female	194.81	229.02	166.9	194.81 (166.9 to 229.02)
High-income Asia Pacific	Male	70.34	80.99	60.65	70.34 (60.65 to 80.99)
High-income Asia Pacific	Female	53.28	61.74	46.06	53.28 (46.06 to 61.74)
Southeast Asia	Male	102.92	117.09	90.95	102.92 (90.95 to 117.09)
Southeast Asia	Female	72.45	83.82	62.98	72.45 (62.98 to 83.82)
Oceania	Male	93.08	109.31	75.05	93.08 (75.05 to 109.31)

Oceania	Female	73.33	86.91	59.5	73.33 (59.5 to 86.91)
Central Europe	Male	115.89	129.26	102.38	115.89 (102.38 to 129.26)
Central Europe	Female	81.85	89.73	73.77	81.85 (73.77 to 89.73)
High-income North America	Male	161.05	172.47	150.7	161.05 (150.7 to 172.47)
High-income North America	Female	89.26	95.63	82.88	89.26 (82.88 to 95.63)
Central Asia	Male	100.61	119.36	83.6	100.61 (83.6 to 119.36)
Central Asia	Female	76.68	86.38	66.43	76.68 (66.43 to 86.38)
Eastern Europe	Male	115.33	136.72	96.74	115.33 (96.74 to 136.72)
Eastern Europe	Female	79.82	93.16	67.55	79.82 (67.55 to 93.16)
Western Europe	Male	180.27	200.34	163.5	180.27 (163.5 to 200.34)
Western Europe	Female	129.69	141.39	116.83	129.69 (116.83 to 141.39)
Caribbean	Male	84.12	97.1	73.65	84.12 (73.65 to 97.1)
Caribbean	Female	65.56	74.41	56.72	65.56 (56.72 to 74.41)
Southern Latin America	Male	138.22	166.95	116.87	138.22 (116.87 to 166.95)
Southern Latin America	Female	86.55	100.37	74.92	86.55 (74.92 to 100.37)
Australasia	Male	102.32	120.05	88.93	102.32 (88.93 to 120.05)
Australasia	Female	72.98	91.64	57.59	72.98 (57.59 to 91.64)
Andean Latin America	Male	204.06	247.12	170.64	204.06 (170.64 to

					247.12)
Andean Latin America	Female	118.91	139.05	100.25	118.91 (100.25 to 139.05)
Central Latin America	Male	118.83	134.83	104.77	118.83 (104.77 to 134.83)
Central Latin America	Female	82.85	94.75	72.67	82.85 (72.67 to 94.75)
South Asia	Male	98.15	115.3	82.78	98.15 (82.78 to 115.3)
South Asia	Female	87.55	103.15	73.81	87.55 (73.81 to 103.15)
North Africa and Middle East	Male	126.11	144.29	107.84	126.11 (107.84 to 144.29)
North Africa and Middle East	Female	80.05	93.06	69.38	80.05 (69.38 to 93.06)
Central Sub-Saharan Africa	Male	75.3	91.45	60.87	75.3 (60.87 to 91.45)
Central Sub-Saharan Africa	Female	51.04	61.73	41.75	51.04 (41.75 to 61.73)
Tropical Latin America	Male	103.07	122.65	87.51	103.07 (87.51 to 122.65)
Tropical Latin America	Female	79.92	93.3	67.75	79.92 (67.75 to 93.3)
Eastern Sub-Saharan Africa	Male	74.47	84.87	64.02	74.47 (64.02 to 84.87)
Eastern Sub-Saharan Africa	Female	46.56	53.61	39.86	46.56 (39.86 to 53.61)
Southern Sub-Saharan Africa	Male	84.32	98.38	71.19	84.32 (71.19 to 98.38)
Southern Sub-Saharan Africa	Female	58.38	68.81	49.17	58.38 (49.17 to 68.81)
Western Sub-Saharan Africa	Male	84.38	96.78	72.94	84.38 (72.94 to 96.78)
Western Sub-Saharan Africa	Female	65.22	74.59	55.42	65.22 (55.42 to 74.59)

Table S13: Mortality rate of PD by sex across GBD regions in 2021

location	sex	val	upper	lower	Number_2021
East Asia	Male	56520	70020	45362	56520 (45362 to 70020)

East Asia	Female	39089	48432	30795	39089<br>(30795 to 48432)
Central Europe	Male	5915	6275	5458	5915<br>(5458 to 6275)
Central Europe	Female	6197	6715	5546	6197<br>(5546 to 6715)
Caribbean	Male	1455	1621	1295	1455<br>(1295 to 1621)
Caribbean	Female	1057	1186	923	1057<br>(923 to 1186)
Eastern Sub-Saharan Africa	Male	2272	3247	1782	2272<br>(1782 to 3247)
Eastern Sub-Saharan Africa	Female	2125	2749	1472	2125<br>(1472 to 2749)
North Africa and Middle East	Male	9761	10775	8624	9761<br>(8624 to 10775)
North Africa and Middle East	Female	6775	7617	5694	6775<br>(5694 to 7617)
Central Latin America	Male	5517	6113	4856	5517<br>(4856 to 6113)
Central Latin America	Female	4161	4593	3616	4161<br>(3616 to 4593)
High-income North America	Male	25452	26906	22515	25452<br>(22515 to 26906)
High-income North America	Female	16058	17600	13059	16058<br>(13059 to 17600)
Western Sub-Saharan Africa	Male	3651	4340	2997	3651<br>(2997 to 4340)
Western Sub-Saharan Africa	Female	3208	3688	2565	3208<br>(2565 to 3688)
Southeast Asia	Male	12648	14779	11152	12648<br>(11152 to 14779)
Southeast Asia	Female	12036	14951	10244	12036<br>(10244 to 14951)
Eastern Europe	Male	5961	6546	5368	5961<br>(5368 to 6546)
Eastern Europe	Female	9282	10217	8295	9282<br>(8295 to 10217)
Central Asia	Male	1187	1290	1070	1187<br>(1070 to 1290)
Central Asia	Female	1213	1329	1090	1213<br>(1090 to 1329)
South Asia	Male	30110	37117	23219	30110<br>(23219 to 37117)

South Asia	Female	23340	28754	18005	23340<br>(18005 to 28754)
Southern Sub-Saharan Africa	Male	839	905	760	839<br>(760 to 905)
Southern Sub-Saharan Africa	Female	964	1070	829	964<br>(829 to 1070)
High-income Asia Pacific	Male	12375	13139	10934	12375<br>(10934 to 13139)
High-income Asia Pacific	Female	10116	11799	7390	10116<br>(7390 to 11799)
Oceania	Male	128	171	99	128<br>(99 to 171)
Oceania	Female	90	116	70	90<br>(70 to 116)
Western Europe	Male	34016	35956	30311	34016<br>(30311 to 35956)
Western Europe	Female	22989	25465	18448	22989<br>(18448 to 25465)
Southern Latin America	Male	2702	2857	2502	2702<br>(2502 to 2857)
Southern Latin America	Female	1757	1889	1526	1757<br>(1526 to 1889)
Andean Latin America	Male	1687	2027	1395	1687<br>(1395 to 2027)
Andean Latin America	Female	1165	1390	953	1165<br>(953 to 1390)
Tropical Latin America	Male	5217	5519	4754	5217<br>(4754 to 5519)
Tropical Latin America	Female	4899	5355	4104	4899<br>(4104 to 5355)
Central Sub-Saharan Africa	Male	667	902	503	667<br>(503 to 902)
Central Sub-Saharan Africa	Female	691	907	483	691<br>(483 to 907)
Australasia	Male	1875	1997	1681	1875<br>(1681 to 1997)
Australasia	Female	1029	1143	827	1029<br>(827 to 1143)

Table S14: ASMR of PD by sex across GBD regions in 2021

location	sex	val	upper	lower	Rate_2021
East Asia	Male	7.24	8.83	5.83	7.24<br>(5.83 to 8.83)
East Asia	Female	3.59	4.44	2.82	3.59<br>(2.82 to 4.44)

Central Europe	Male	6.59	6.99	6.07	6.59<br>(6.07 to 6.99)
Central Europe	Female	3.93	4.26	3.53	3.93<br>(3.53 to 4.26)
Caribbean	Male	6.11	6.8	5.44	6.11<br>(5.44 to 6.8)
Caribbean	Female	3.46	3.88	3.03	3.46<br>(3.03 to 3.88)
Eastern Sub-Saharan Africa	Male	4.68	6.9	3.63	4.68<br>(3.63 to 6.9)
Eastern Sub-Saharan Africa	Female	3.59	4.73	2.48	3.59<br>(2.48 to 4.73)
North Africa and Middle East	Male	6.18	6.86	5.44	6.18<br>(5.44 to 6.86)
North Africa and Middle East	Female	4.13	4.66	3.48	4.13<br>(3.48 to 4.66)
Central Latin America	Male	5.53	6.11	4.86	5.53<br>(4.86 to 6.11)
Central Latin America	Female	3.25	3.58	2.82	3.25<br>(2.82 to 3.58)
High-income North America	Male	8.51	9.01	7.49	8.51<br>(7.49 to 9.01)
High-income North America	Female	3.75	4.08	3.09	3.75<br>(3.09 to 4.08)
Western Sub-Saharan Africa	Male	6.3	7.36	5.2	6.3<br>(5.2 to 7.36)
Western Sub-Saharan Africa	Female	5.04	5.77	4.06	5.04<br>(4.06 to 5.77)
Southeast Asia	Male	6.28	7.35	5.48	6.28<br>(5.48 to 7.35)
Southeast Asia	Female	4.29	5.32	3.62	4.29<br>(3.62 to 5.32)
Eastern Europe	Male	5.51	6.05	4.96	5.51<br>(4.96 to 6.05)
Eastern Europe	Female	3.62	3.99	3.24	3.62<br>(3.24 to 3.99)
Central Asia	Male	5.53	5.98	5.02	5.53<br>(5.02 to 5.98)
Central Asia	Female	3.23	3.54	2.89	3.23<br>(2.89 to 3.54)
South Asia	Male	5.75	7.04	4.46	5.75<br>(4.46 to 7.04)
South Asia	Female	3.83	4.74	2.93	3.83<br>(2.93 to 4.74)

Southern Sub-Saharan Africa	Male	5.74	6.15	5.22	5.74 (5.22 to 6.15)
Southern Sub-Saharan Africa	Female	3.72	4.1	3.16	3.72 (3.16 to 4.1)
High-income Asia Pacific	Male	5	5.3	4.44	5 (4.44 to 5.3)
High-income Asia Pacific	Female	2.62	3.01	2	2.62 (2 to 3.01)
Oceania	Male	5.52	7.49	4.3	5.52 (4.3 to 7.49)
Oceania	Female	3.92	4.97	3.07	3.92 (3.07 to 4.97)
Western Europe	Male	7.01	7.4	6.26	7.01 (6.26 to 7.4)
Western Europe	Female	3.15	3.46	2.59	3.15 (2.59 to 3.46)
Southern Latin America	Male	7.58	8.02	7	7.58 (7 to 8.02)
Southern Latin America	Female	3.06	3.29	2.68	3.06 (2.68 to 3.29)
Andean Latin America	Male	6.78	8.12	5.61	6.78 (5.61 to 8.12)
Andean Latin America	Female	3.88	4.63	3.18	3.88 (3.18 to 4.63)
Tropical Latin America	Male	5.38	5.7	4.87	5.38 (4.87 to 5.7)
Tropical Latin America	Female	3.45	3.77	2.9	3.45 (2.9 to 3.77)
Central Sub-Saharan Africa	Male	5.56	7.9	4.16	5.56 (4.16 to 7.9)
Central Sub-Saharan Africa	Female	3.74	4.94	2.54	3.74 (2.54 to 4.94)
Australasia	Male	6.88	7.32	6.17	6.88 (6.17 to 7.32)
Australasia	Female	2.93	3.24	2.38	2.93 (2.38 to 3.24)

Table S15: DALYs of PD by sex across GBD regions in 2021

location	sex	val	upper	lower	Number_2021
Central Europe	Male	103980	111706	96225	103980 (96225 to 111706)
Central Europe	Female	101721	109289	92506	101721 (92506 to 109289)
Caribbean	Male	25192	27927	22556	25192 (22556 to 27927)

Caribbean	Female	17830	19876	15885	17830<br>(15885 to 19876)
Western Europe	Male	570769	614381	521277	570769<br>(521277 to 614381)
Western Europe	Female	397167	439331	341742	397167<br>(341742 to 439331)
North Africa and Middle East	Male	190586	210590	169975	190586<br>(169975 to 210590)
North Africa and Middle East	Female	132187	147504	114030	132187<br>(114030 to 147504)
Southeast Asia	Male	254038	291676	226326	254038<br>(226326 to 291676)
Southeast Asia	Female	220755	265975	191993	220755<br>(191993 to 265975)
Oceania	Male	2811	3633	2210	2811<br>(2210 to 3633)
Oceania	Female	1991	2549	1552	1991<br>(1552 to 2549)
High-income North America	Male	428576	455831	390417	428576<br>(390417 to 455831)
High-income North America	Female	260022	283645	223486	260022<br>(223486 to 283645)
Central Latin America	Male	103886	116105	92936	103886<br>(92936 to 116105)
Central Latin America	Female	76926	86000	68602	76926<br>(68602 to 86000)
Central Sub-Saharan Africa	Male	14906	18938	11541	14906<br>(11541 to 18938)
Central Sub-Saharan Africa	Female	14133	17913	10371	14133<br>(10371 to 17913)
Eastern Sub-Saharan Africa	Male	47680	63693	38672	47680<br>(38672 to 63693)
Eastern Sub-Saharan Africa	Female	40403	51132	29560	40403<br>(29560 to 51132)
Central Asia	Male	23631	26122	21373	23631<br>(21373 to 26122)
Central Asia	Female	23235	25422	21152	23235<br>(21152 to 25422)
Eastern Europe	Male	114341	127085	103020	114341<br>(103020 to 127085)
Eastern Europe	Female	158391	174527	141991	158391<br>(141991 to 174527)
Australasia	Male	29745	31714	27180	29745<br>(27180 to 31714)

Australasia	Female	16717	18501	14131	16717 (14131 to 18501)
South Asia	Male	590828	710278	467761	590828 (467761 to 710278)
South Asia	Female	464121	554586	372737	464121 (372737 to 554586)
Southern Latin America	Male	46586	49782	43247	46586 (43247 to 49782)
Southern Latin America	Female	30363	33072	27360	30363 (27360 to 33072)
Andean Latin America	Male	33225	39164	27844	33225 (27844 to 39164)
Andean Latin America	Female	21858	25368	18595	21858 (18595 to 25368)
East Asia	Male	1318423	1587108	1092861	1318423 (1092861 to 1587108)
East Asia	Female	915910	1090996	750099	915910 (750099 to 1090996)
Tropical Latin America	Male	97361	104636	89436	97361 (89436 to 104636)
Tropical Latin America	Female	85174	93125	75158	85174 (75158 to 93125)
High-income Asia Pacific	Male	188846	201985	172445	188846 (172445 to 201985)
High-income Asia Pacific	Female	147735	168774	117383	147735 (117383 to 168774)
Southern Sub-Saharan Africa	Male	17060	18530	15528	17060 (15528 to 18530)
Southern Sub-Saharan Africa	Female	16948	18749	14630	16948 (14630 to 18749)
Western Sub-Saharan Africa	Male	69523	82219	57313	69523 (57313 to 82219)
Western Sub-Saharan Africa	Female	56240	64859	45933	56240 (45933 to 64859)

Table S16: ASDR of PD by sex across GBD regions in 2021

location	sex	val	upper	lower	Rate_2021
Central Europe	Male	111.18	119.32	103	111.18 (103 to 119.32)
Central Europe	Female	67.98	73.09	62.05	67.98 (62.05 to 73.09)
Caribbean	Male	103.48	114.58	92.71	103.48 (92.71 to 114.58)
Caribbean	Female	60.07	66.92	53.57	60.07 (53.57 to 66.92)

Western Europe	Male	121.89	131.48	111.44	121.89<br>(111.44 to 131.48)
Western Europe	Female	61.93	68.42	53.96	61.93<br>(53.96 to 68.42)
North Africa and Middle East	Male	106.46	117.38	95.01	106.46<br>(95.01 to 117.38)
North Africa and Middle East	Female	71.35	79.15	61.06	71.35<br>(61.06 to 79.15)
Southeast Asia	Male	108.18	124.6	96.09	108.18<br>(96.09 to 124.6)
Southeast Asia	Female	72.8	88.22	63.15	72.8<br>(63.15 to 88.22)
Oceania	Male	100.76	131.13	79.86	100.76<br>(79.86 to 131.13)
Oceania	Female	73.38	92.81	57.92	73.38<br>(57.92 to 92.81)
High-income North America	Male	140.49	149.57	127.53	140.49<br>(127.53 to 149.57)
High-income North America	Female	64.61	70.28	56.03	64.61<br>(56.03 to 70.28)
Central Latin America	Male	98.65	110.09	88.12	98.65<br>(88.12 to 110.09)
Central Latin America	Female	59.02	65.94	52.55	59.02<br>(52.55 to 65.94)
Central Sub-Saharan Africa	Male	94.75	126.1	73.56	94.75<br>(73.56 to 126.1)
Central Sub-Saharan Africa	Female	64.37	81.94	46.64	64.37<br>(46.64 to 81.94)
Eastern Sub-Saharan Africa	Male	81.37	112.33	65.38	81.37<br>(65.38 to 112.33)
Eastern Sub-Saharan Africa	Female	59.71	75.87	42.99	59.71<br>(42.99 to 75.87)
Central Asia	Male	94.68	104.06	86.2	94.68<br>(86.2 to 104.06)
Central Asia	Female	59.07	64.54	53.84	59.07<br>(53.84 to 64.54)
Eastern Europe	Male	97.94	108.3	88.73	97.94<br>(88.73 to 108.3)
Eastern Europe	Female	64.57	71.13	58.06	64.57<br>(58.06 to 71.13)
Australasia	Male	109.75	117.2	100.33	109.75<br>(100.33 to 117.2)
Australasia	Female	51.28	56.64	43.89	51.28<br>(43.89 to 56.64)

South Asia	Male	98.88	118.07	78.59	98.88 (78.59 to 118.07)
South Asia	Female	69.65	83.27	55.62	69.65 (55.62 to 83.27)
Southern Latin America	Male	126.05	134.68	117.04	126.05 (117.04 to 134.68)
Southern Latin America	Female	55.47	60.39	50.08	55.47 (50.08 to 60.39)
Andean Latin America	Male	126.8	149.67	106.49	126.8 (106.49 to 149.67)
Andean Latin America	Female	72.35	84.03	61.54	72.35 (61.54 to 84.03)
East Asia	Male	143.97	171.49	120.68	143.97 (120.68 to 171.49)
East Asia	Female	80.8	95.98	66.16	80.8 (66.16 to 95.98)
Tropical Latin America	Male	93.65	100.4	86.03	93.65 (86.03 to 100.4)
Tropical Latin America	Female	60.23	65.85	53.15	60.23 (53.15 to 65.85)
High-income Asia Pacific	Male	80.58	86.48	73.82	80.58 (73.82 to 86.48)
High-income Asia Pacific	Female	45.47	51.16	37.56	45.47 (37.56 to 51.16)
Southern Sub-Saharan Africa	Male	96.2	103.48	87.38	96.2 (87.38 to 103.48)
Southern Sub-Saharan Africa	Female	60.01	66.14	51.64	60.01 (51.64 to 66.14)
Western Sub-Saharan Africa	Male	101.74	119.6	84.93	101.74 (84.93 to 119.6)
Western Sub-Saharan Africa	Female	77.92	88.69	64	77.92 (64 to 88.69)
